# Self-relabeling for noise-tolerant retina vessel segmentation through label reliability estimation

**DOI:** 10.1186/s12880-021-00732-y

**Published:** 2022-01-12

**Authors:** Jiacheng Li, Ruirui Li, Ruize Han, Song Wang

**Affiliations:** 1 College of Intelligence and Computing, Tianjin University, Tianjin, China; 2 College of Information Science and Technology, Beijing University of Chemical Technology, Beijing, China; 3 Department of Computer Science and Engineering, University of South Carolina, Columbia, USA

**Keywords:** Retina image segmentation, Label map correction, Noise-tolerant, Reliability estimation, Temporal statistics

## Abstract

**Background:**

Retinal vessel segmentation benefits significantly from deep learning. Its performance relies on sufficient training images with accurate ground-truth segmentation, which are usually manually annotated in the form of binary pixel-wise label maps. Manually annotated ground-truth label maps, more or less, contain errors for part of the pixels. Due to the thin structure of retina vessels, such errors are more frequent and serious in manual annotations, which negatively affect deep learning performance.

**Methods:**

In this paper, we develop a new method to automatically and iteratively identify and correct such noisy segmentation labels in the process of network training. We consider historical predicted label maps of network-in-training from different epochs and jointly use them to self-supervise the predicted labels during training and dynamically correct the supervised labels with noises.

**Results:**

We conducted experiments on the three datasets of DRIVE, STARE and CHASE-DB1 with synthetic noises, pseudo-labeled noises, and manually labeled noises. For synthetic noise, the proposed method corrects the original noisy label maps to a more accurate label map by 4.0–$9.8\%$ on $F_1$ and 10.7–$16.8\%$ on PR on three testing datasets. For the other two types of noise, the method could also improve the label map quality.

**Conclusions:**

Experiment results verified that the proposed method could achieve better retinal image segmentation performance than many existing methods by simultaneously correcting the noise in the initial label map.

**Supplementary Information:**

The online version contains supplementary material available at 10.1186/s12880-021-00732-y.

## Background

Retinal fundus images as an essential kind of medical image are widely used in the early screening and diagnosis of ophthalmologic diseases. Segmenting blood vessels from the retinal fundus image is important for the automatic detection of fundus retinopathy and has drawn much interest in recent years. With the development of deep learning in analyzing medical images, researchers have proposed many effective deep learning-based methods such as [1–3]. Most of them rely on supervised learning strategies that require a large number of training samples with accurate annotations to obtain a well-learned model. However, because of the thin structure of retina vessels and the high accuracy requirements of the dense pixel labels, retina vessel segmentation labels rely on professional clinical ophthalmologists to annotate the retinal fundus images pixel by pixel, which is a time-consuming, laborious, and expensive work. This severely limits deep learning models’ wide application in actual auxiliary diagnosis. To tackle this bottleneck, researchers try to relax the restrictions on label accuracy. They adopt more economical methods of obtaining labels, such as hiring junior medical staff to annotate, crowdsourcing, or pseudo labeling. All the above methods for obtaining cheap yet noisy label maps on a new unlabeled dataset come up with the same problem: *How to fully utilize the correct labels in the noisy label maps to train the model while defending the bad effect from noisy labels to the training?*

This problem is named as learning with noisy labels (LNL) in many works [4, 5]. Existing methods on LNL are mainly designed for the classification tasks on natural images [4–8]. Among them, Co-teaching [7] is a simple yet effective strategy that uses the agreement of the predictions from two differently initialized networks to select potential correct labels from the low-quality label sets to train the model. Tanaka et al. [5] proposed a framework on LNL which jointly optimizes the network parameters and estimates true labels. Though most of these methods could not be directly applied to the semantic segmentation tasks due to the dense prediction pattern in segmentation, they inspired many methods on LNL in the segmentation tasks [9–12]. Among these methods, Li et al. [12] proposed a robust framework that could progressively prompt the quality of the labels as well as the learned models. It corrects the noisy labels by iteratively aggregating the current network prediction with the initial noisy labels through a moving average strategy. Nevertheless, the framework proposed by Li et al. [12] directly uses the smoothed prediction values to modify the labels. This method may also mistakenly correct the labels, leading to further accumulation of errors in the subsequent training process. To avoid accumulating errors, Liu [13] et al. utilized a mutual learning strategy to estimate the reliability of the labels. In medical image segmentation, Xue et al. [11] and Zhang et al. [14] proposed two similar mutual learning frameworks which train three networks simultaneously and treat the agreement of two networks as clean labels to train another network. Though the mutual learning strategy could fully utilize the random initialization of different network parameters, it costs high GPU memory and computation to train multiple networks at the same time. In real applications, a more flexible and lightweight noise-tolerant solution is desired for medical image segmentation.

The critical problem in designing such a method is evaluating the accuracy between the predicted labels trained on noisy labels and the given noisy labels themselves. One basic assumption in many studies based on consistency and regularization [15, 16] is that: in the process of deep model training, there will be multiple periods of random exploration. The correct label is more steadily close to the predicted value among these periods. Inspired by this point of view, we propose a joint framework for the noise-tolerant retinal vessel segmentation task that simultaneously trains the network and corrects the noisy labels. The framework combines the advantages of Li et al. [12] to update annotations efficiently and iteratively. Differently, we propose an estimation method for the reliability of both labels and predictions. Based on this estimation, we construct a time memory loss for robust training and a label correction compensation mechanism for more accurate label correction. To verify the method proposed in this paper, we conduct experiments on three public retinal blood vessel data sets and analyze the model’s accuracy under three different types of noise: synthetic noises, pseudo-labeled noises, and manually labeled noises. The results show that the proposed method can still effectively maintain the accuracy of blood vessel segmentation under a large proportion of noise without the help of additional true labels.

In summary, we make the following contributions in this paper:

- An efficient framework for noise-tolerant retinal vessel segmentation that can estimate the reliability of both the labels and the predictions;
- a temporal memory loss for robust training;
- a label correction compensation mechanism for more accurate label correction.

## Related works

Retina vessel segmentation is a task with long studying history [17] and quite a lot of mature methods [18]. Beneficial from the development of deep learning, the current SOTA methods [19, 20] have achieved fairly accurate prediction results on the widely used public datasets, such as DRIVE [17], STARE [21], CHASE [22]. However, seldom of them focus on how to eliminate the noisy label map caused by reasons like observer variety, which could degrade the segmentation accuracy [23]. In this work, we aimed to rectify the noisy label map and improve the segmentation accuracy in the meantime.

Rectifying segmentation label map is a branch of studies of learning from noisy labels [24] (LNL). Since datasets with both noisy labels and carefully-checked clean labels, e.g., WebVision [25], only provide data and evaluation for LNL of classification task, existing studies of LNL mainly focus on the classification task. Some of them studied the task of reducing the bad effect of noisy labels on the network by reweighting the noisy labels in loss functions [4, 7, 26] or dropping the noisy labeled samples in data sampling [27, 28]. To distinguish the noisy labels from all the labels, strategies like generative learning [29, 30], contrastive learning [31], entropy minimization [32], consistency regularization [33, 34] and pseudo labeling [35] are widely used and developed to many variants. These strategies also inspired many recent works on LNL of segmentation tasks. Unlike classification, segmentation is a dense prediction task. Even pixel-wise noisy labels have contextual information with their neighbor pixels, which is not suitable for reweighting or dropping them independently. In recent years, many studies [36, 37] focused on semi-supervised LNL on segmentation. However, they still need clean labels to provide essential information on distinguishing noisy labels. In this work, we are targeted at the task of unsupervised rectifying noisy label maps in retina vessel image segmentation, which could only provide noisy label maps with position-unknown clean labels.

Existed unsupervised segmentation label map rectify methods are mainly based on strategies like consistency regularization [11] and pseudo labeling [11, 12]. Xue et al. [11] proposed a framework that could correct the noisy boundary annotations without knowing clean annotations on chest X-ray images. Inspired by the ideas of Co-teaching [7], they jointly trained three independent networks and treated the agreement of each two networks as correct annotations for the other one’s training. However, since the three networks share the same architecture and input, they may end up learning homogeneous knowledge and suffer from coupled noises that hinder the further improvement of label map [36]. Li et al. [12] studied the same task but on natural image datasets. They proposed a framework that directly uses the network’s prediction label map to change the supervised label maps iteratively. However, the training of the network is still affected by the noisy label maps and the correctness of the label map changes is hard to guarantee, highly relying on the network’s predicted label map accuracy. Our work is based on Li et al. [12] but with important improvements on both training with noisy label maps and distinguishing incorrect label map changes.

## Methods

### Overview

**Fig. 1:**
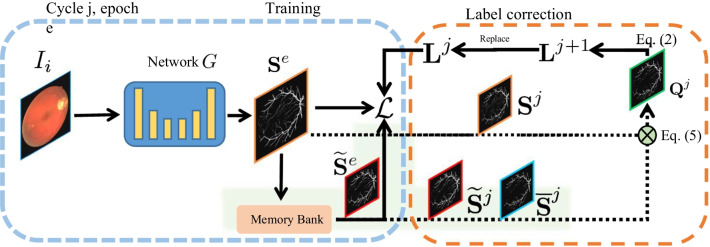
An illustration of the proposed framework

Given the retina vessel images and segmentation label maps with error pixel-wise labels, we aim to train a segmentation model with them and simultaneously correct the errors in the noisy label maps. We illustrate the pipeline of our method in Fig. 1, which contains two modules.

- *Segmentation training module (STM)* *G* denotes the segmentation network, which is trained for *C* *cycles* (each cycle contains *E* *epochs*) on the training set with the following loss
  1 $$\begin{aligned} {\mathcal {L}} = E({\mathbf{S }},{\mathbf{L}} ) \end{aligned}$$
  where *E* is the criterion loss function, ${\mathbf{S }}$ and ${\mathbf{L}}$ denote the predicted segmentation label map generated by *G* and the supervised label maps, respectively.
- *Label correction module (LCM)* After each cycle of training, we correct the given label maps (with noises). Specifically, inspired by [12], we consider the current label correction compensation ${\mathbf{Q }}^{j}$ in each cycle *j* and the initial label maps ${\mathbf{L}} ^{0}$ for updating the current corrected label maps in cycle *j*
  2 $$\begin{aligned} \begin{aligned} {\mathbf{L}} ^{j+1} = \frac{1}{j+1} \cdot {\mathbf{L}} ^{0} +\frac{j}{j+1}\cdot {\mathbf{Q }}^{j}, \ j=1,2,\ldots , M \end{aligned} \end{aligned}$$

which is used for training *G* at the $(j+1)$-th cycle. Specially, the label maps of cycle 1 is also equal to ${\mathbf{L}} ^{0}$. The details of the above two modules will be discussed in the following.

### Temporal memory loss (TML) for training

Since the initial label map ${\mathbf{L}} ^{0}$ is with noises, we aim to find a more accurate label map as supervision in training *G*. The key problem lies in estimating the current label maps ${\mathbf{L}}$ in Eq. (1) in each cycle. A straightforward idea is to use the updated label map ${\mathbf{L}} ^{j}$ as ${\mathbf{Q}} ^{j}$ in Eq. (2) like most previous works [12]. However, the updated label map cannot be considered completely accurate, especially in the early training cycles. In this work, we propose a temporal memory mechanism for improving the robustness of the supervision during training.

Specifically, while training the network *G* in the cycle *j*, we record the historical segmentation prediction ${\mathbf{S}} ^{e}$ at each epoch *e* and calculate the best *pixel-wise* predictions of this cycle. For example, at *e*-th epoch in cycle *j*, the best historical prediction at each pixel (*x*, *y*) is defined as

3 $$\begin{aligned} \widetilde{S}^e_{x,y}={S}^{k}_{x,y}, \quad {\mathrm {with}} \quad k=\mathop {\arg \min }\limits _{u}|{S}^{u}_{x,y}-L^{j}_{x,y}|, \end{aligned}$$

where $u \in \{1,2,\ldots ,e\}$ denote the epoch index in cycle *j*, and ${S}^{u}_{x,y}$ and $L^{j}_{x,y}$ denote the value at the pixel (*x*, *y*) on ${\mathbf{S}} ^{u}$ and ${\mathbf{L}} ^{j}$, respectively. We then combine $\widetilde{S}^e_{x,y}$ by all pixels and get the best historical prediction $\widetilde{\mathbf{S }}^{e}$. For the next epoch $e+1$ in this cycle, we replace the loss function in Eq. (1) with

4 $$\begin{aligned} {\mathcal {L}} = E\left( {\mathbf{S}} ^e, \widetilde{{\mathbf{S} }}^{e}\right) +\lambda \cdot E\left( {\mathbf{S}} ^e,{\mathbf{L}} ^{j}\right) \end{aligned}$$

where $\lambda$ is a preset weight and set as 0.1.

We explain the rationale of the proposed unsupervised loss. On the one hand, if the given label $L^{j}_{x,y}$ on pixel (*x*, *y*) is correct, the $\widetilde{S}^{e}_{x,y}$ will always be better than the prediction $S^e_{x,y}$ and guide the optimization in the ideal direction. On the other hand, if the label $L^{j}_{x,y}$ is incorrect, the historically learned $\widetilde{S}^{e}_{x,y}$ is less noisy than the label $L^{j}_{x,y}$, this manner could reduce the bad effect of the noisy label. This is because the network pretends to learn simple patterns first [23], and here the correct (pixel-wise) labels often have more consistent and simple patterns to learn than the various noisy labels.

**Fig. 2:**
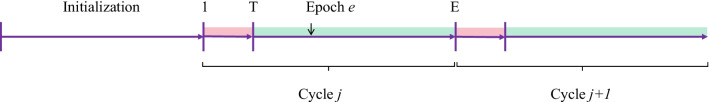
An illustration of the proposed training schedule

In the following, we discuss the details of the training as illustrated in Fig. 2. We first train the network with initial noisy label maps ${\mathbf{L}} ^{0}$ for several epochs as initialization following by multiple cycles of training. At the beginning of each cycle, we train the network for *T* epochs only consider the second item of Eq. (4) without the weight as loss function. This is because the recorded historical best prediction used in the first term in Eq. (4) needs several epochs to accumulate. After that, we train the network for next $E-T$ epochs using the loss defined in Eq. (4).

### Spatial confidence aware label correction

In this section, we discuss the label map correction strategy in Eq. (2), particularly for the label correction compensation ${\mathbf{Q }}^{j}$. Previous works [12] directly use the final predicted segmentation map in cycle *j* namely $\mathbf{S ^{j}}$ as $\mathbf{Q }^{j}$, which may be incredible because of under-fitted training and noisy-label supervision. While only using the $\widetilde{\mathbf{S }}^{j}$ as $\mathbf{Q }^{j}$ is not always the best, since the $\widetilde{\mathbf{S }}^{j}_{x,y}$ will be worse than $\mathbf{S} ^{j}_{x,y}$ at the pixels guided by the incorrect label $L^{j}_{x,y}$. In this work, we propose a spatial confidence aware label correction strategy to obtain a more reliable $\mathbf{Q }^{j}$ from the predicted segmentation maps. Specifically, we estimate the uncertainty of the prediction by the difference between its historical best and worst predictions, which could be formulated as $d^{j}_{x,y}=|\widetilde{S}^{j}_{x,y}-\overline{S}^{j}_{x,y}|$. Here, $\widetilde{S}^{j}_{x,y}$ is computed as Eq. (2) by taking the results of the last epoch in cycle *j*. Similarly, we also record the worst prediction $\overline{S}^{j}_{x,y}$ by replacing the minimum in Eq. (3) with the maximum operation. This way, $d^{j}_{x,y}$ can be taken as the rangeability of the historical prediction results, which contrary reflects its confidence at each pixel. Based on this, we replace the final prediction $\mathbf{S} ^{j}$ with $\widetilde{\mathbf{S }}^{j}$ using $d^{j}_{x,y}$ as a soft weight. The proposed label correction compensation is

5 $$\begin{aligned} \mathbf{Q} ^{j}=\mathbf{D} ^{j} \odot \widetilde{\mathbf{S }}^{j} + \left( \mathbf{1} -\mathbf{D} ^{j}\right) \odot \mathbf{S} ^{j}, \end{aligned}$$

where $\odot$ denotes the element-wise multiplication, $\mathbf{D} ^{j}$ is composed of $d^{j}_{x,y}$ reflecting the pixel-level confidence of the segmentation results. We take the segmentation results from $\mathbf{S} ^{j}$ where the prediction confidence is high. Otherwise, we use the historical best prediction $\widetilde{\mathbf{S }}^{j}$ that is more stable when the confidence is low.

### Implementation details

In this work, we choose the classical binary cross entropy loss as *E* in Eq. (4) and use U-Net [2] as network *G*. To efficiently store the $\widetilde{S}^{j}_{x,y}$ and $\overline{S}^{j}_{x,y}$ on each pixel, we employ a dict structure, named as *Memory Bank* in Fig. 1, to record the $\widetilde{S}^{j}_{x,y}$ and $\overline{S}^{j}_{x,y}$ according to the image index and the (*x*, *y*) coordinates. During training, for each image, we perform horizontal flipping, vertical flipping, and both of them respectively, to construct three augmented images. The memory bank will first reverse the augmentation operations of the augmented images on their prediction label maps, then calculate and record the $\widetilde{S}^{j}_{x,y}$ and $\overline{S}^{j}_{x,y}$. We use the Adam [38] optimizer with learning rate $7 \times {10}^{-3}$. Following the setting in [12], we also use stochastic weight averaging method [16] to train the network.

We run our method for 100 epochs in total, the first 50 epochs as initialization following with 5 cycles, each containing 10 epochs. We apply SGDR [39] learning rate scheduler to adjust the learning rate dynamically. The learning rate scheduler begins to work at epoch 40 and with 10 as cyclical epoch number.

## Results

### Setup

We evaluate two tasks in the experiments: 1) We train the network on the training dataset using only the noisy label (as the initial label) and evaluate its segmentation results on the testing dataset with the correct labels. 2) We evaluate the noisy label correction on the training dataset using the correct labels.

*Datasets* We evaluate our methods on 3 public benchmarks.

- *DRIVE* [17] contains 40 retina images with size $565\times 584$, 20 images in training set and 20 images in testing set. Each image in the training set has the label map annotated by an expert (taken as the golden standard, i.e., correct label). Besides the correct label maps, each image in the testing set has a label map annotated by another annotator (taken as the noisy label). To satisfy our task in this work, we exchange the data in the training set and testing set and denote the new dataset as *DRIVE(R)*.
- *STARE (VK)* [21] contains 20 images with the resolution of $605\times 700$: first 10 in the training set and the other 10 in the testing set.
- *CHASE* [22] contains 28 retina images with the resolution of $999\times 960$: first 14 for training and the other 14 for testing. In these two datasets, each image has two label maps annotated by two annotators. According to the official description, the label maps from one expert are taken as the golden standard.

*Comparison methods* We include following 3 methods for comparison.

- *U-Net*: We select a famous network architecture for image segmentation namely U-Net [2] as the baseline, which maintains the same backbone network and training settings as ours.
- *Cas *[11]: A method for chest X-ray image segmentation task, which also provides the noisy label correction results.
- *SF *[12]: A state-of-the-art method for noisy label based human parsing and label correction.

**Fig. 3:**
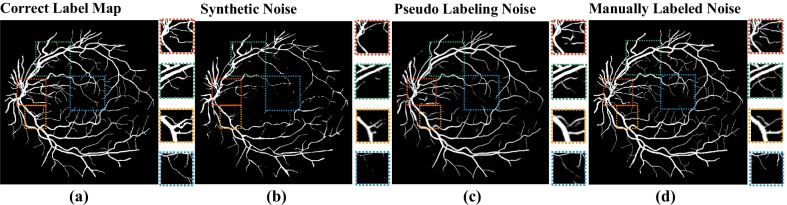
Noisy label maps from different pollution source:** a** correct label map without noise,** b** label map with synthetic noise,** c** label map with pseudo labeling noise,** d** label map with manually labeled noise

*Pollution sources* We use three types of pollution sources, i.e., (1) synthetic noisy label maps, (2) label maps generated by pseudo labeling, and (3) manually labeled noisy label maps to evaluate the label correction performance of our method and the comparison methods. Examples of them are shown in Fig. 3b–d respectively. The original label map is shown in Fig. 3a for comparison.

- We apply the method in [9] to generate the synthetic noisy label maps. We approximate the contour of the retina vessel using the combination of line segments using the tool OpenCV. This could result in pixel label deletion, shifting, and inaccurate contours, which is to simulate the noises in roughly annotating retina vessel images. We control the parameter of approximation accuracy and generate noisy label maps with three aggravated pollution levels, named as $\mathrm {LV}$-1, $\mathrm {LV}$-2, and $\mathrm {LV}$-3.
- For unlabeled segmentation datasets in practical scenes, pseudo label maps generated by models trained on other similar labeled datasets are often used as low-cost noisy supervision. So we also collect pseudo label maps of DRIVE (R) and STARE (VK) datasets generated by existing published work [40] as shown in Fig. 3c.
- The manually labeled noisy label maps are from the manual label maps (other than the golden standard) provided by the above three datasets.

All of the noisy labels for the three datasets used in this work are submitted as described in the section of Additional Files, Additional file 1.

### Results of label correction

We first evaluate the noisy label correction performance in Table 1. Specifically, we compare the original noisy label with the corrected label generated by different methods using standard segmentation metrics, including the $F_1$ score and area under the precision-recall curve (PR score). As shown in Table 1, the results in ‘Baseline’ denote the accuracy of the labels under different polluted sources.

**Table 1:** Comparative results of prediction on the testing set (%)

Dataset		DRIVE(R)		STARE(VK)		CHASE	
Group	Method	F1	PR	F1	PR	F1	PR
$\mathrm {LV}$-1	Baseline	73.2	76.0	75.7	77.7	81.9	82.8
	Cas	76.1	83.5	72.6	78.8	79.9	87.8
	SF	78.4	87.8	78.0	87.1	85.5	93.9
	Ours	**79.6**	**88.6**	**79.9**	**88.4**	**85.9**	**94.1**
$\mathrm {LV}$-2	Baseline	70.2	72.8	72.3	74.2	77.2	78.3
	Cas	75.1	82.0	72.9	79.8	77.4	85.4
	SF	75.7	84.5	77.3	86.0	83.6	92.2
	Ours	**77.6**	**87.1**	**78.9**	**87.3**	**84.3**	**92.6**
$\mathrm {LV}$-3	Baseline	67.2	69.6	69.4	71.3	73.8	74.9
	Cas	75.1	82.3	71.1	76.9	77.7	85.4
	SF	73.2	82.4	75.0	83.5	82.4	91.0
	Ours	**77.0**	**86.4**	**77.6**	**86.4**	**83.1**	**91.4**
Pseudo	Baseline	79.3	87.2	76.0	83.9	/	/
	Cas	75.7	82.8	74.0	81.3	/	/
	SF	80.0	88.1	**76.7**	**84.8**	/	/
	Ours	**80.3**	**88.5**	76.5	84.2	/	/
Manual	Baseline	78.9	79.9	72.2	76.0	76.3	78.2
	Cas	78.1	85.8	73.4	79.8	78.8	81.8
	SF	82.8	91.2	76.7	84.1	82.7	90.4
	Ours	**83.0**	**91.3**	**77.8**	**84.3**	**83.3**	**91.1**

The values with bold denote the best performance in each group

The proposed method consistently outperforms all the other methods in all the benchmarks for the synthetic noises, especially in LV-3 groups. It corrects the original noisy label maps to a more accurate label map by 4.0–$9.8\%$ on $F_1$ and 10.7–$16.8\%$ on PR on three testing datasets.

For the pseudo labeling noise, the proposed method could also improve the quality of the pseudo label map by a small margin.

For the manually labeled noise, the proposed method shows better accuracy than other methods, especially on the STARE (VK) dataset, where it outperforms the SF method and Cas method by $1.1\%$ and $4.4\%$ on $F_1$ score. Compared to the original noisy label maps, it obtains the improvement of 4.1–$7.0\%$ on $F_1$ score on three datasets.

### Testing performance boost of segmentation

We further evaluate the segmentation performance boost of our method and the comparison methods on the testing set using the same initial noisy labels for training. The results are shown in Table 2 and the ‘Baseline’ here denotes the *U-Net* described in the Setup Sect. . We can see that the segmentation performance improvement of the proposed method is also superior compared with others in most experiments. Notably, when the level of synthetic noise is serious, e.g., $\mathrm {LV}$-3, the proposed method could also boost the segmentation performance of the network while other two methods fail in some cases, e.g., those on DRIVE (R) and STARE (VK).

**Table 2:** Comparative results of prediction on testing set.(%)

Dataset		DRIVE(R)		STARE(VK)		CHASE	
Group	Method	F1	PR	F1	PR	F1	PR
$\mathrm {LV}$-1	U-Net	73.9	82.9	79.6	87.4	77.4	**85.8**
	Cas	73.6	81.9	75.1	82.1	73.4	80.7
	SF	76.3	84.9	80.4	88.2	76.5	84.7
	Ours	**76.8**	**85.5**	**81.3**	**88.9**	**77.5**	85.3
$\mathrm {LV}$-2	U-Net	74.5	81.3	79.1	87.3	70.7	78.4
	Cas	73.4	82.0	74.4	82.0	73.1	80.5
	SF	73.7	82.4	80.0	87.6	75.3	82.9
	Ours	**75.9**	**84.8**	**80.8**	**88.1**	**75.9**	**83.4**
$\mathrm {LV}$-3	U-Net	72.3	81.4	78.6	86.4	70.1	77.0
	Cas	72.2	80.2	69.7	76.1	69.9	76.6
	SF	72.0	80.3	76.9	85.3	74.4	81.5
	Ours	**75.7**	**84.3**	**79.2**	**87.0**	**74.8**	**82.8**
Pseudo	U-Net	78.1	86.5	80.1	88.3	/	/
	Cas	74.0	82.2	75.8	83.8	/	/
	SF	78.5	87.0	80.3	88.5	/	/
	Ours	**78.7**	**87.2**	**80.6**	**88.6**	/	/
Manual	U-Net	80.2	88.7	80.0	87.9	77.7	85.0
	Cas	76.5	85.1	73.8	81.1	73.9	81.0
	SF	80.9	89.5	81.3	88.7	79.4	87.2
	Ours	**81.3**	**89.8**	**82.0**	**89.1**	**80.0**	**88.0**

The values with bold denote the best performance in each group

### Cross-datasets validation

To evaluate the generalization ability of the proposed method and other compared methods, we use the models trained on the DRIVE(R) dataset to predict segmentation label maps on the test set of the STARE dataset and the other way round for cross-datasets validation. The results are shown in Table 3.

**Table 3:** Cross validation on DRIVE(R) and STARE datasets.(%)

Dataset		DRIVE(R)		STARE(VK)	
Group	Method	F1	PR	F1	PR
$\mathrm {LV}$-1	U-Net	68.5	75.6	70.3	78.6
	Cas	36.0	36.0	70.8	74.8
	SF	65.0	71.2	72.2	80.2
	Ours	**73.9**	**81.2**	**74.5**	**82.3**
$\mathrm {LV}$-2	U-Net	68.7	74.6	70.1	77.3
	Cas	47.0	48.5	72.4	79.8
	SF	66.7	72.6	70.9	78.3
	Ours	**71.8**	**79.0**	**73.0**	**79.9**
$\mathrm {LV}$-3	U-Net	49.0	46.1	67.0	74.7
	Cas	50.0	54.5	**70.5**	77.9
	SF	67.8	73.5	68.7	76.4
	Ours	**70.2**	**76.4**	**70.5**	**78.4**
Pseudo	U-Net	57.4	62.7	72.2	78.1
	Cas	49.6	52.2	76.9	83.5
	SF	70.9	76.9	74.7	81.1
	Ours	**76.3**	**84.4**	**77.8**	**85.7**
Manual	U-Net	55.3	59.7	70.6	77.3
	Cas	61.9	68.3	75.5	82.0
	SF	65.9	71.3	74.9	82.7
	Ours	**71.4**	**78.6**	**77.1**	**83.6**

The values with bold denote the best performance in each group

From Table 3, we can see a performance decrease of all the methods on both of the datasets, especially the STARE dataset. This is because the images and annotations have a domain gap between these two datasets with different capturing devices and different human annotators. However, the proposed method still achieves considerable high performance in the cross-datasets validation and outperforms other compared methods in all the metrics across different noise settings. Even in the high synthetic noise groups like LV-3, the proposed method still gets the F1 score over 70.0 on both the DRIVE(R) and STARE datasets. The experimental results support that the proposed method has a good generalization ability under different levels of label noises.

### Qualitative study

**Fig. 4:**
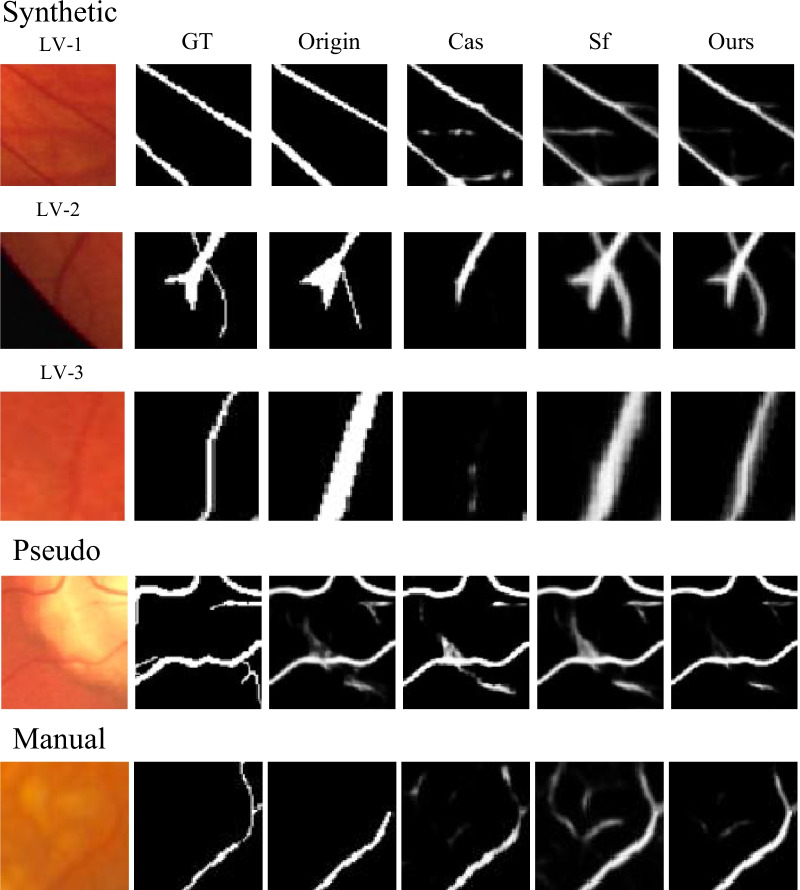
Visual results of the corrected label maps on training set of the comparison methods and the proposed method

We show the cases of corrected label maps of different types of noise in Fig. 4. We could see that the proposed method tends to correct the noisy labels carefully while preserving the correct labels unchanged. The compared methods either couldn’t correct the noise or failed to preserve the correct labels unchanged, such as the cases shown in lines 1, 4, and 5 in Fig. 4. Besides, the proposed method could generate more accurate boundary and thickness of the vessels than the compared methods, such as the cases shown in lines 2 and 3 in Fig. 4. This could be explained by the proposed method considering both the noisy labels in training and noisy predictions in label correction. Thus, for example, if the labels of the vessel are thicker than its correct labels at the boundary, the network in the proposed framework will not be directly influenced by the noisy labels, which otherwise may result in thicker vessel predictions. The full corrected label maps are shown in Fig. 6.

### Training-testing curve

**Fig. 5:**
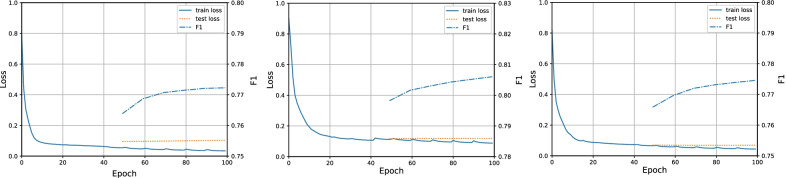
Change curve of train loss, test loss, and the label correction performance with the training epoch increasing

**Fig. 6:**
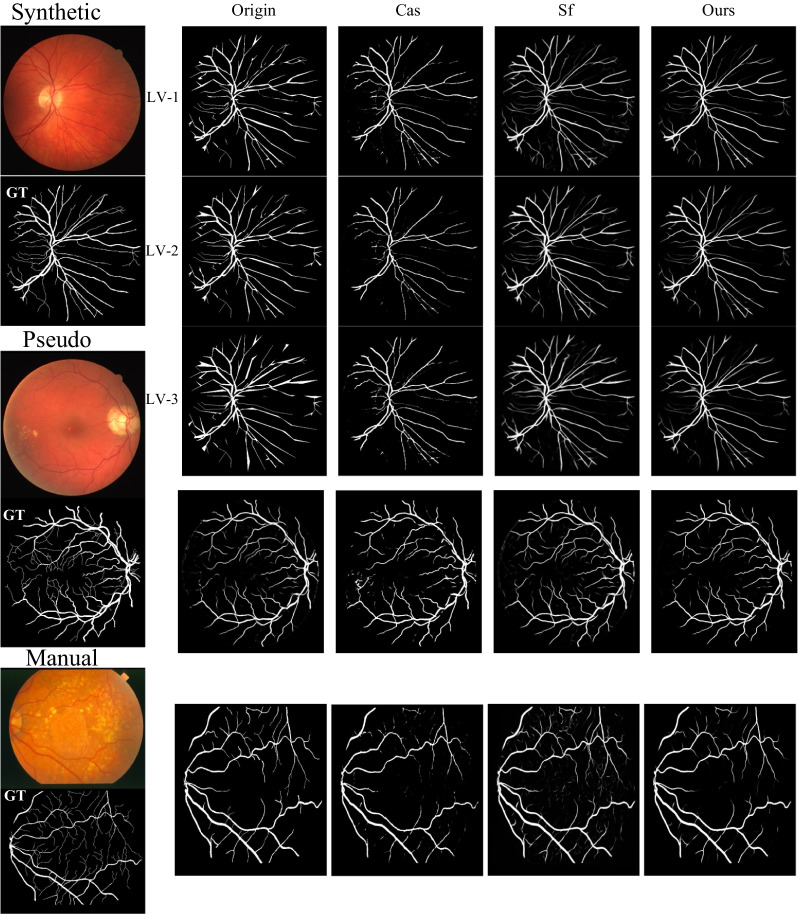
Full visual results of the corrected label maps on training set of the comparison methods and the proposed method

We further show the training-testing loss curve and the F1 curve of label map correction in Fig. 5 to understand the training procedure better. As mentioned in the Implement Details Sect. , we train the whole framework for 100 epochs and start the first cycle of label correction and testing at epoch 50. From Fig. 5 we could see that the testing loss curves continuously decrease during multiple training cycles. While the training loss curves are almost constantly reducing as well, except that at the beginning epoch of each cycle, it will get a small peak. This is because the label map is corrected at the end of each cycle, and the SGDR learning rate scheduler will warm up at the beginning of each cycle. The two curves support that the proposed method is not over-fitted to the evaluated datasets. Besides, the F1 curve of label map correction is also continuously increasing. The progress of network training and label map correction will promote each other and further boost the performance of the whole framework (Fig. 6).

### Ablation study

In this section, we apply the ablation study to the label map correction task. We consider the following variations of the proposed method.

- **w/o TML**: We remove the proposed temporal memory loss, i.e., only use $E(\mathbf{S} ^e,\mathbf{L} ^{j})$ as loss function in Eq. (4).
- **w** $\mathbf{S} ^j$ : Replacing $\mathbf{Q} ^{j}$ with $\mathbf{S} ^{j}$ in Eq. (5).
- **w** $\widetilde{\mathbf{S }}^j$ : Replacing $\mathbf{Q} ^{j}$ with $\widetilde{\mathbf{S }}^j$ in Eq. (5).

The results are shown in Table 4. Without using the proposed TML in training will decrease the performance of the proposed framework among all the benchmarks by the range of 0.3–1.6$\%$ on $F_1$ score and 0.2–$2.4\%$ on PR score. Notably, when the degree of synthetic noise increases, we can see a larger performance decrease margin if we remove TML. For example, on the DRIVE (R) dataset with $\mathrm {LV}$-1 synthetic noise, removing TML brings a $1.1\%$ decrease on PR score. While with $\mathrm {LV}$-3 synthetic noise, the corresponding performance decreases by $2.4\%$. Using $\mathbf{S} ^j$ as $\mathbf{Q} ^{j}$ in Eq. (5) will also consistently decrease the performance among all the benchmarks. It will downgrade the performance by 0.5–$2.0\%$ on $F_1$ score and 0.4–$1.2\%$ on PR score. Using the $\widetilde{\mathbf{S }}^j$ to replace $\mathbf{Q} ^{j}$ in Eq. (5) is slightly better than the proposed $\mathbf{Q} ^{j}$ in some cases, especially in low-level synthetic noises, such as LV-1 and LV-2 on CHASE. However in most of the benchmarks the proposed $\mathbf{Q} ^{j}$ is superior to the $\widetilde{\mathbf{S }}^j$ in label correction.

**Table 4:** Ablation study of label correction task.(%)

Dataset		DRIVE(R)		STARE(VK)		CHASE	
Group	Method	F1	PR	F1	PR	F1	PR
$\mathrm {LV}$-1	w/o TML	78.5	87.5	79.0	87.4	85.5	93.9
	w $\mathbf{S} ^j$	79.3	87.7	78.9	87.2	85.5	93.8
	w $\widetilde{\mathbf{S }}^j$	**79.8**	88.1	79.5	87.7	**86.2**	**94.2**
	Ours	79.6	**88.6**	**79.9**	**88.4**	85.9	94.1
$\mathrm {LV}$-2	w/o TML	76.4	85.9	78.0	86.5	83.6	92.2
	w $\mathbf{S} ^j$	76.7	85.7	78.3	86.7	83.8	92.4
	w $\widetilde{\mathbf{S }}^j$	77.1	84.7	77.9	87.0	**84.5**	**92.6**
	Ours	**77.6**	**87.1**	**78.9**	**87.3**	84.3	**92.6**
$\mathrm {LV}$-3	w/o TML	75.6	84.0	76.9	85.0	82.4	91.0
	w $\mathbf{S} ^j$	74.4	82.9	76.0	84.4	81.3	89.0
	w $\widetilde{\mathbf{S }}^j$	**77.1**	84.7	**77.9**	**87.0**	**84.5**	**92.6**
	Ours	77.0	**86.4**	77.6	86.4	83.1	91.4
Pseudo	w/o TML	79.9	88.0	76.2	83.9	/	/
	w $\mathbf{S} ^j$	80.0	88.0	76.3	84.0	/	/
	w $\widetilde{\mathbf{S }}^j$	80.1	87.9	**76.7**	**84.8**	/	/
	Ours	**80.3**	**88.5**	76.5	84.2	/	/
Manual	w/o TML	82.6	90.5	76.2	83.4	82.7	90.6
	w $\mathbf{S} ^j$	82.5	90.9	75.8	83.1	82.5	90.4
	w $\widetilde{\mathbf{S }}^j$	82.6	90.8	77.6	**84.7**	82.8	90.1
	Ours	**83.0**	**91.3**	**77.8**	84.3	**83.3**	**91.1**

The values with bold denote the best performance in each group

## Conclusion

In this paper, we developed a new noise-tolerant method to train the segmentation network on noisy label maps and improve the quality of the initial label maps in the meantime. More specifically, we considered the temporal-integrated segmentation prediction during network training at different epochs and used it for self-supervised network training and noisy label correction. Experiments on the DRIVE, STARE, and CHASE-DB1 datasets verified that the proposed method could achieve better retinal image segmentation performance than many existing methods by simultaneously correcting the noise in the initial label map.

## Supplementary Information

**Additional file 1**. Generated noisy label maps.

## Data Availability

The datasets generated and analyzed during the current study are available on the following websites: https://drive.grand-challenge.org/, https://cecas.clemson.edu/ ahoover/stare/, and https://blogs.kingston.ac.uk/retinal/chasedb1/ respectively. The generated noisy label maps are included in the Additional file 1.

## Abbreviations

LNL: Learning from noisy labels
STM: Segmentation training module
LCM: Label correction module
TML: Temporal memory loss
PR: Area under the precision-recall curve

## References

[1] Zhou Z, Siddiquee MMR, Tajbakhsh N, Liang J. Unet++: a nested u-net architecture for medical image segmentation. In: Deep learning in medical image analysis and multimodal learning for clinical decision support. Cham: Springer; 2018. p. 3– 11. 10.1007/978-3-030-00889-5_1 PMC7329239 32613207

[2] Ronneberger O, Fischer P, Brox T. U-net: convolutional networks for biomedical image segmentation. In: International conference on medical image computing and computer-assisted intervention; 2015. p. 234–41.

[3] Isensee F, Jaeger PF, Kohl SA, Petersen J, Maier-Hein KH (2021). nnU-Net: a self-configuring method for deep learning-based biomedical image segmentation. Nat Methods.

[4] Jiang L, Zhou Z, Leung T, Li L-J, Fei-Fei L. Mentornet: learning data-driven curriculum for very deep neural networks on corrupted labels. In: International conference on machine learning; 2018. p. 2304–13.

[5] Tanaka D, Ikami D, Yamasaki T, Aizawa K. Joint optimization framework for learning with noisy labels. In: IEEE conference on computer vision and pattern recognition; 2018.

[6] Menon A, Van Rooyen B, Ong CS, Williamson B. Learning from corrupted binary labels via class-probability estimation. In: International conference on machine learning; 2015.

[7] Han B, Yao Q, Yu X, Niu G, Xu M, Hu W, Tsang I, Sugiyama M. Co-teaching: robust training of deep neural networks with extremely noisy labels. In: Advances in neural information processing systems; 2018. p. 8527–37.

[8] Yi K, Wu J. Probabilistic end-to-end noise correction for learning with noisy labels. In: IEEE/CVF conference on computer vision and pattern recognition; 2019.

[9] Mirikharaji Z, Yan Y, Hamarneh G. Learning to segment skin lesions from noisy annotations. In: Domain adaptation and representation transfer and medical image learning with less labels and imperfect data; 2019. p. 207–15.

[10] Nie D, Gao Y, Wang L, Shen D. Asdnet: attention based semi-supervised deep networks for medical image segmentation. In: International conference on medical image computing and computer-assisted intervention; 2018. p. 370–8.

[11] Xue C, Deng Q, Li X, Dou Q, Heng P-A. Cascaded robust learning at imperfect labels for chest x-ray segmentation. In: International conference on medical image computing and computer-assisted intervention; 2020. p. 579–88.

[12] Li P, Xu Y, Wei Y, Yang Y. Self-correction for human parsing. IEEE Trans Pattern Anal Mach Intell. 2020. 10.1109/TPAMI.2020.3048039 33373297

[13] Liu J, Li R, Sun C. Co-correcting: noise-tolerant medical image classification via mutual label correction. IEEE Trans. Med. Imaging. 2021. 10.1109/TMI.2021.3091178 34152981

[14] Zhang T, Yu L, Hu N, Lv S, Gu S. Robust medical image segmentation from non-expert annotations with tri-network. In: International Conference on medical image computing and computer-assisted intervention; 2020.

[15] Athiwaratkun B, Finzi M, Izmailov P, Wilson AG. There are many consistent explanations of unlabeled data: why you should average. In: International conference on learning representations; 2019.

[16] Izmailov P, Podoprikhin D, Garipov T, Vetrov D, Wilson AG. Averaging weights leads to wider optima and better generalization; 2018.

[17] Staal J, Abràmoff MD, Niemeijer M, Viergever MA, Van Ginneken B (2004). Ridge-based vessel segmentation in color images of the retina. IEEE Trans Med Imaging.

[18] Soares JV, Leandro JJ, Cesar RM, Jelinek HF, Cree MJ (2006). Retinal vessel segmentation using the 2-D Gabor wavelet and supervised classification. IEEE Trans Med Imaging.

[19] Jin Q, Meng Z, Pham TD, Chen Q, Wei L, Su R (2019). Dunet: a deformable network for retinal vessel segmentation. Knowl-Based Syst.

[20] Srinidhi CL, Aparna P, Rajan J (2017). Recent advancements in retinal vessel segmentation. J Med Syst.

[21] Hoover A, Kouznetsova V, Goldbaum M (2000). Locating blood vessels in retinal images by piecewise threshold probing of a matched filter response. IEEE Trans Med Imaging.

[22] Owen CG, Rudnicka AR, Mullen R, Barman SA, Monekosso D, Whincup PH, Ng J, Paterson C (2009). Measuring retinal vessel tortuosity in 10-year-old children: validation of the computer-assisted image analysis of the retina (CAIAR) program. Investig Ophthalmol Vis Sci.

[23] Arpit D, Jastrzębski S, Ballas N, Krueger D, Bengio E, Kanwal MS, Maharaj T, Fischer A, Courville A, Bengio Y, et al. A closer look at memorization in deep networks. In: International conference on machine learning; 2017. p. 233–42.

[24] Tajbakhsh N, Jeyaseelan L, Li Q, Chiang JN, Wu Z, Ding X (2020). Embracing imperfect datasets: a review of deep learning solutions for medical image segmentation. Med Image Anal.

[25] Li W, Wang L, Li W, Agustsson E, Van Gool L. Webvision database: visual learning and understanding from web data; 2017. arXiv:1708.02862.

[26] Malach E, Shalev-Shwartz S. Decoupling“ when to update” from “ how to update”; 2017. arXiv:1706.02613.

[27] Chang H-S, Learned-Miller E, McCallum A. Active bias: training more accurate neural networks by emphasizing high variance samples; 2017. arXiv:1704.07433.

[28] Gao J, Jagadish H, Ooi BC. Active sampler: light-weight accelerator for complex data analytics at scale; 2015. arXiv:1512.03880.

[29] Kapil A, Meier A, Zuraw A, Steele KE, Rebelatto MC, Schmidt G, Brieu N (2018). Deep semi supervised generative learning for automated tumor proportion scoring on NSCLC tissue needle biopsies. Sci Rep.

[30] Liu Y, Deng G, Zeng X, Wu S, Yu Z, Wong H-S. Regularizing discriminative capability of cgans for semi-supervised generative learning. In: Proceedings of the IEEE/CVF conference on computer vision and pattern recognition; 2020. p. 5720–9.

[31] Inoue N, Goto K. Semi-supervised contrastive learning with generalized contrastive loss and its application to speaker recognition. In: 2020 Asia-Pacific signal and information processing association annual summit and conference (APSIPA ASC); 2020. p. 1641–6.

[32] Grandvalet Y, Bengio Y, et al. Semi-supervised learning by entropy minimization. In: CAP; 2005. p. 281– 296.

[33] Tarvainen A, Valpola H. Weight-averaged, consistency targets improve semi-supervised deep learning results. CoRR, 1780; vol. abs/1703 (2017).

[34] Perone CS, Cohen-Adad J. Deep semi-supervised segmentation with weight-averaged consistency targets. In: Deep learning in medical image analysis and multimodal learning for clinical decision support; 2018. p. 12–9.

[35] Xie Q, Luong M-T, Hovy E, Le QV. Self-training with noisy student improves imagenet classification. In: Proceedings of the IEEE/CVF conference on computer vision and pattern recognition; 2020. p. 10687–98.

[36] Zhang P, Zhang B, Zhang T, Chen D, Wen F. Robust mutual learning for semi-supervised semantic segmentation. 2021.

[37] Ke Z, Di Qiu KL, Yan Q, Lau RW. Guided collaborative training for pixel-wise semi-supervised learning. In: European conference on computer vision, vol. 2; 2020. p. 6.

[38] Kingma DP, Ba J. Adam: a method for stochastic optimization. 2014.

[39] Loshchilov I, Hutter F. Sgdr: stochastic gradient descent with warm restarts. 2016.

[40] Yan Z, Yang X, Cheng K-T (2018). Joint segment-level and pixel-wise losses for deep learning based retinal vessel segmentation. IEEE Trans Biomed Eng.

