# Supplementary figures and images for: Self-relabeling for noise-tolerant retina vessel segmentation through label reliability estimation (part 2 of 2)
Source: BMC Med Imaging. 2022 Jan 12;22:8. doi: 10.1186/s12880-021-00732-y (PMC8753937; doi:10.1186/s12880-021-00732-y)

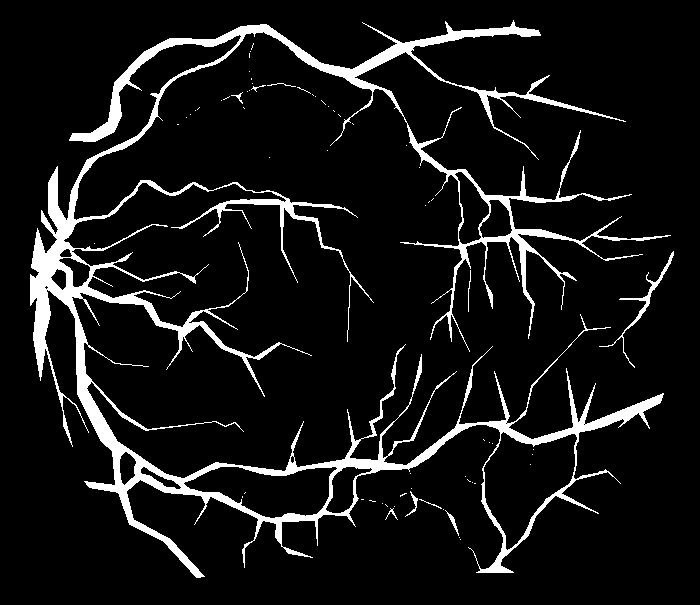

Supplement: Supplementary file 1 — Additional file 1. Generated noisy label maps. [file 12880_2021_732_MOESM1_ESM.zip › Noisy_label_maps/STARE/LV1/im0001.png]

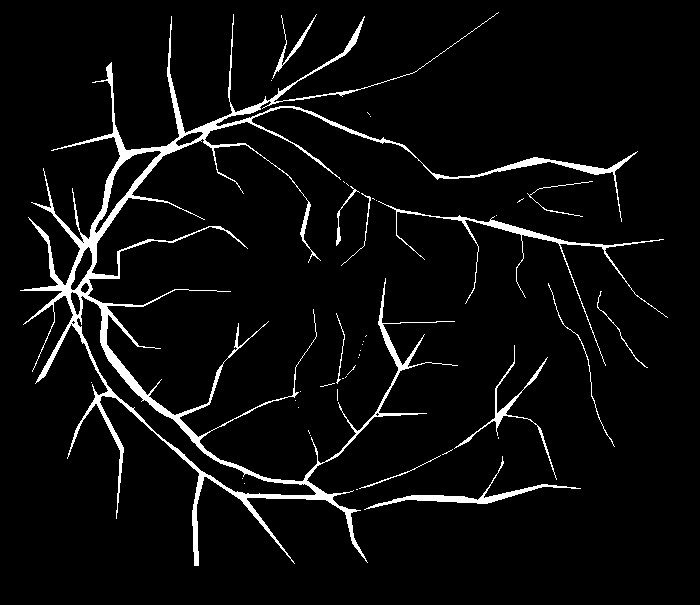

Supplement: Supplementary file 1 — Additional file 1. Generated noisy label maps. [file 12880_2021_732_MOESM1_ESM.zip › Noisy_label_maps/STARE/LV1/im0002.png]

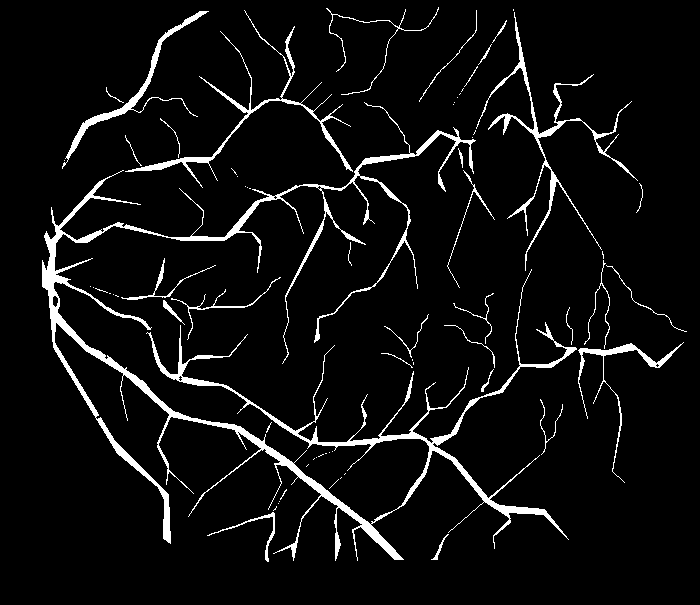

Supplement: Supplementary file 1 — Additional file 1. Generated noisy label maps. [file 12880_2021_732_MOESM1_ESM.zip › Noisy_label_maps/STARE/LV1/im0003.png]

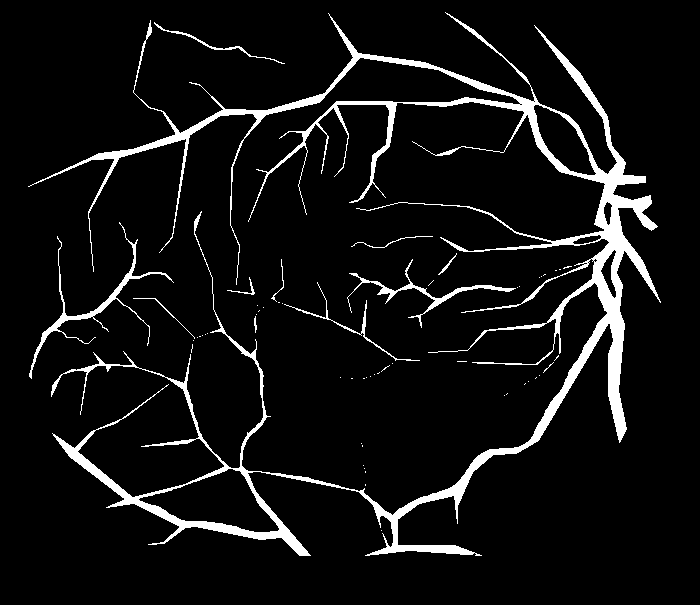

Supplement: Supplementary file 1 — Additional file 1. Generated noisy label maps. [file 12880_2021_732_MOESM1_ESM.zip › Noisy_label_maps/STARE/LV1/im0004.png]

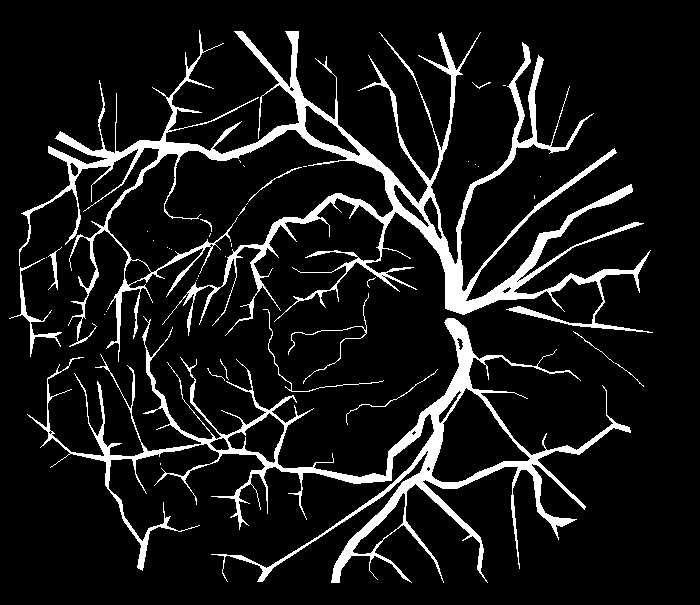

Supplement: Supplementary file 1 — Additional file 1. Generated noisy label maps. [file 12880_2021_732_MOESM1_ESM.zip › Noisy_label_maps/STARE/LV1/im0005.png]

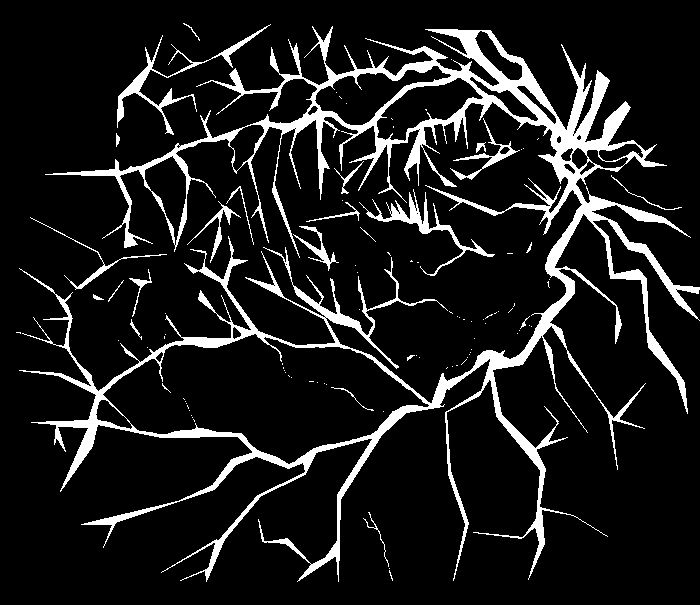

Supplement: Supplementary file 1 — Additional file 1. Generated noisy label maps. [file 12880_2021_732_MOESM1_ESM.zip › Noisy_label_maps/STARE/LV1/im0044.png]

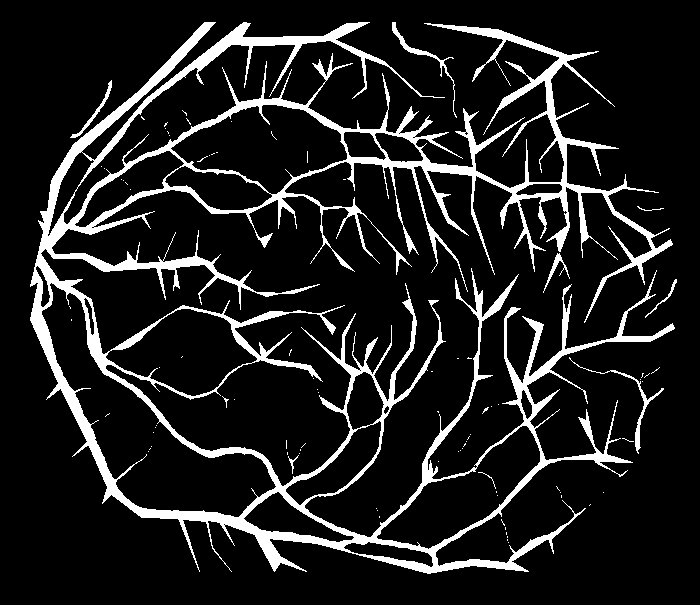

Supplement: Supplementary file 1 — Additional file 1. Generated noisy label maps. [file 12880_2021_732_MOESM1_ESM.zip › Noisy_label_maps/STARE/LV1/im0077.png]

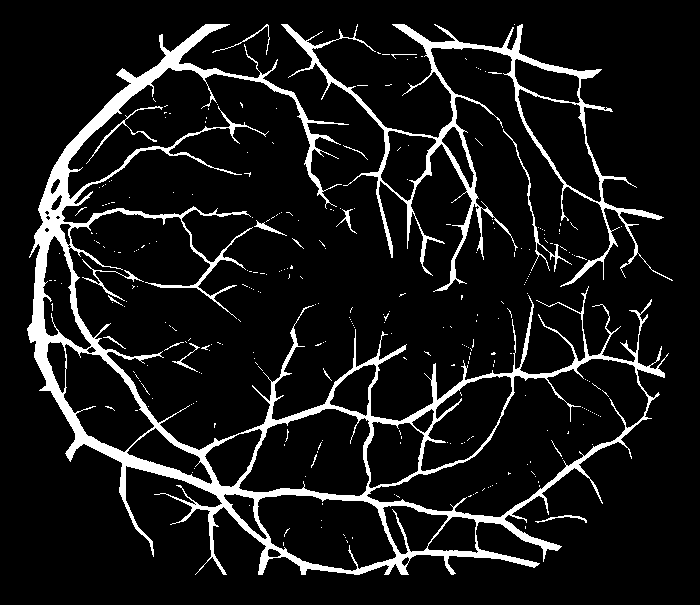

Supplement: Supplementary file 1 — Additional file 1. Generated noisy label maps. [file 12880_2021_732_MOESM1_ESM.zip › Noisy_label_maps/STARE/LV1/im0081.png]

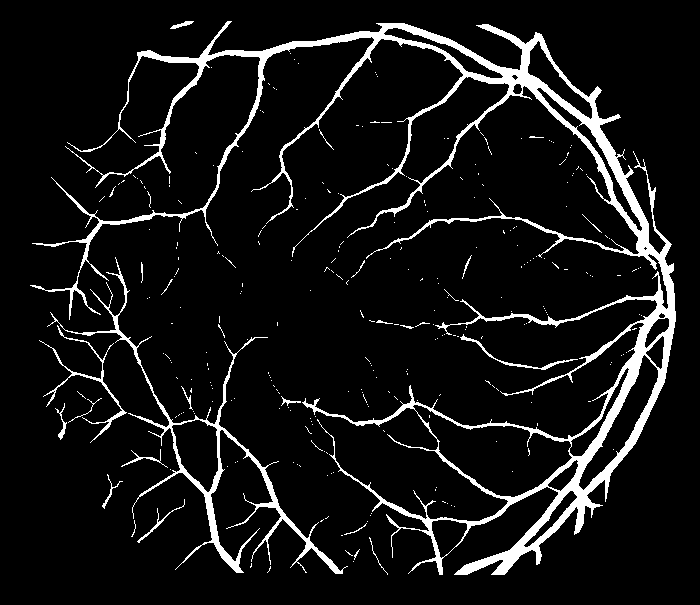

Supplement: Supplementary file 1 — Additional file 1. Generated noisy label maps. [file 12880_2021_732_MOESM1_ESM.zip › Noisy_label_maps/STARE/LV1/im0082.png]

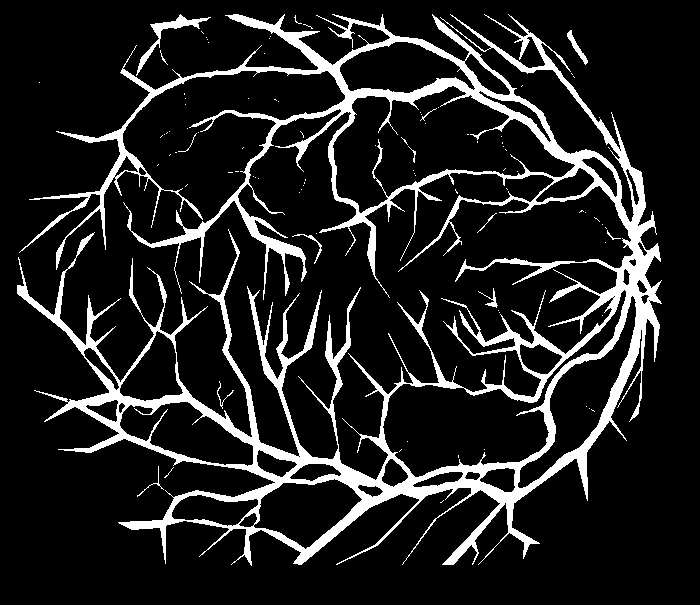

Supplement: Supplementary file 1 — Additional file 1. Generated noisy label maps. [file 12880_2021_732_MOESM1_ESM.zip › Noisy_label_maps/STARE/LV1/im0139.png]

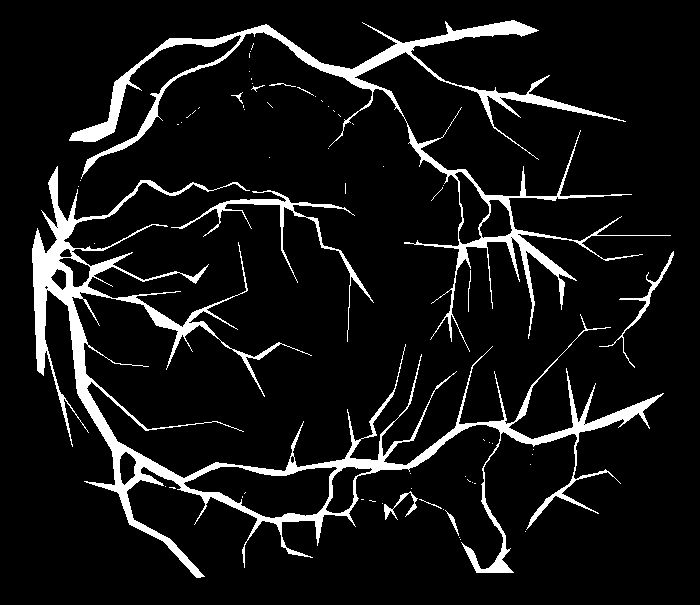

Supplement: Supplementary file 1 — Additional file 1. Generated noisy label maps. [file 12880_2021_732_MOESM1_ESM.zip › Noisy_label_maps/STARE/LV3/im0001.png]

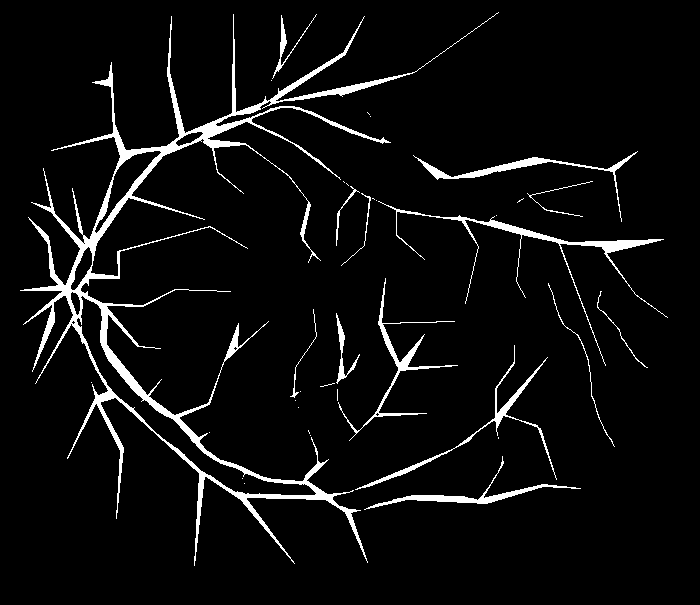

Supplement: Supplementary file 1 — Additional file 1. Generated noisy label maps. [file 12880_2021_732_MOESM1_ESM.zip › Noisy_label_maps/STARE/LV3/im0002.png]

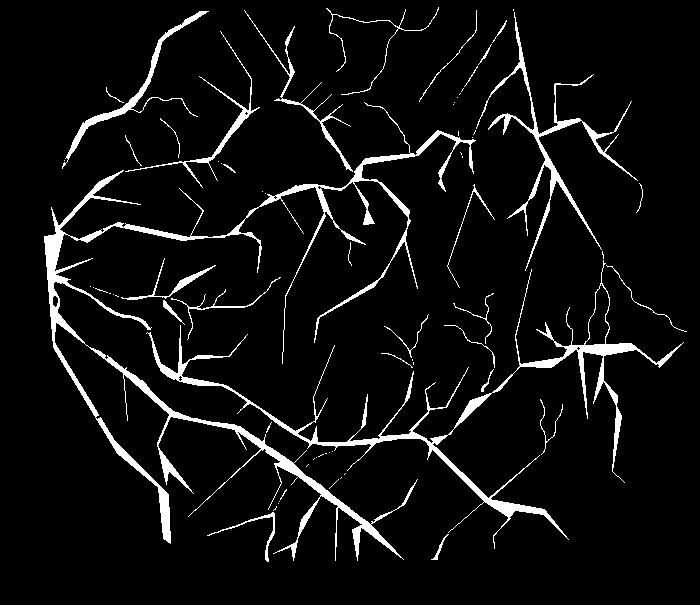

Supplement: Supplementary file 1 — Additional file 1. Generated noisy label maps. [file 12880_2021_732_MOESM1_ESM.zip › Noisy_label_maps/STARE/LV3/im0003.png]

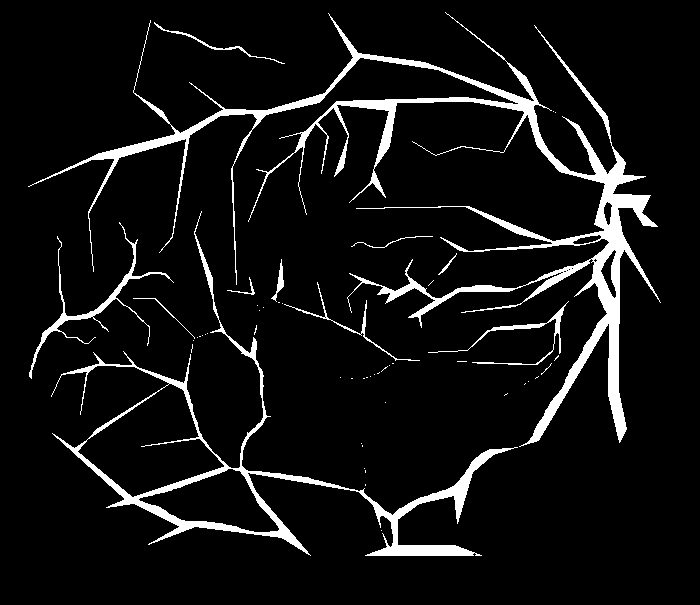

Supplement: Supplementary file 1 — Additional file 1. Generated noisy label maps. [file 12880_2021_732_MOESM1_ESM.zip › Noisy_label_maps/STARE/LV3/im0004.png]

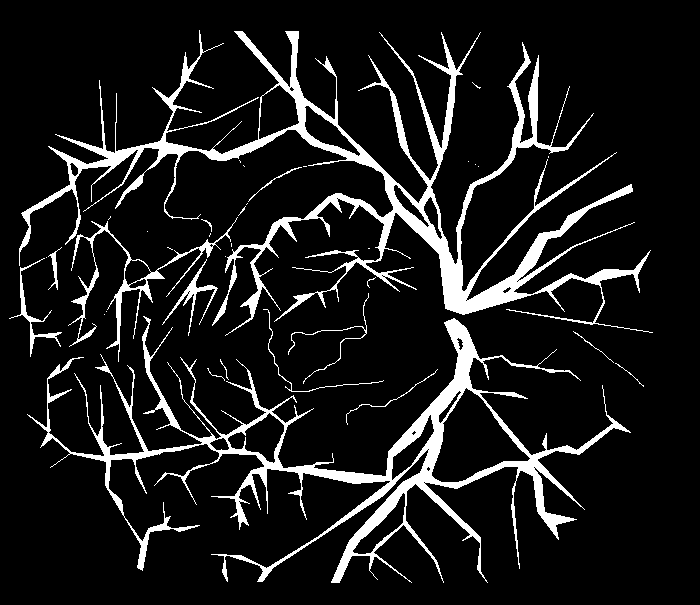

Supplement: Supplementary file 1 — Additional file 1. Generated noisy label maps. [file 12880_2021_732_MOESM1_ESM.zip › Noisy_label_maps/STARE/LV3/im0005.png]

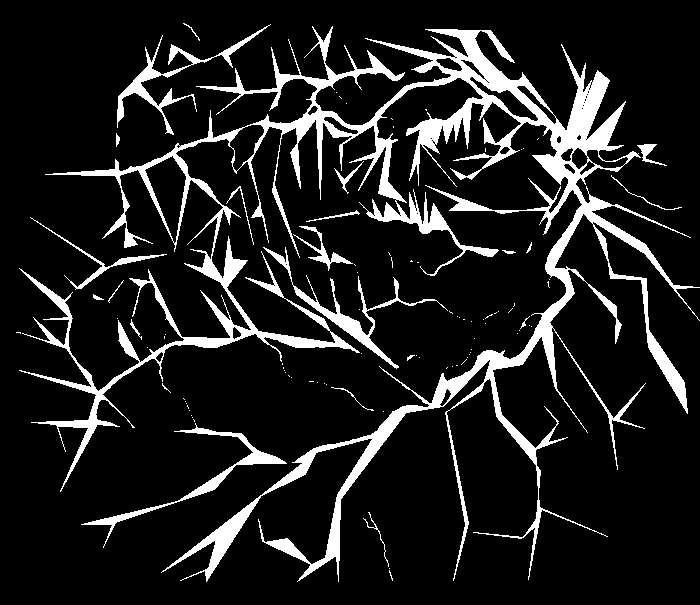

Supplement: Supplementary file 1 — Additional file 1. Generated noisy label maps. [file 12880_2021_732_MOESM1_ESM.zip › Noisy_label_maps/STARE/LV3/im0044.png]

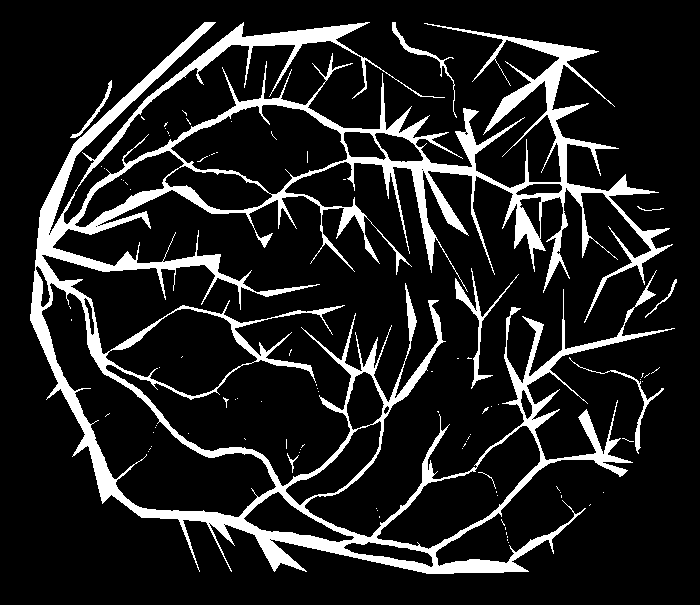

Supplement: Supplementary file 1 — Additional file 1. Generated noisy label maps. [file 12880_2021_732_MOESM1_ESM.zip › Noisy_label_maps/STARE/LV3/im0077.png]

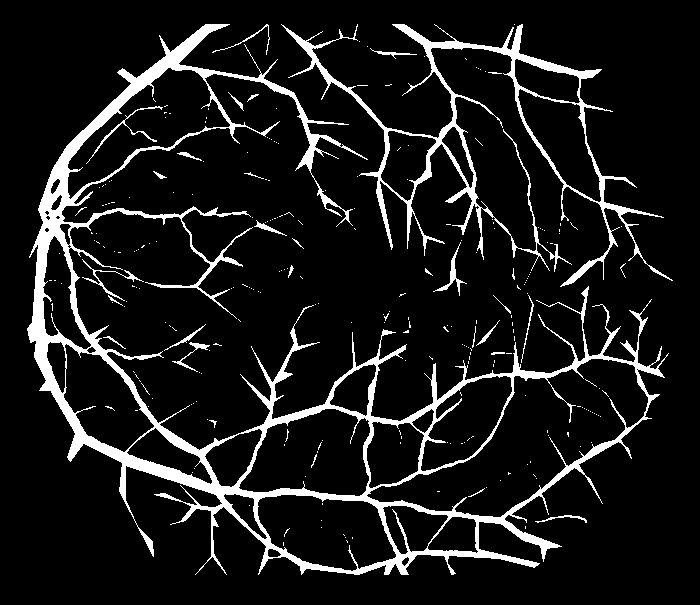

Supplement: Supplementary file 1 — Additional file 1. Generated noisy label maps. [file 12880_2021_732_MOESM1_ESM.zip › Noisy_label_maps/STARE/LV3/im0081.png]

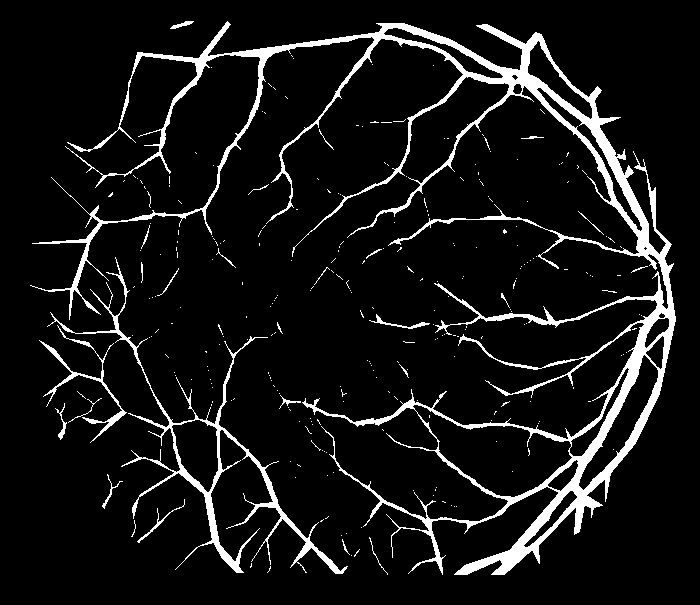

Supplement: Supplementary file 1 — Additional file 1. Generated noisy label maps. [file 12880_2021_732_MOESM1_ESM.zip › Noisy_label_maps/STARE/LV3/im0082.png]

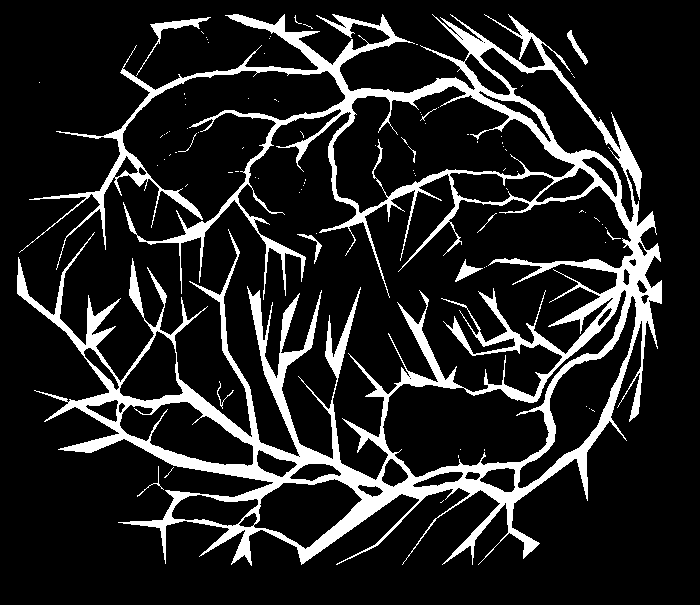

Supplement: Supplementary file 1 — Additional file 1. Generated noisy label maps. [file 12880_2021_732_MOESM1_ESM.zip › Noisy_label_maps/STARE/LV3/im0139.png]

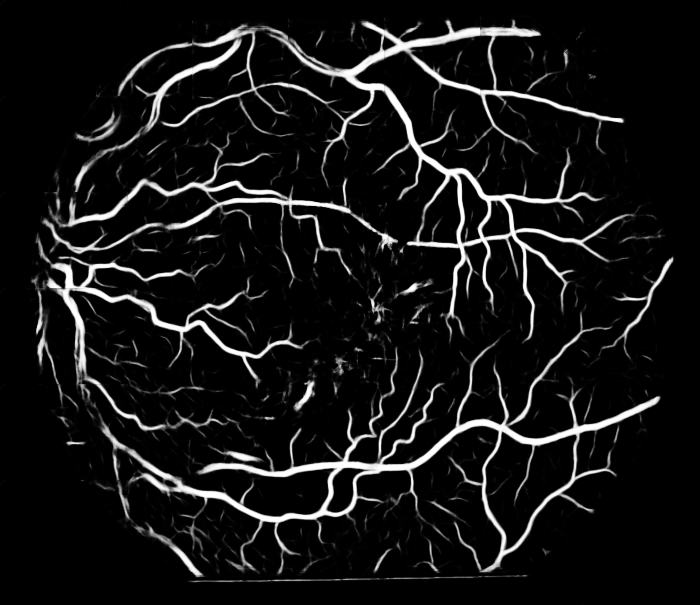

Supplement: Supplementary file 1 — Additional file 1. Generated noisy label maps. [file 12880_2021_732_MOESM1_ESM.zip › Noisy_label_maps/STARE/Pseudo/01_prediction_mix.png]

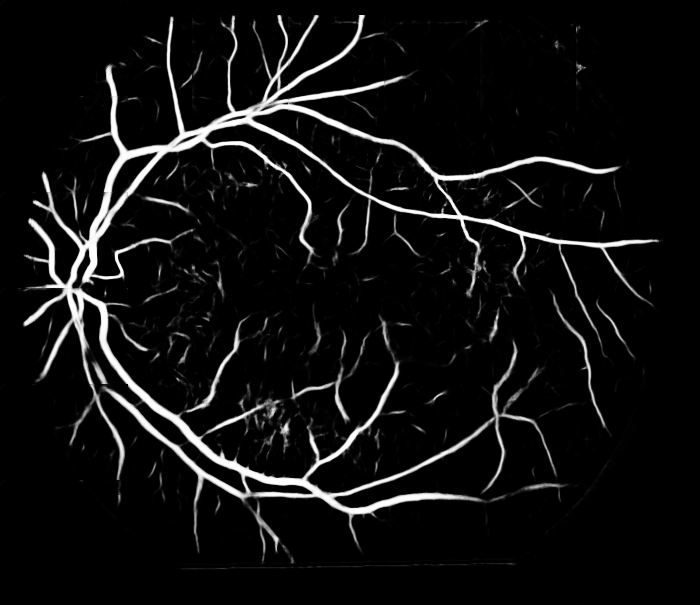

Supplement: Supplementary file 1 — Additional file 1. Generated noisy label maps. [file 12880_2021_732_MOESM1_ESM.zip › Noisy_label_maps/STARE/Pseudo/02_prediction_mix.png]

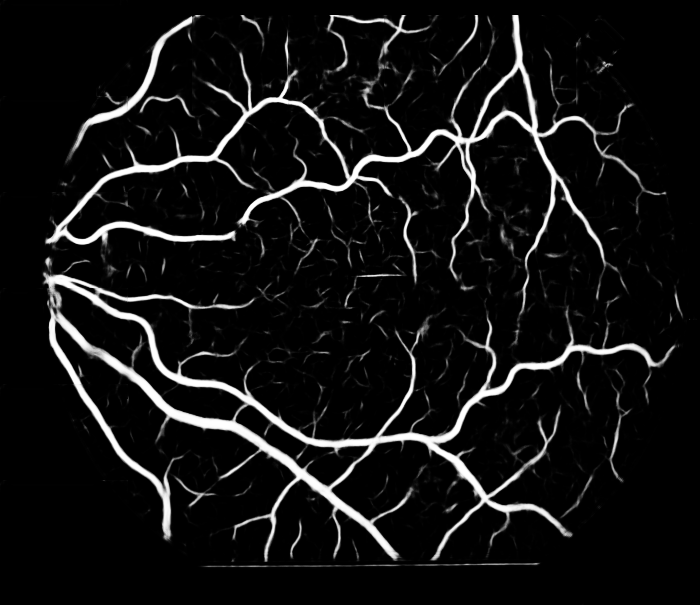

Supplement: Supplementary file 1 — Additional file 1. Generated noisy label maps. [file 12880_2021_732_MOESM1_ESM.zip › Noisy_label_maps/STARE/Pseudo/03_prediction_mix.png]

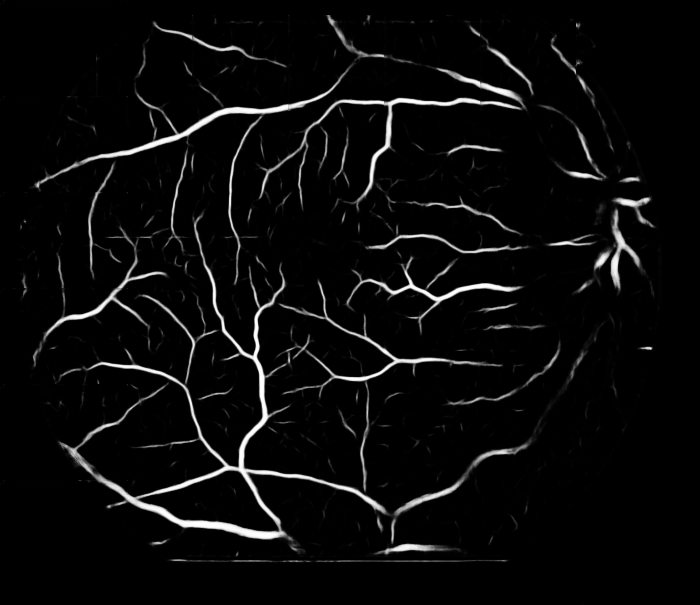

Supplement: Supplementary file 1 — Additional file 1. Generated noisy label maps. [file 12880_2021_732_MOESM1_ESM.zip › Noisy_label_maps/STARE/Pseudo/04_prediction_mix.png]

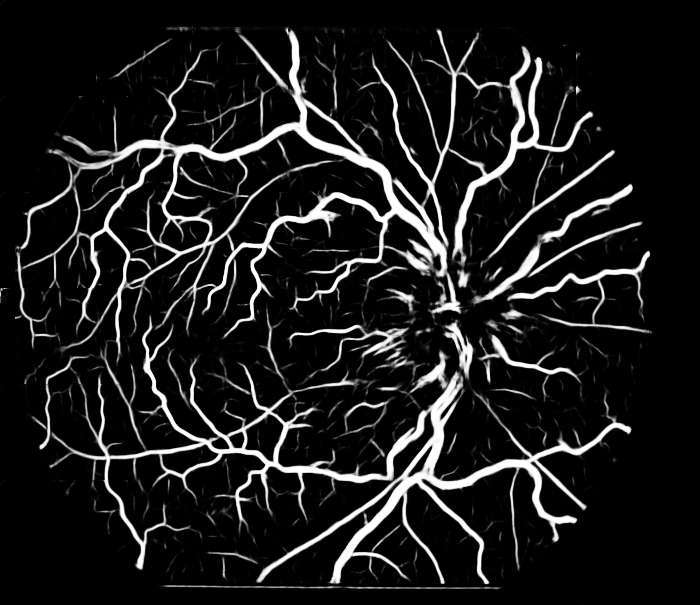

Supplement: Supplementary file 1 — Additional file 1. Generated noisy label maps. [file 12880_2021_732_MOESM1_ESM.zip › Noisy_label_maps/STARE/Pseudo/05_prediction_mix.png]

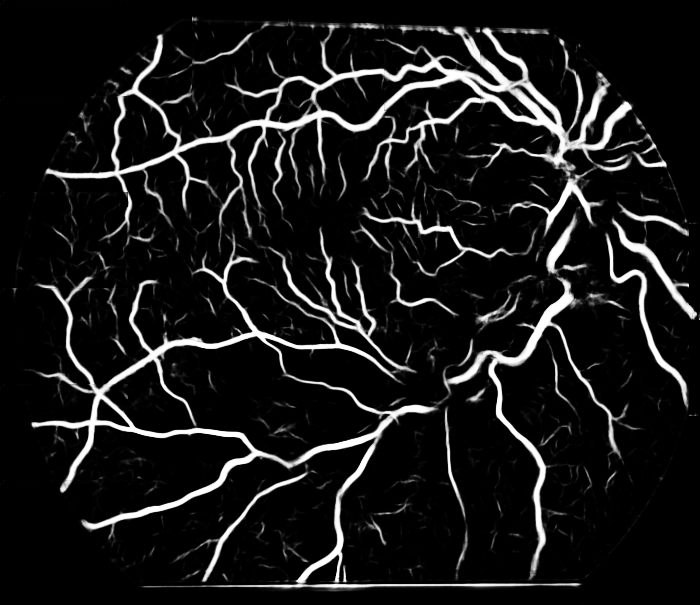

Supplement: Supplementary file 1 — Additional file 1. Generated noisy label maps. [file 12880_2021_732_MOESM1_ESM.zip › Noisy_label_maps/STARE/Pseudo/06_prediction_mix.png]

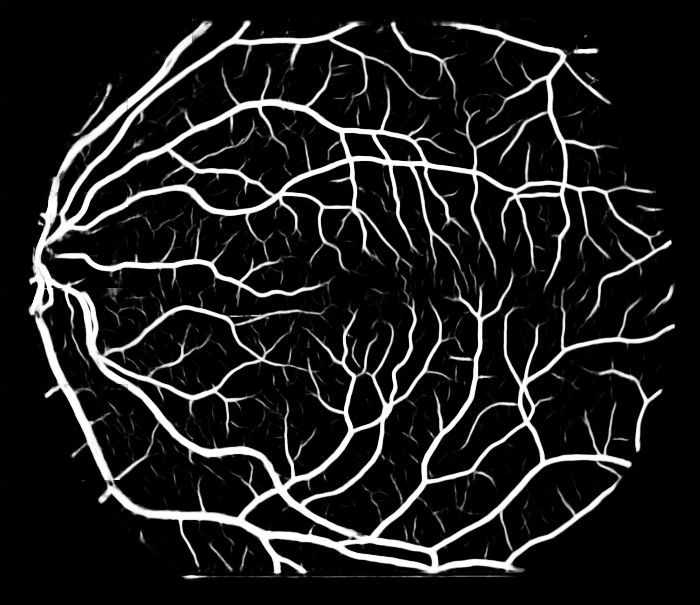

Supplement: Supplementary file 1 — Additional file 1. Generated noisy label maps. [file 12880_2021_732_MOESM1_ESM.zip › Noisy_label_maps/STARE/Pseudo/07_prediction_mix.png]

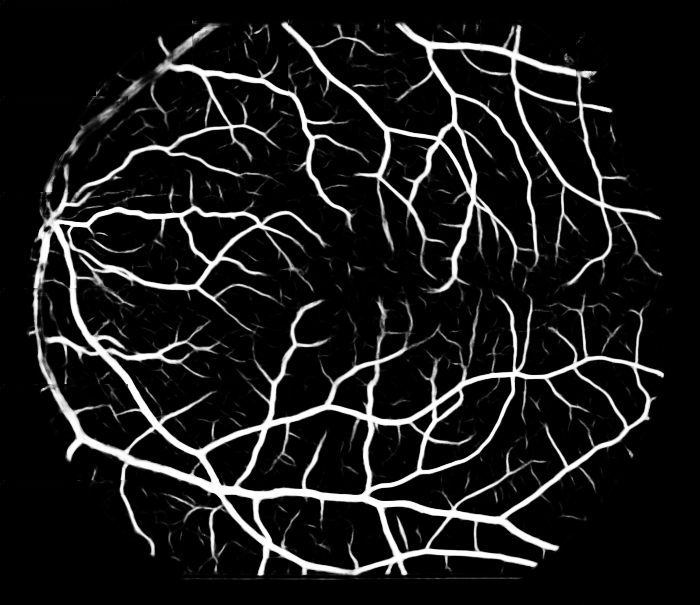

Supplement: Supplementary file 1 — Additional file 1. Generated noisy label maps. [file 12880_2021_732_MOESM1_ESM.zip › Noisy_label_maps/STARE/Pseudo/08_prediction_mix.png]

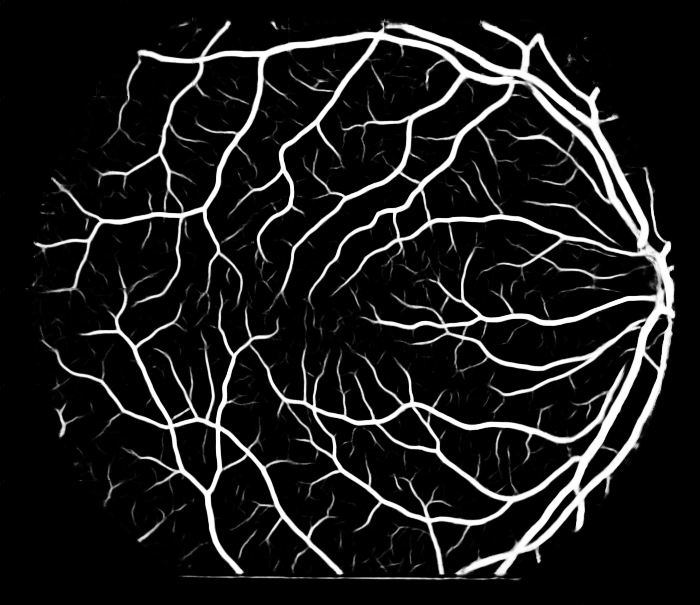

Supplement: Supplementary file 1 — Additional file 1. Generated noisy label maps. [file 12880_2021_732_MOESM1_ESM.zip › Noisy_label_maps/STARE/Pseudo/09_prediction_mix.png]

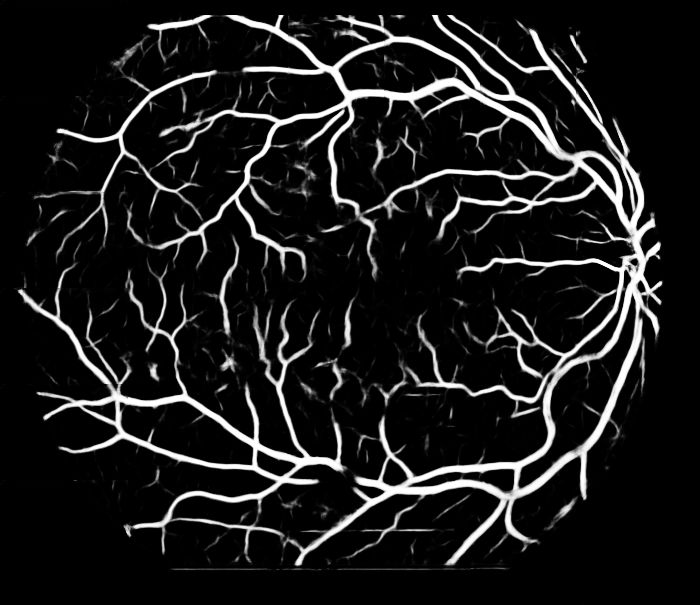

Supplement: Supplementary file 1 — Additional file 1. Generated noisy label maps. [file 12880_2021_732_MOESM1_ESM.zip › Noisy_label_maps/STARE/Pseudo/10_prediction_mix.png]

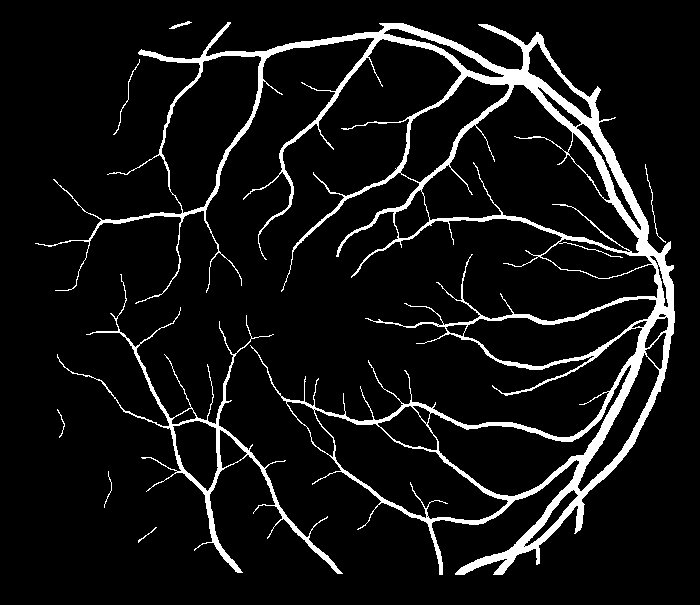

Supplement: Supplementary file 1 — Additional file 1. Generated noisy label maps. [file 12880_2021_732_MOESM1_ESM.zip › Noisy_label_maps/STARE/Manual/im0082.png]

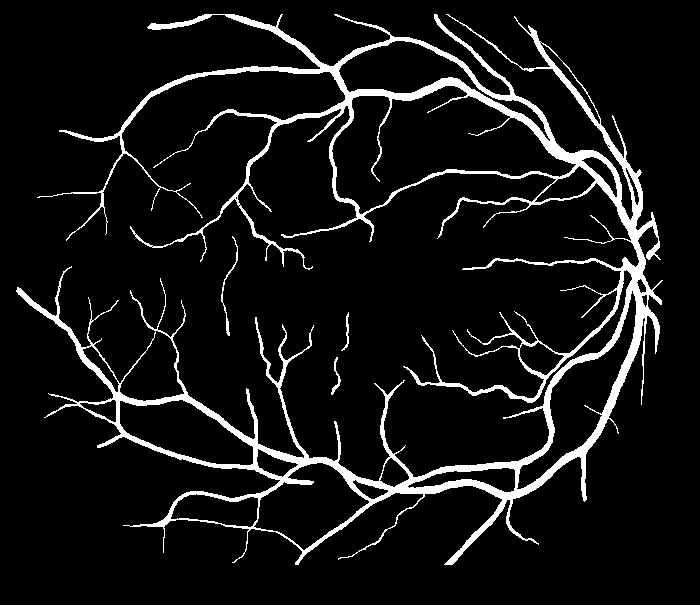

Supplement: Supplementary file 1 — Additional file 1. Generated noisy label maps. [file 12880_2021_732_MOESM1_ESM.zip › Noisy_label_maps/STARE/Manual/im0139.png]

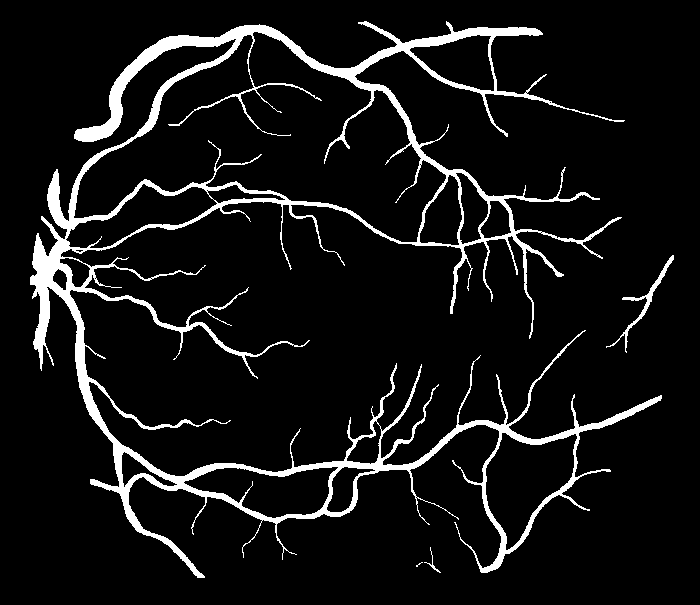

Supplement: Supplementary file 1 — Additional file 1. Generated noisy label maps. [file 12880_2021_732_MOESM1_ESM.zip › Noisy_label_maps/STARE/Manual/im0001.png]

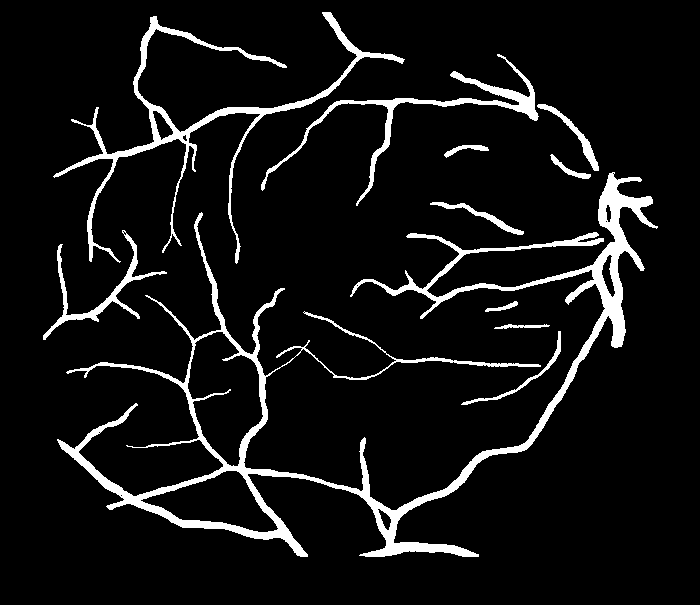

Supplement: Supplementary file 1 — Additional file 1. Generated noisy label maps. [file 12880_2021_732_MOESM1_ESM.zip › Noisy_label_maps/STARE/Manual/im0004.png]

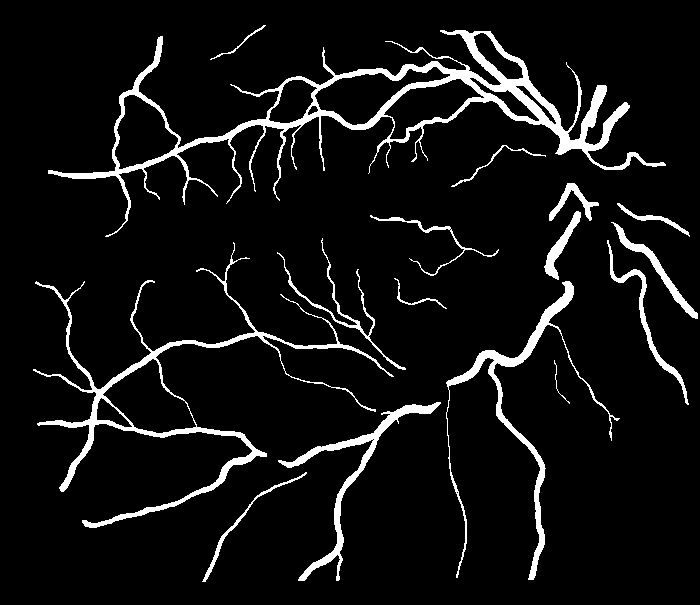

Supplement: Supplementary file 1 — Additional file 1. Generated noisy label maps. [file 12880_2021_732_MOESM1_ESM.zip › Noisy_label_maps/STARE/Manual/im0044.png]

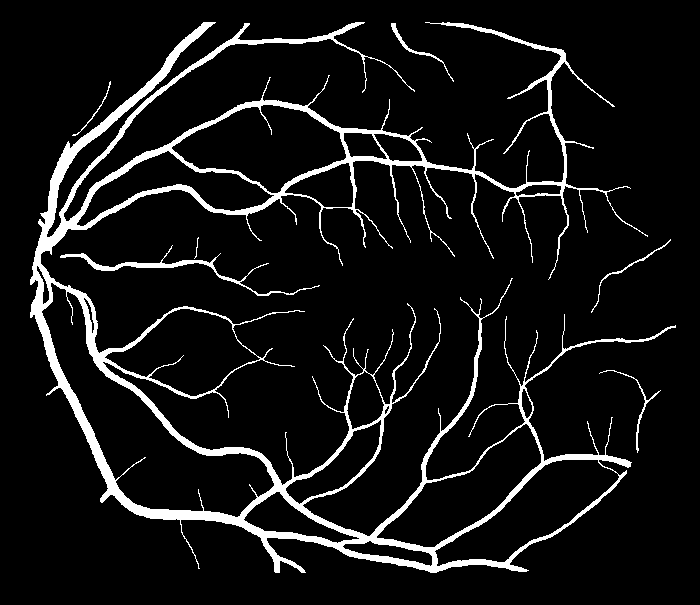

Supplement: Supplementary file 1 — Additional file 1. Generated noisy label maps. [file 12880_2021_732_MOESM1_ESM.zip › Noisy_label_maps/STARE/Manual/im0077.png]

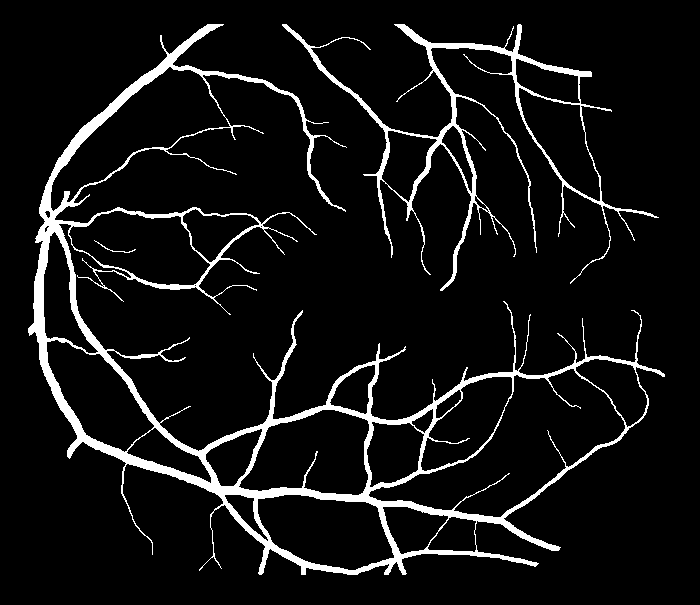

Supplement: Supplementary file 1 — Additional file 1. Generated noisy label maps. [file 12880_2021_732_MOESM1_ESM.zip › Noisy_label_maps/STARE/Manual/im0081.png]

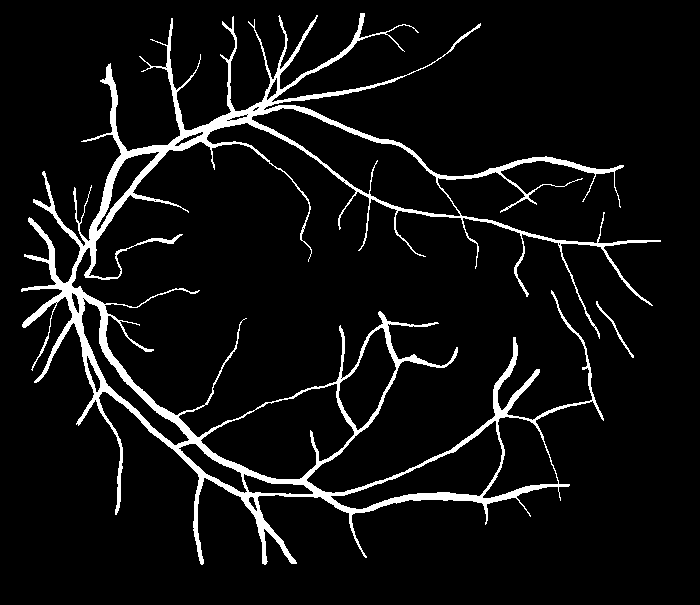

Supplement: Supplementary file 1 — Additional file 1. Generated noisy label maps. [file 12880_2021_732_MOESM1_ESM.zip › Noisy_label_maps/STARE/Manual/im0002.png]

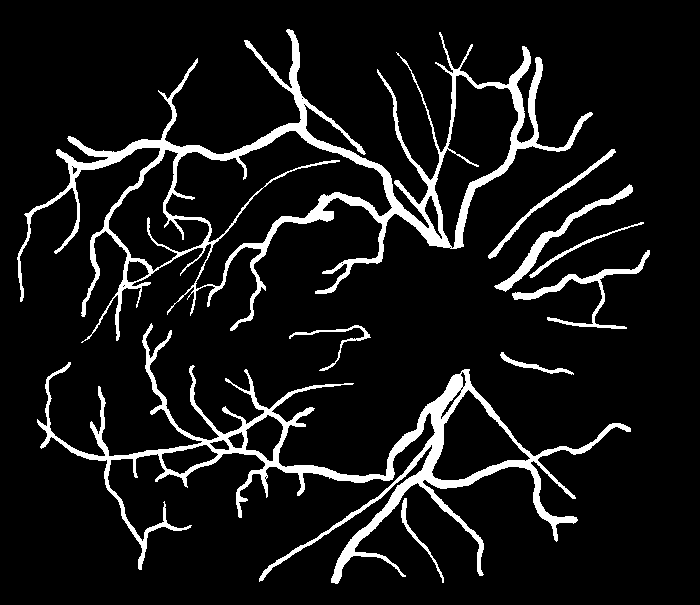

Supplement: Supplementary file 1 — Additional file 1. Generated noisy label maps. [file 12880_2021_732_MOESM1_ESM.zip › Noisy_label_maps/STARE/Manual/im0005.png]

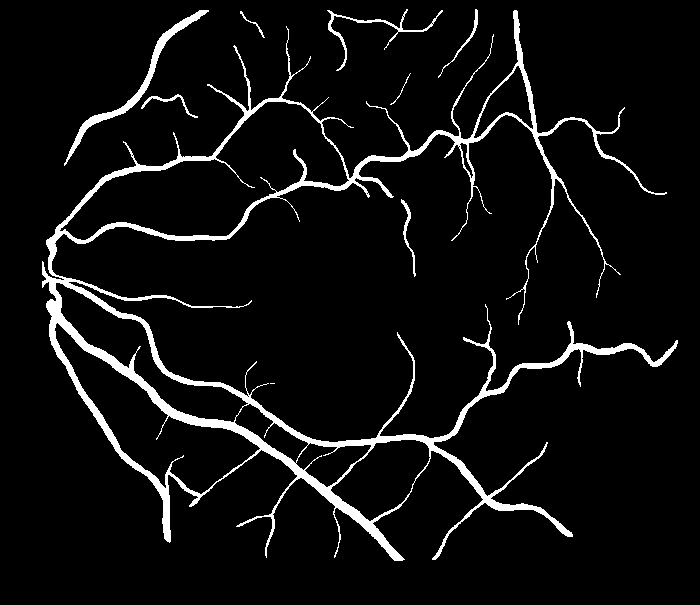

Supplement: Supplementary file 1 — Additional file 1. Generated noisy label maps. [file 12880_2021_732_MOESM1_ESM.zip › Noisy_label_maps/STARE/Manual/im0003.png]

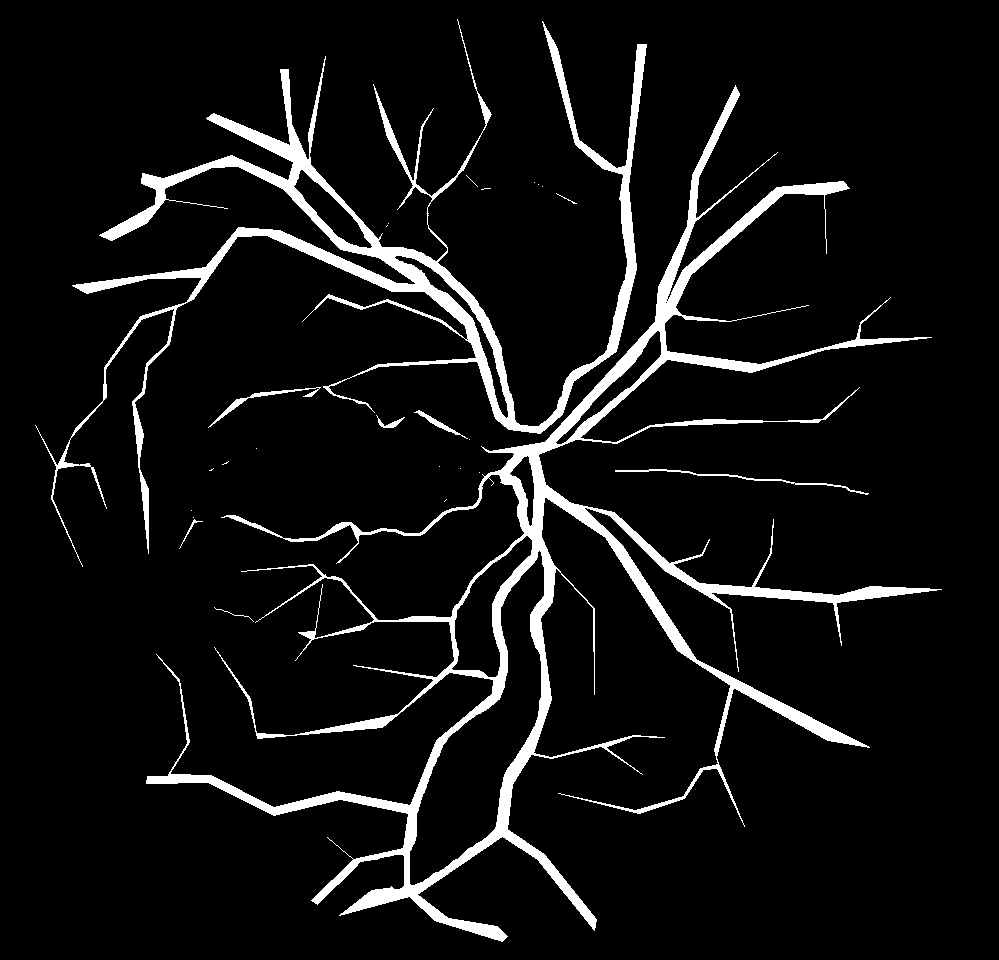

Supplement: Supplementary file 1 — Additional file 1. Generated noisy label maps. [file 12880_2021_732_MOESM1_ESM.zip › Noisy_label_maps/CHASE/LV1/Image_06R_1stHO.png]

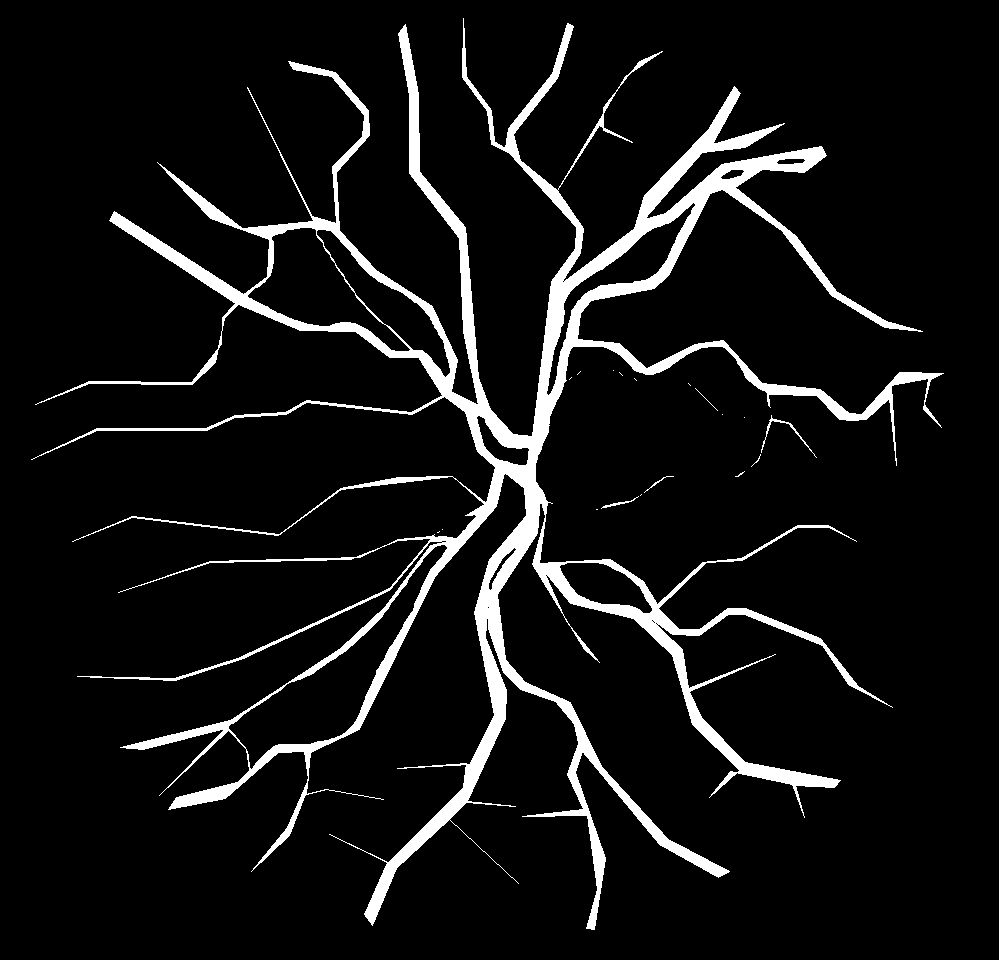

Supplement: Supplementary file 1 — Additional file 1. Generated noisy label maps. [file 12880_2021_732_MOESM1_ESM.zip › Noisy_label_maps/CHASE/LV1/Image_05L_1stHO.png]

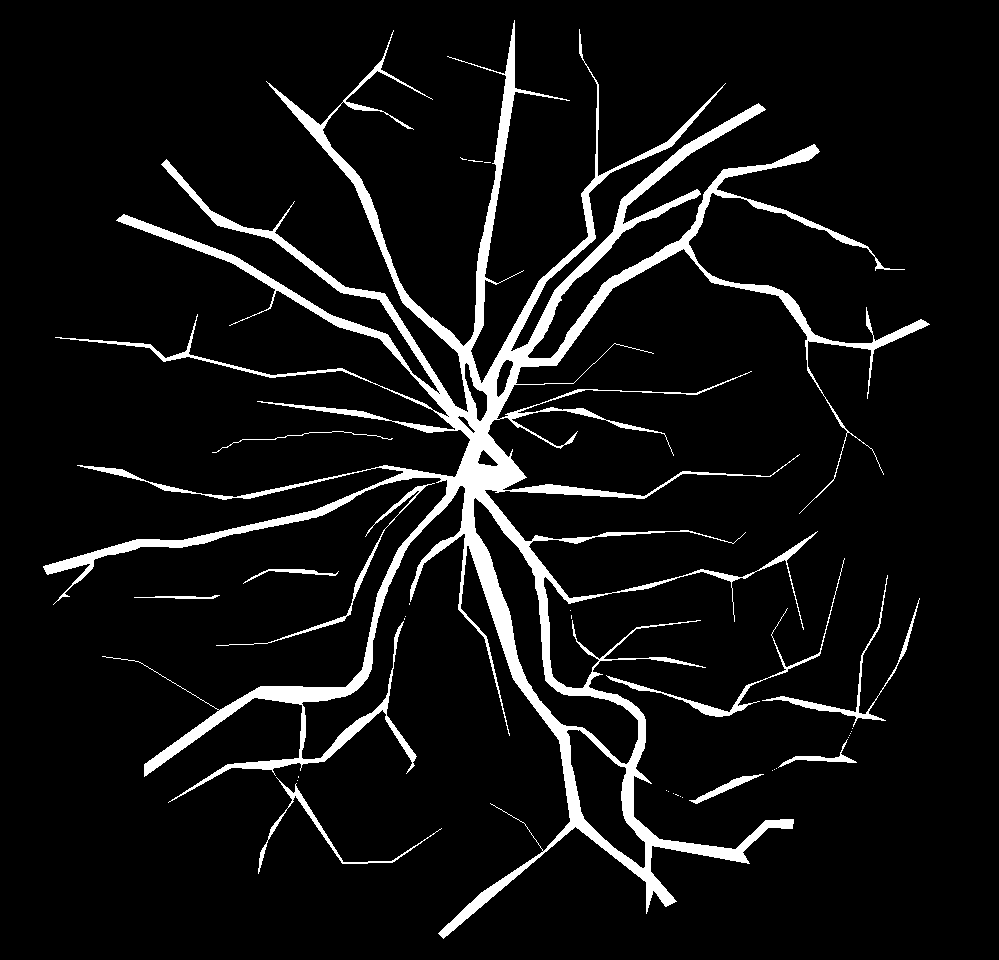

Supplement: Supplementary file 1 — Additional file 1. Generated noisy label maps. [file 12880_2021_732_MOESM1_ESM.zip › Noisy_label_maps/CHASE/LV1/Image_03L_1stHO.png]

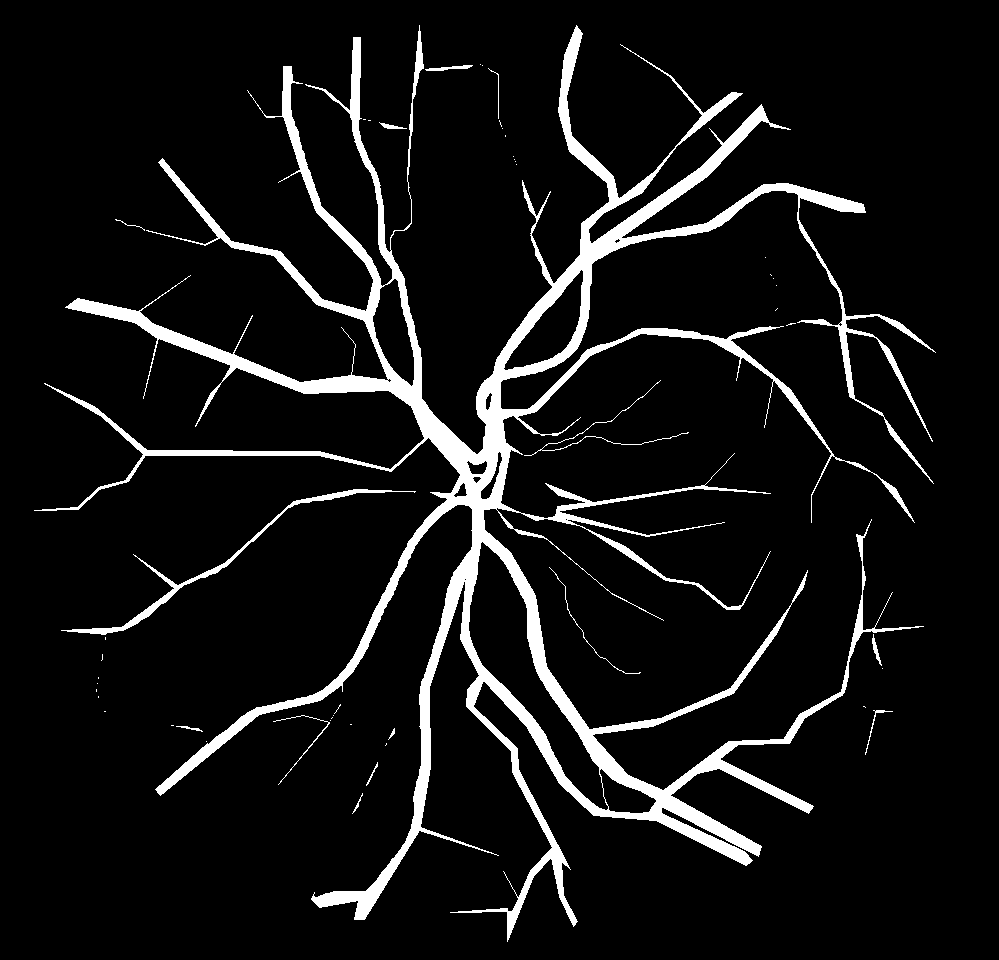

Supplement: Supplementary file 1 — Additional file 1. Generated noisy label maps. [file 12880_2021_732_MOESM1_ESM.zip › Noisy_label_maps/CHASE/LV1/Image_02L_1stHO.png]

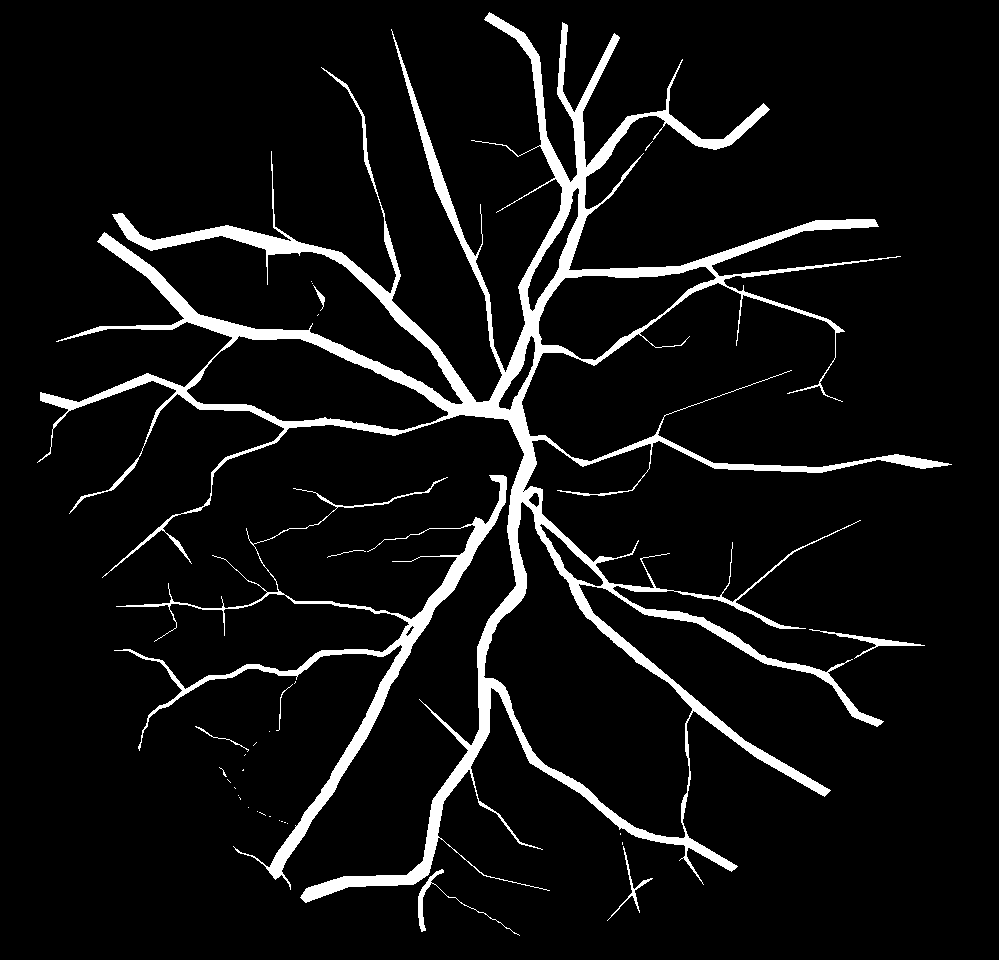

Supplement: Supplementary file 1 — Additional file 1. Generated noisy label maps. [file 12880_2021_732_MOESM1_ESM.zip › Noisy_label_maps/CHASE/LV1/Image_07R_1stHO.png]

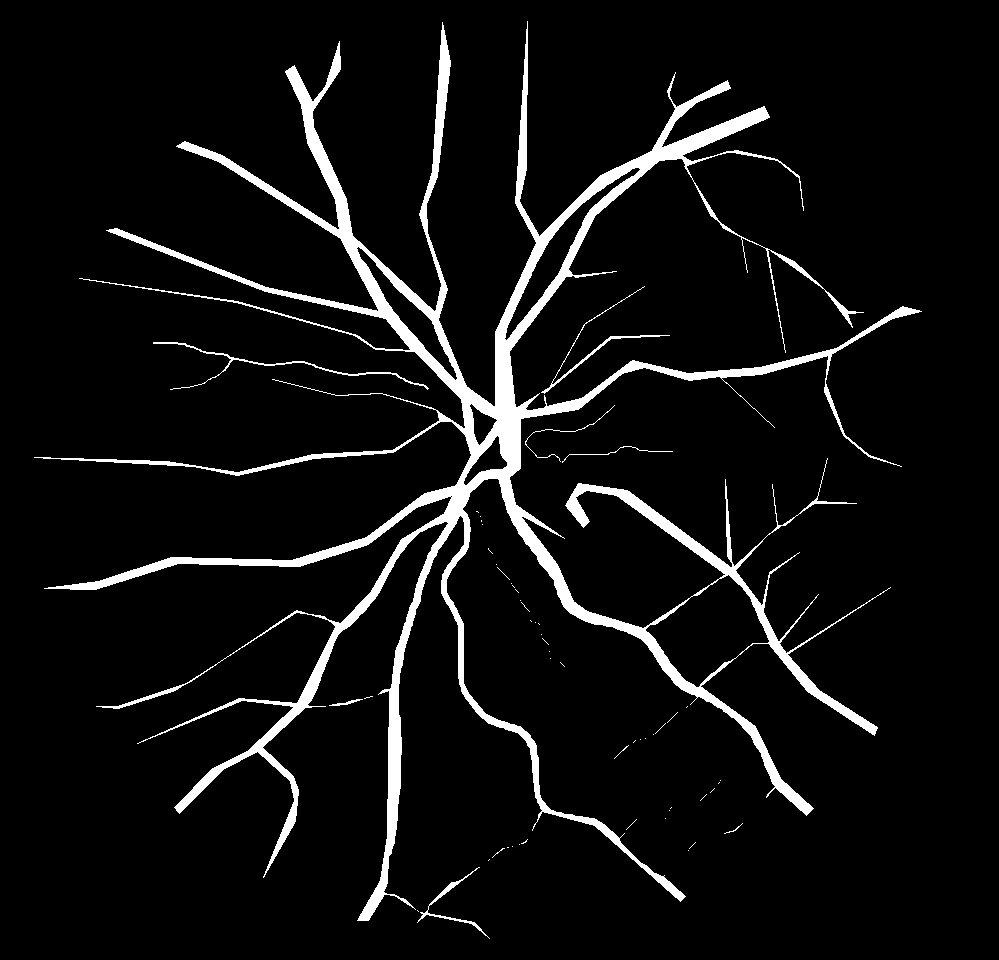

Supplement: Supplementary file 1 — Additional file 1. Generated noisy label maps. [file 12880_2021_732_MOESM1_ESM.zip › Noisy_label_maps/CHASE/LV1/Image_01L_1stHO.png]

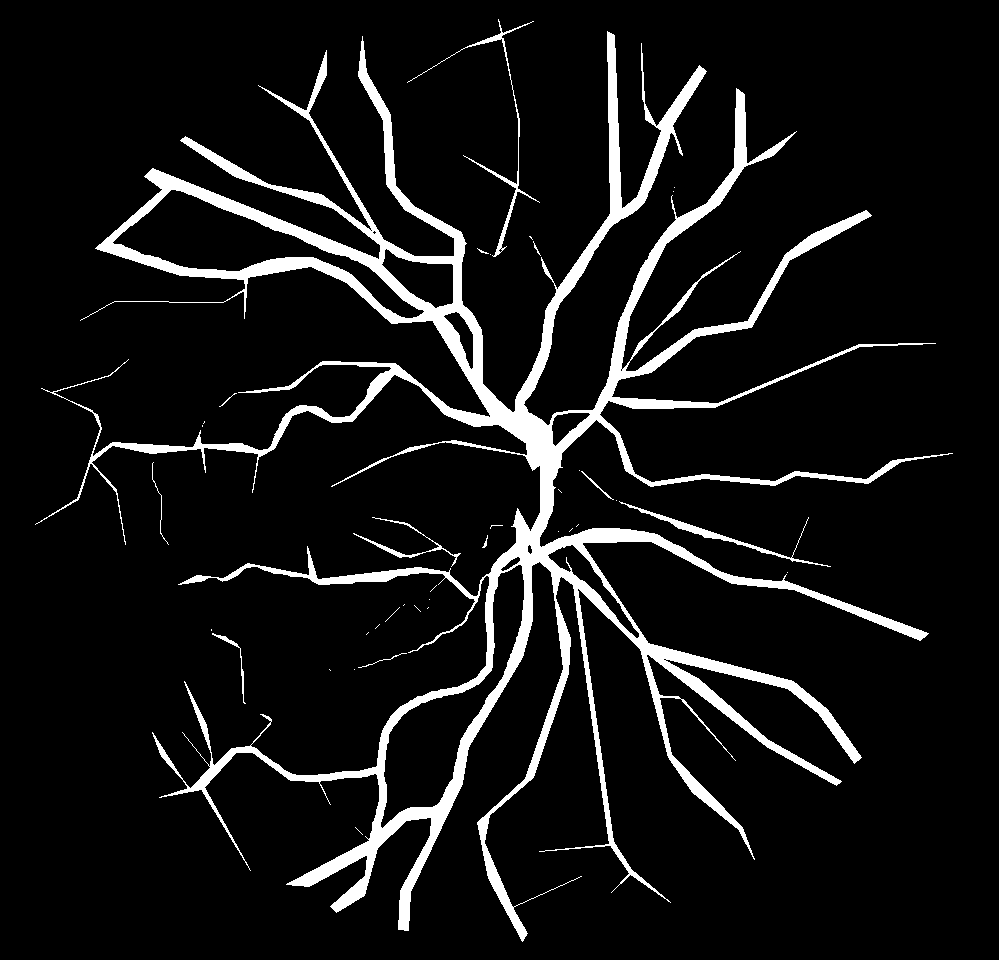

Supplement: Supplementary file 1 — Additional file 1. Generated noisy label maps. [file 12880_2021_732_MOESM1_ESM.zip › Noisy_label_maps/CHASE/LV1/Image_05R_1stHO.png]

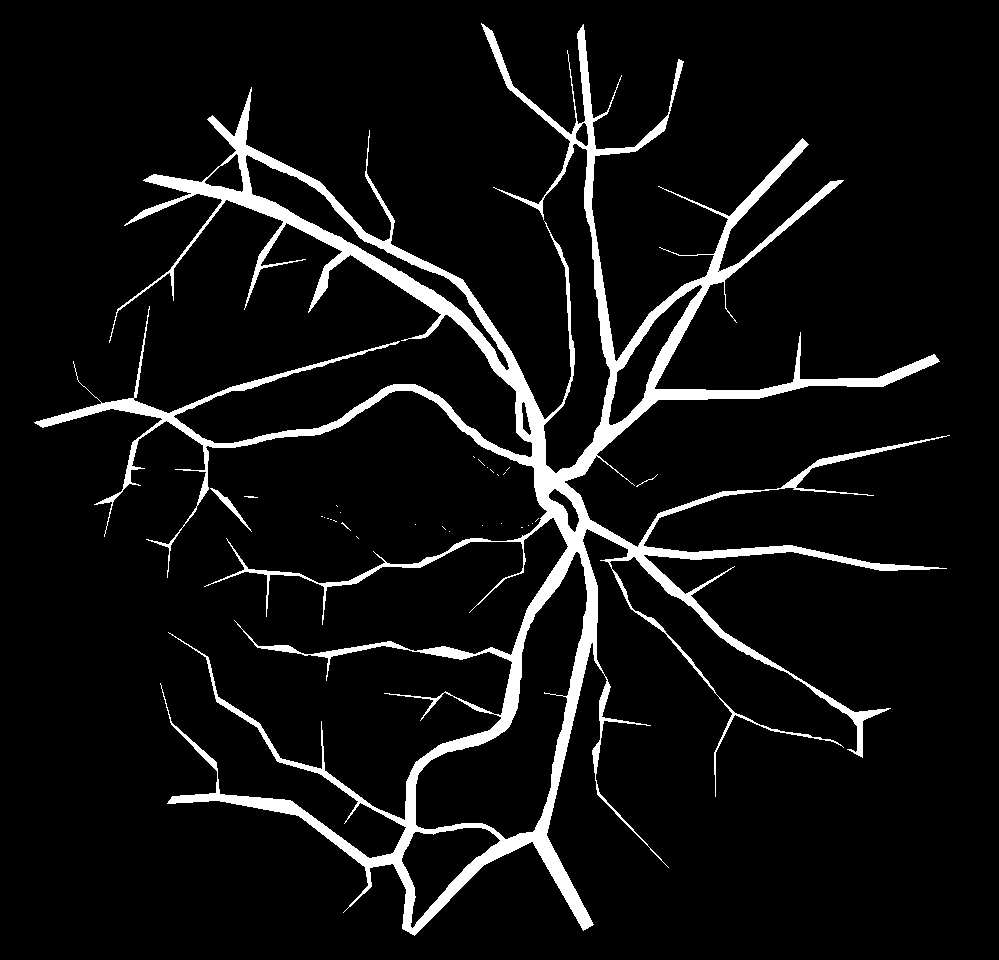

Supplement: Supplementary file 1 — Additional file 1. Generated noisy label maps. [file 12880_2021_732_MOESM1_ESM.zip › Noisy_label_maps/CHASE/LV1/Image_01R_1stHO.png]

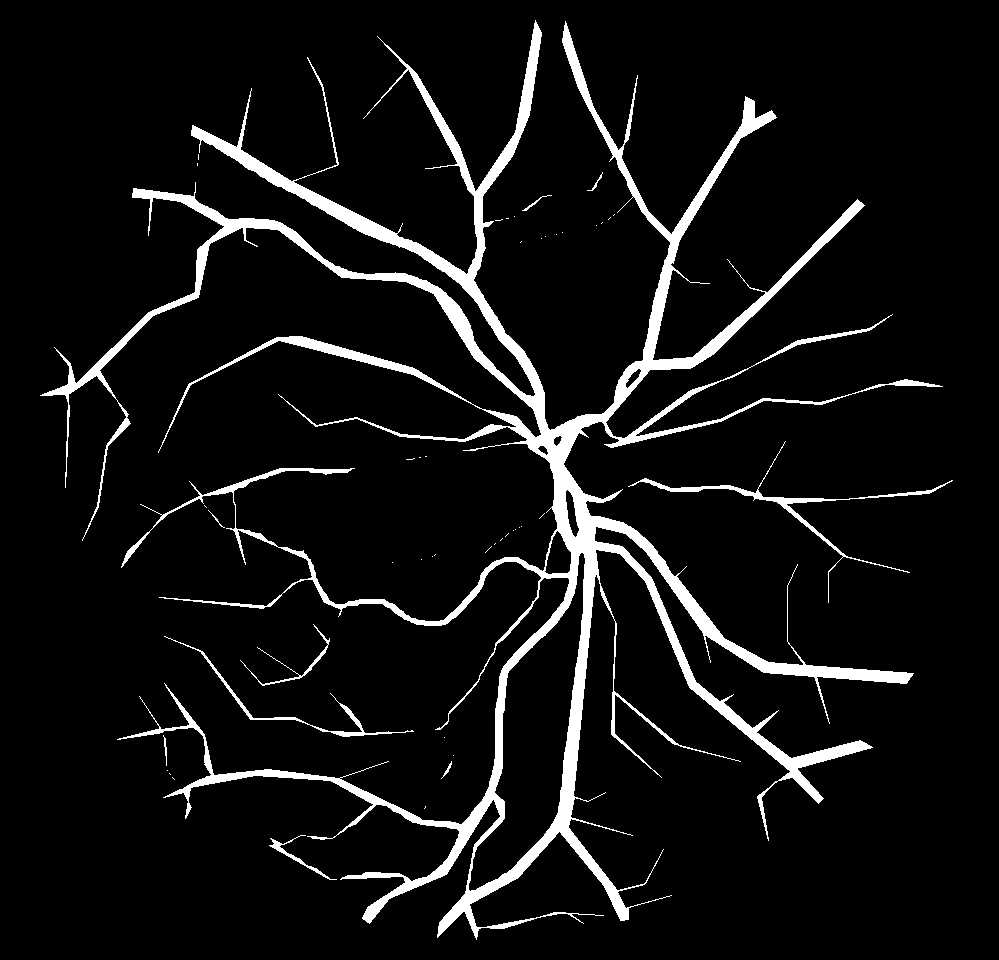

Supplement: Supplementary file 1 — Additional file 1. Generated noisy label maps. [file 12880_2021_732_MOESM1_ESM.zip › Noisy_label_maps/CHASE/LV1/Image_02R_1stHO.png]

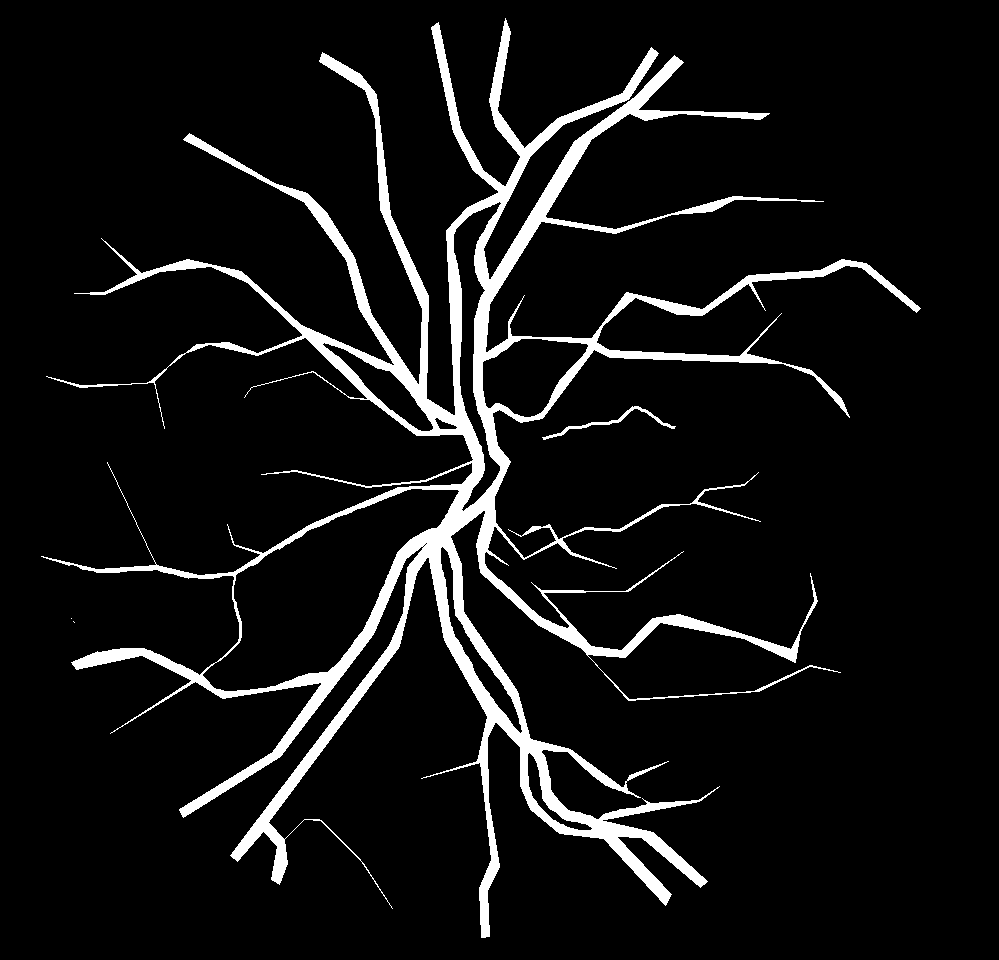

Supplement: Supplementary file 1 — Additional file 1. Generated noisy label maps. [file 12880_2021_732_MOESM1_ESM.zip › Noisy_label_maps/CHASE/LV1/Image_04L_1stHO.png]

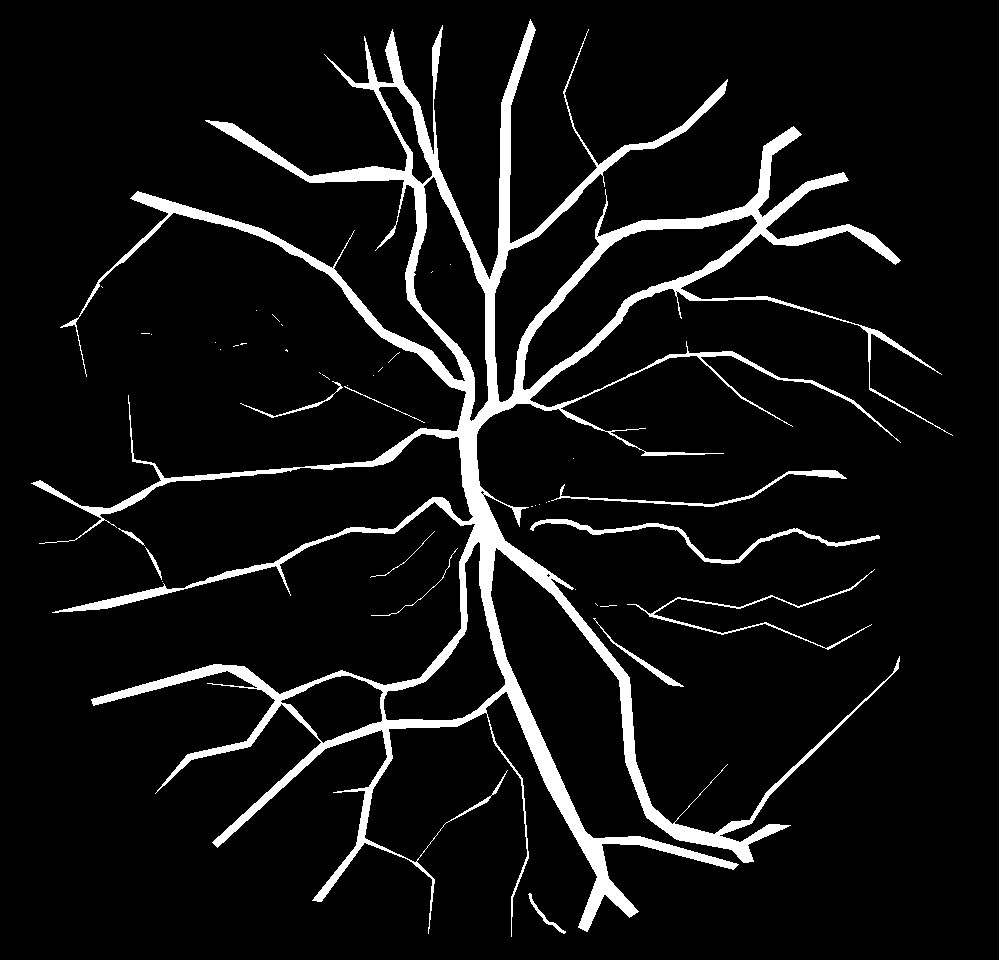

Supplement: Supplementary file 1 — Additional file 1. Generated noisy label maps. [file 12880_2021_732_MOESM1_ESM.zip › Noisy_label_maps/CHASE/LV1/Image_07L_1stHO.png]

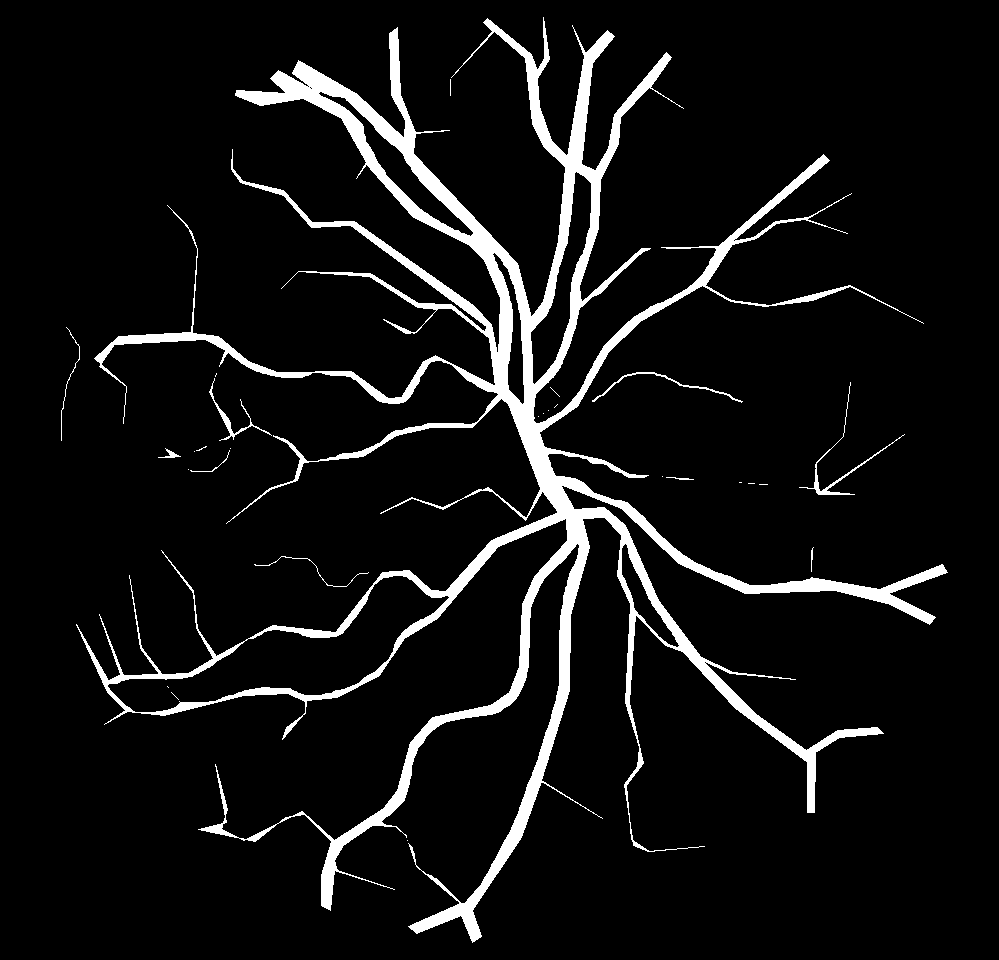

Supplement: Supplementary file 1 — Additional file 1. Generated noisy label maps. [file 12880_2021_732_MOESM1_ESM.zip › Noisy_label_maps/CHASE/LV1/Image_04R_1stHO.png]

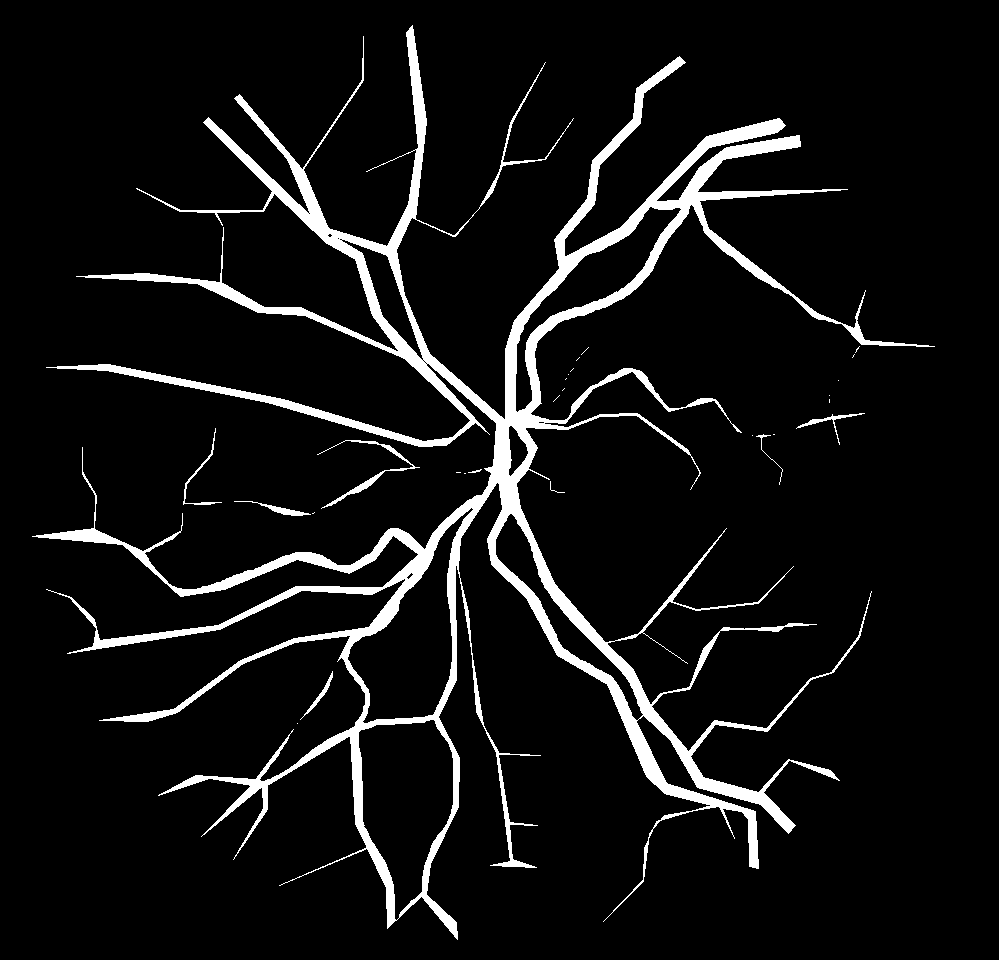

Supplement: Supplementary file 1 — Additional file 1. Generated noisy label maps. [file 12880_2021_732_MOESM1_ESM.zip › Noisy_label_maps/CHASE/LV1/Image_06L_1stHO.png]

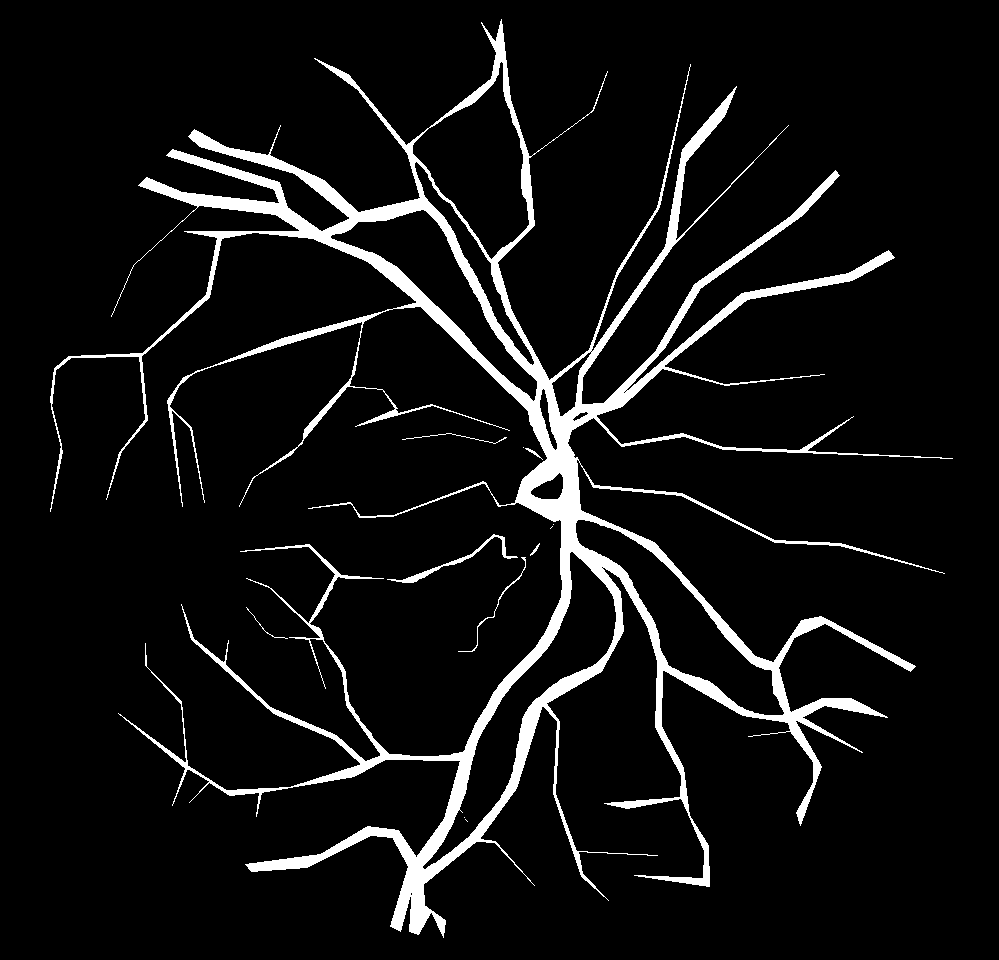

Supplement: Supplementary file 1 — Additional file 1. Generated noisy label maps. [file 12880_2021_732_MOESM1_ESM.zip › Noisy_label_maps/CHASE/LV1/Image_03R_1stHO.png]

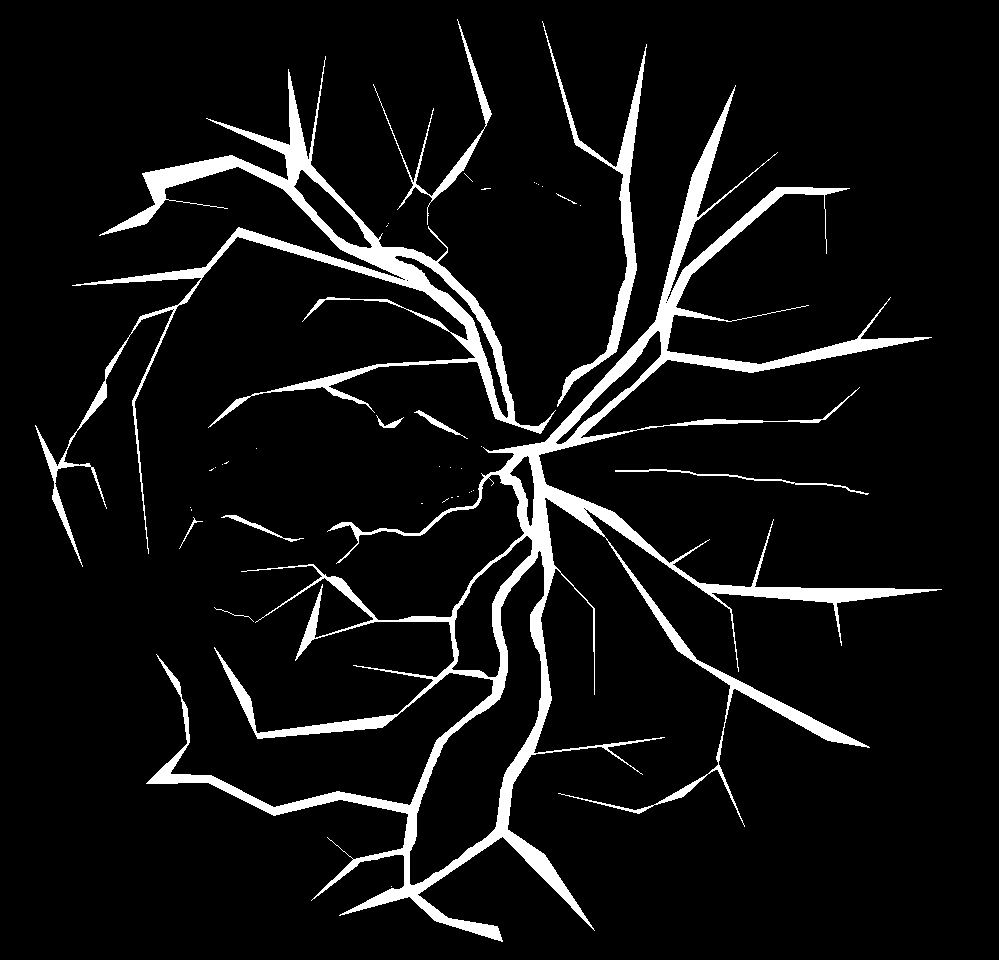

Supplement: Supplementary file 1 — Additional file 1. Generated noisy label maps. [file 12880_2021_732_MOESM1_ESM.zip › Noisy_label_maps/CHASE/LV3/Image_06R_1stHO.png]

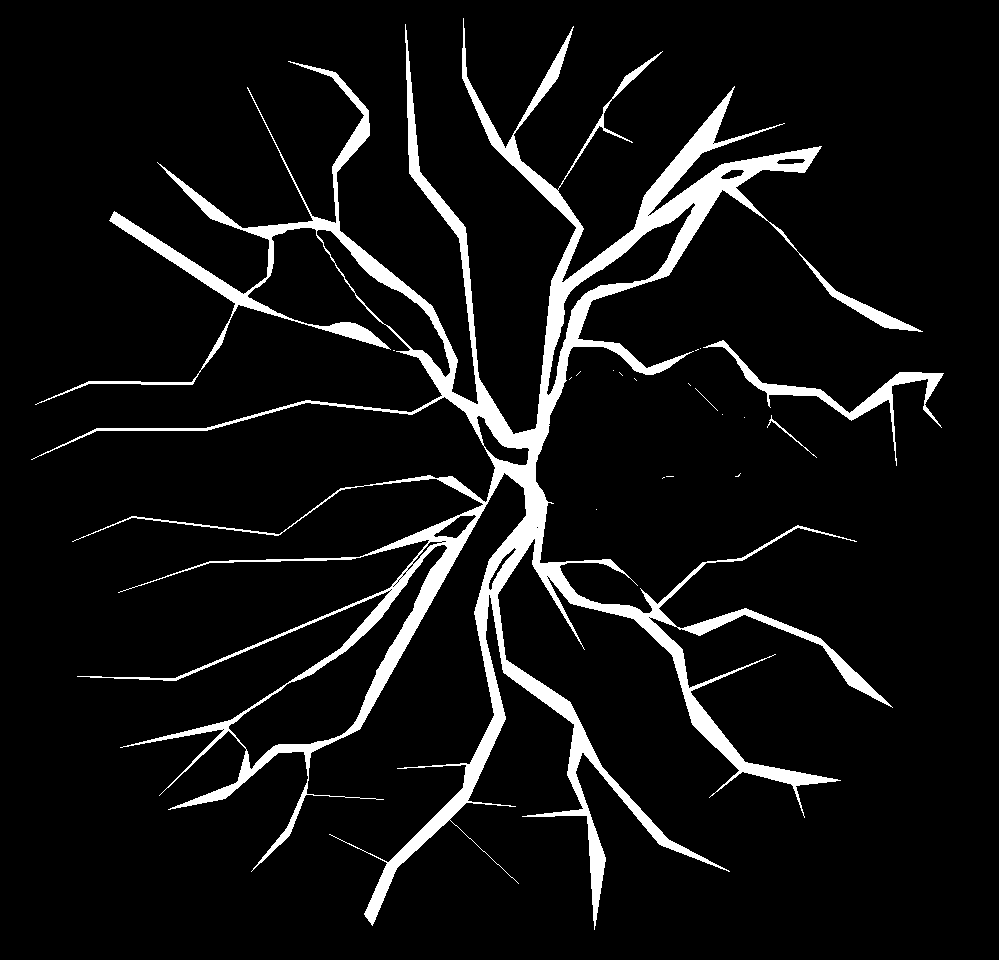

Supplement: Supplementary file 1 — Additional file 1. Generated noisy label maps. [file 12880_2021_732_MOESM1_ESM.zip › Noisy_label_maps/CHASE/LV3/Image_05L_1stHO.png]

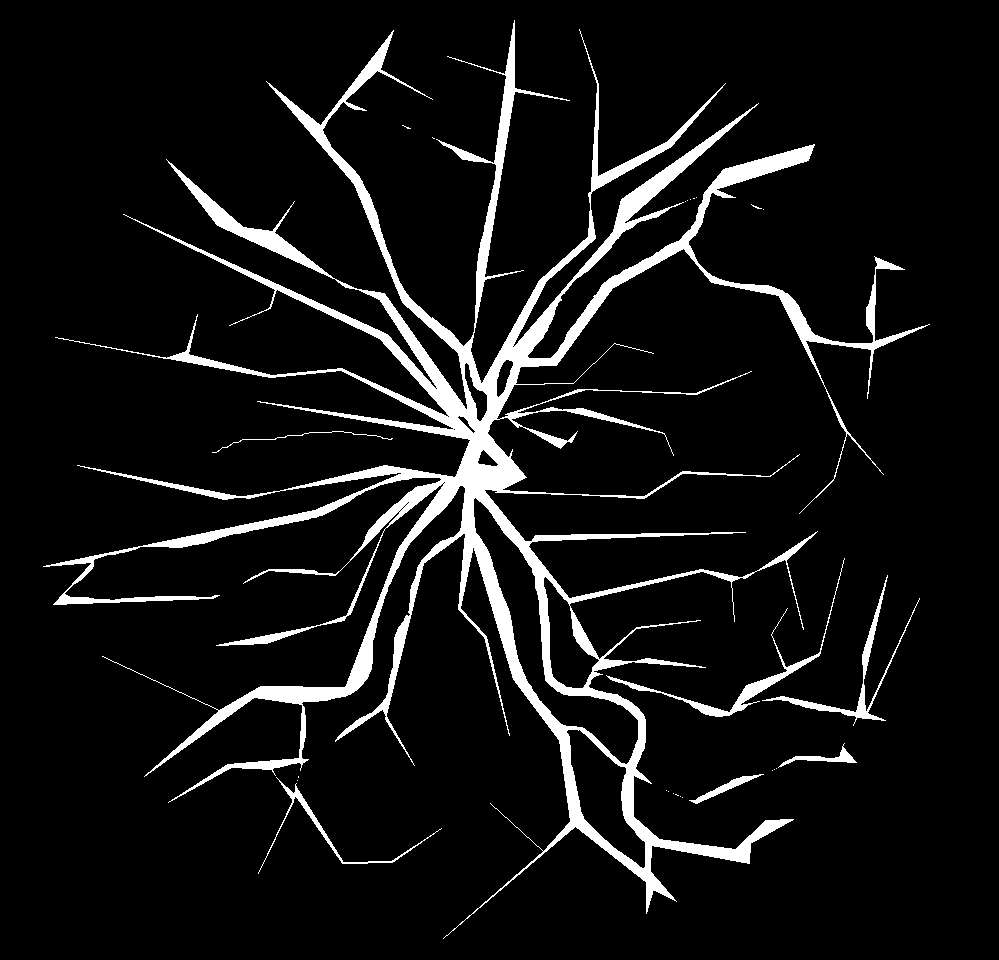

Supplement: Supplementary file 1 — Additional file 1. Generated noisy label maps. [file 12880_2021_732_MOESM1_ESM.zip › Noisy_label_maps/CHASE/LV3/Image_03L_1stHO.png]

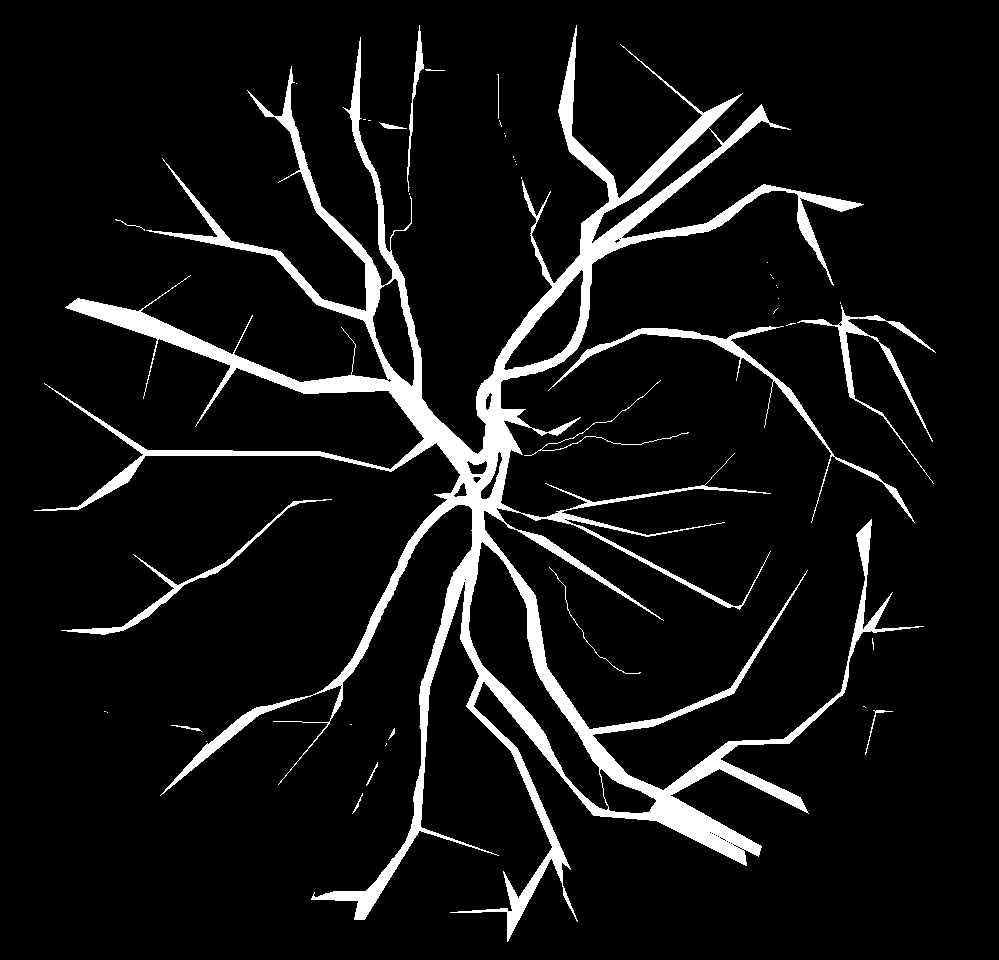

Supplement: Supplementary file 1 — Additional file 1. Generated noisy label maps. [file 12880_2021_732_MOESM1_ESM.zip › Noisy_label_maps/CHASE/LV3/Image_02L_1stHO.png]

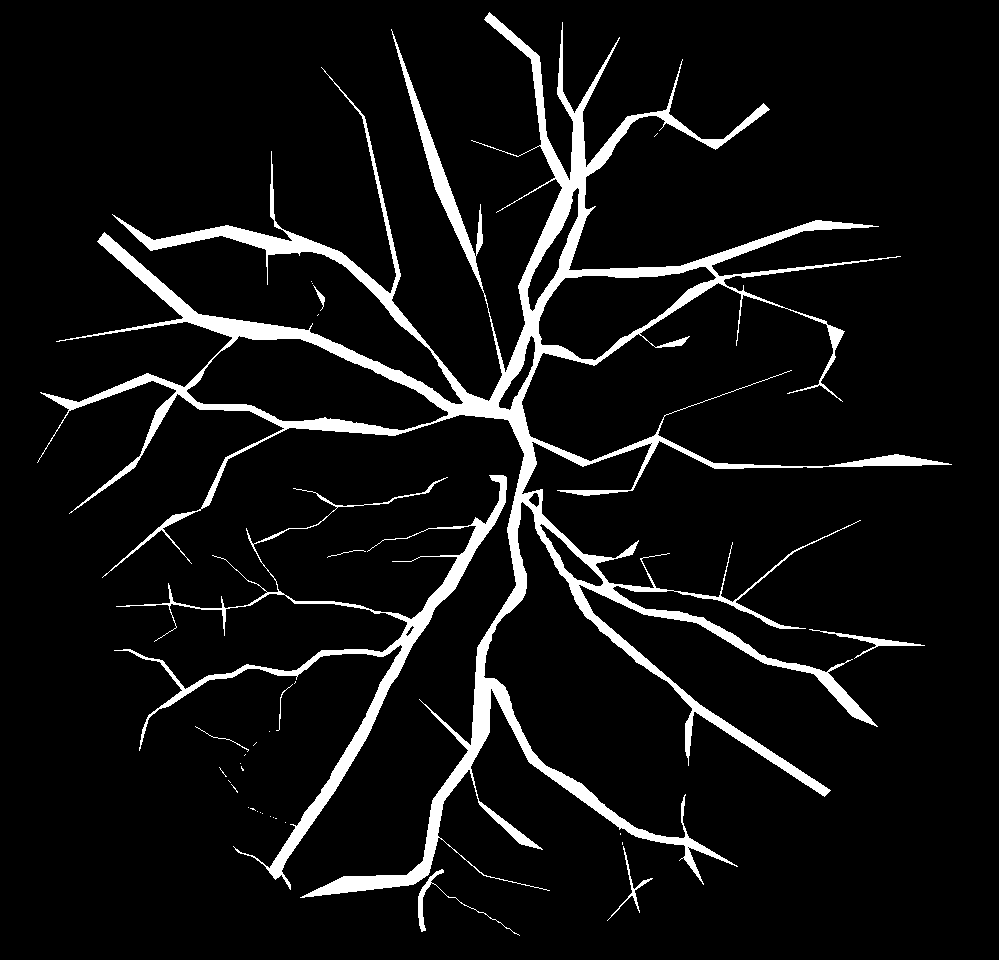

Supplement: Supplementary file 1 — Additional file 1. Generated noisy label maps. [file 12880_2021_732_MOESM1_ESM.zip › Noisy_label_maps/CHASE/LV3/Image_07R_1stHO.png]

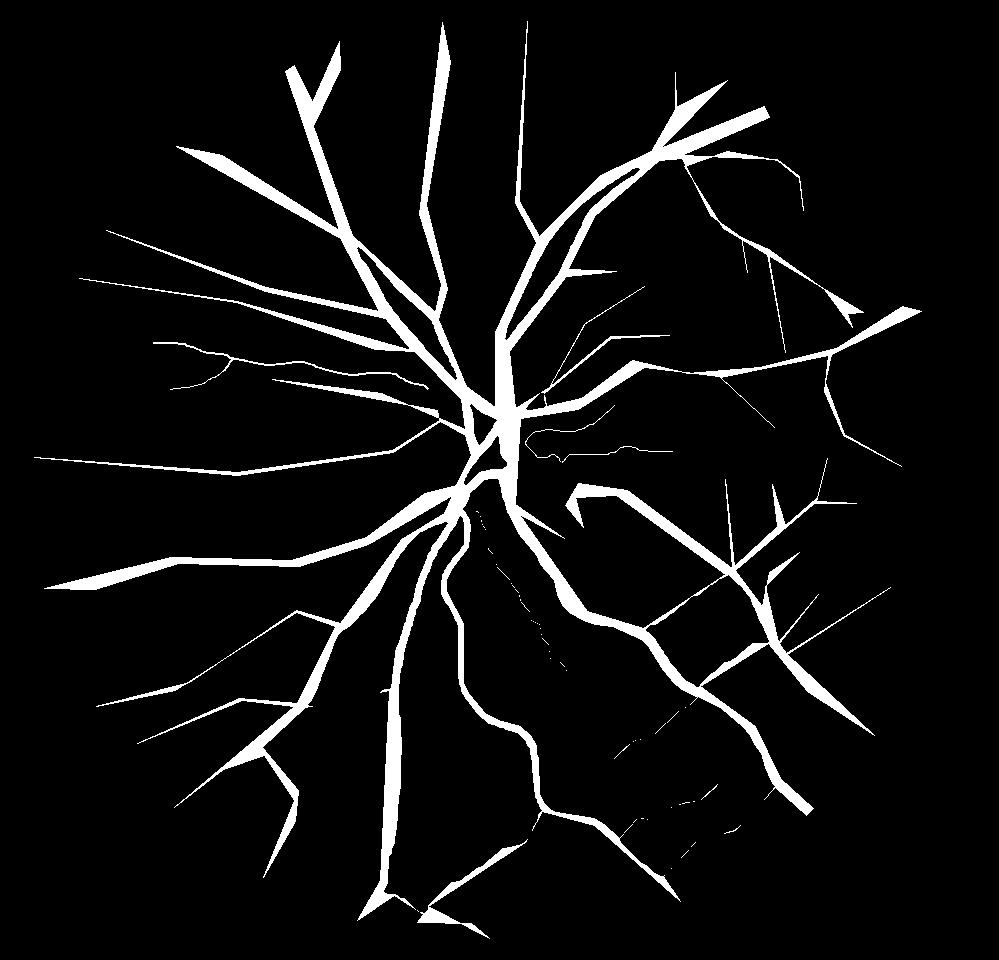

Supplement: Supplementary file 1 — Additional file 1. Generated noisy label maps. [file 12880_2021_732_MOESM1_ESM.zip › Noisy_label_maps/CHASE/LV3/Image_01L_1stHO.png]

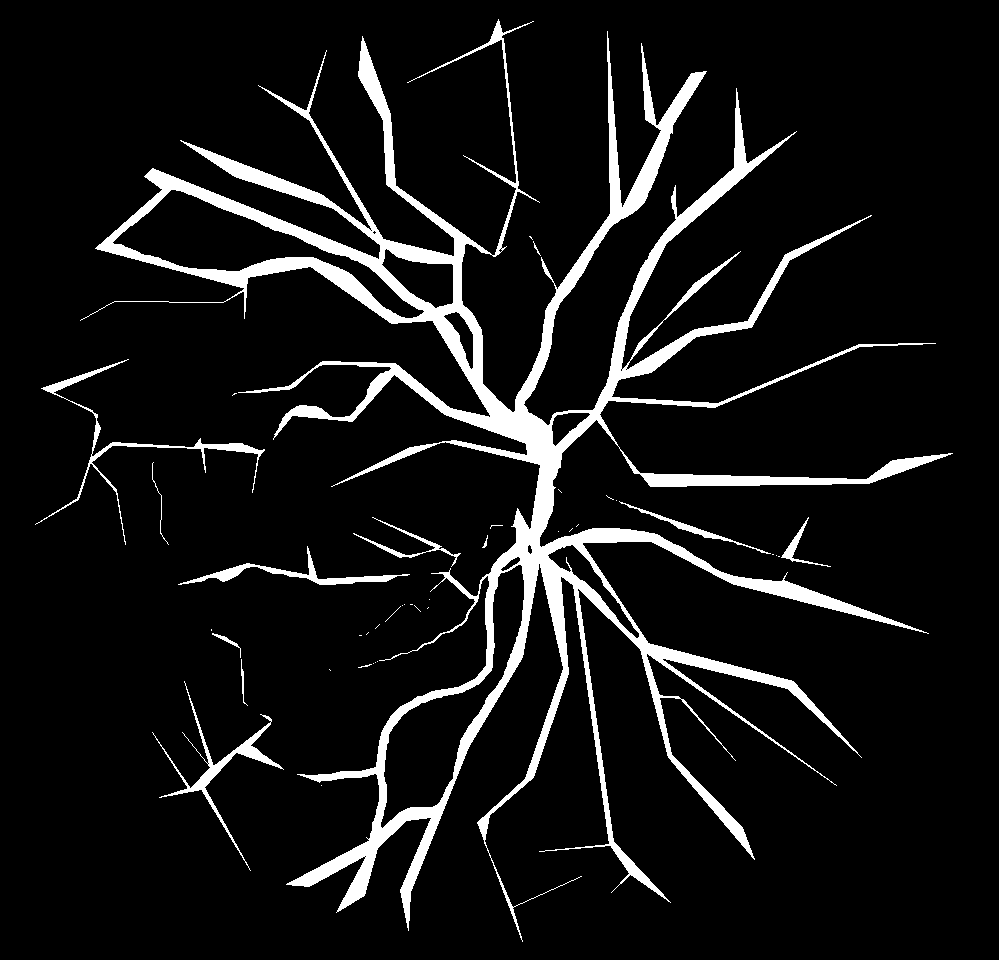

Supplement: Supplementary file 1 — Additional file 1. Generated noisy label maps. [file 12880_2021_732_MOESM1_ESM.zip › Noisy_label_maps/CHASE/LV3/Image_05R_1stHO.png]

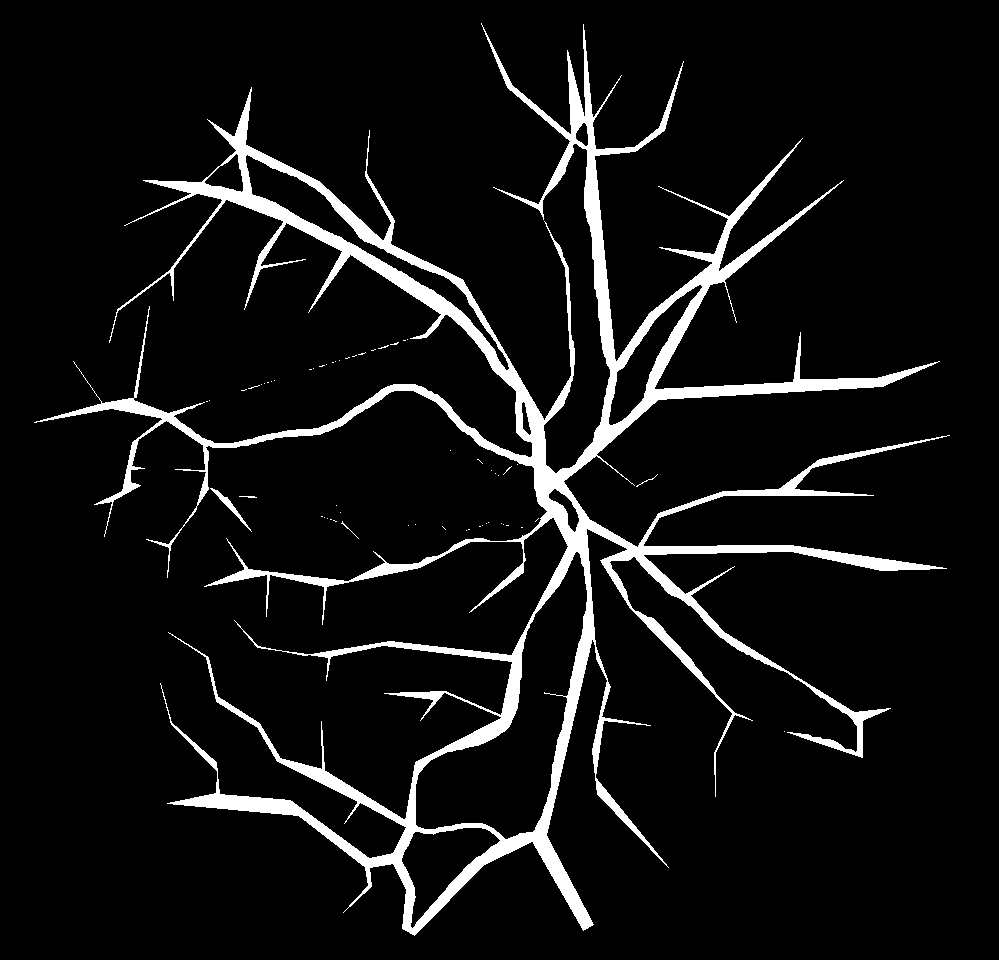

Supplement: Supplementary file 1 — Additional file 1. Generated noisy label maps. [file 12880_2021_732_MOESM1_ESM.zip › Noisy_label_maps/CHASE/LV3/Image_01R_1stHO.png]

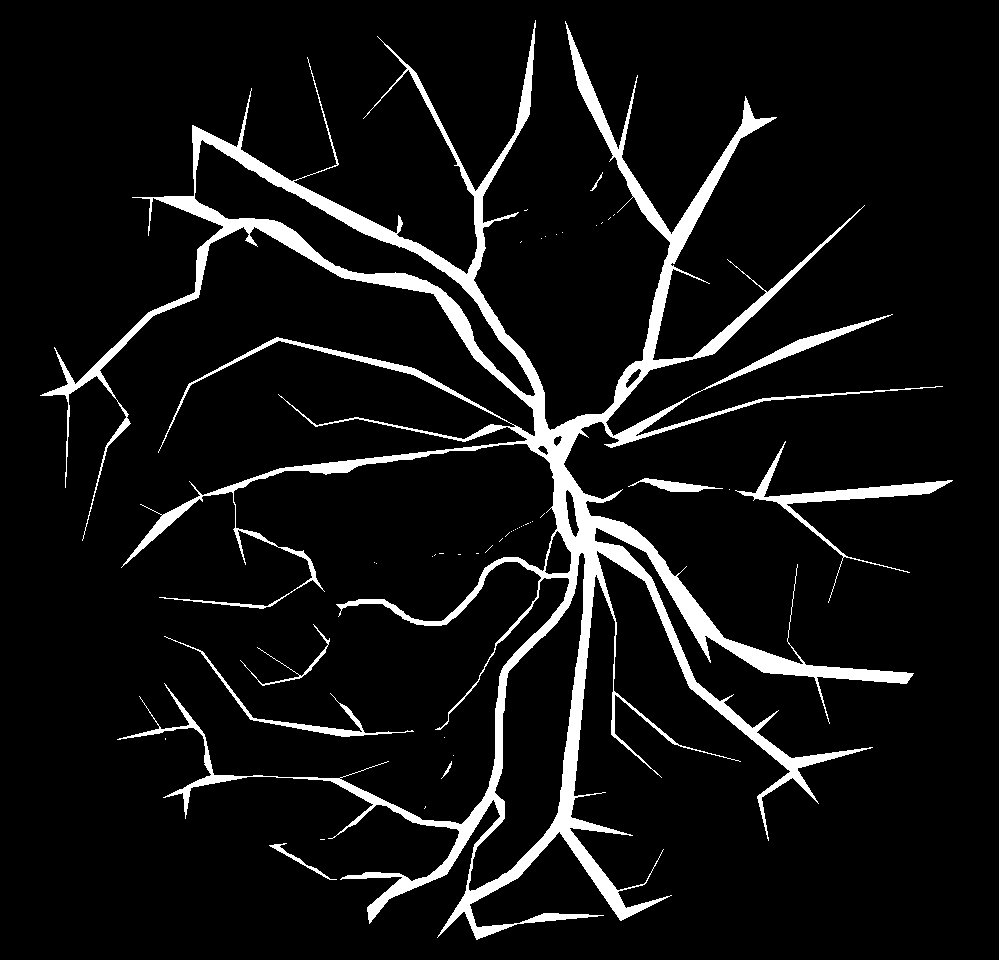

Supplement: Supplementary file 1 — Additional file 1. Generated noisy label maps. [file 12880_2021_732_MOESM1_ESM.zip › Noisy_label_maps/CHASE/LV3/Image_02R_1stHO.png]

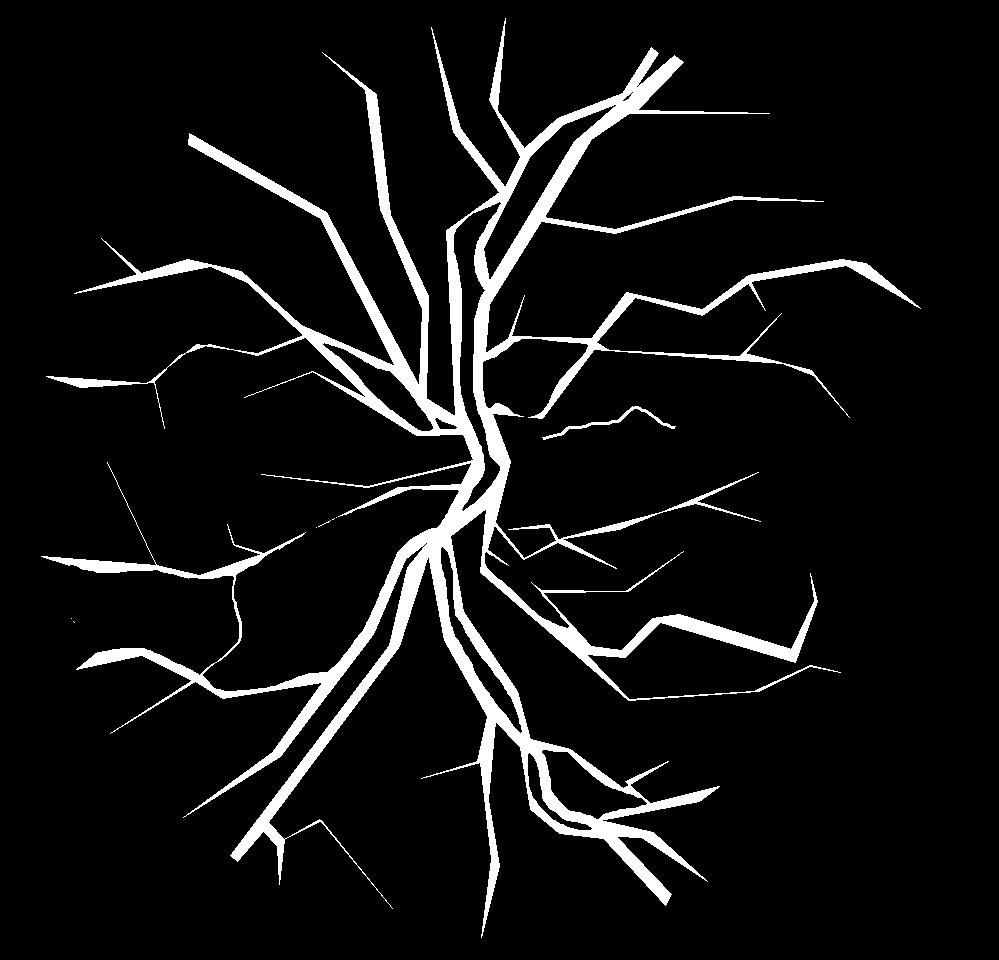

Supplement: Supplementary file 1 — Additional file 1. Generated noisy label maps. [file 12880_2021_732_MOESM1_ESM.zip › Noisy_label_maps/CHASE/LV3/Image_04L_1stHO.png]

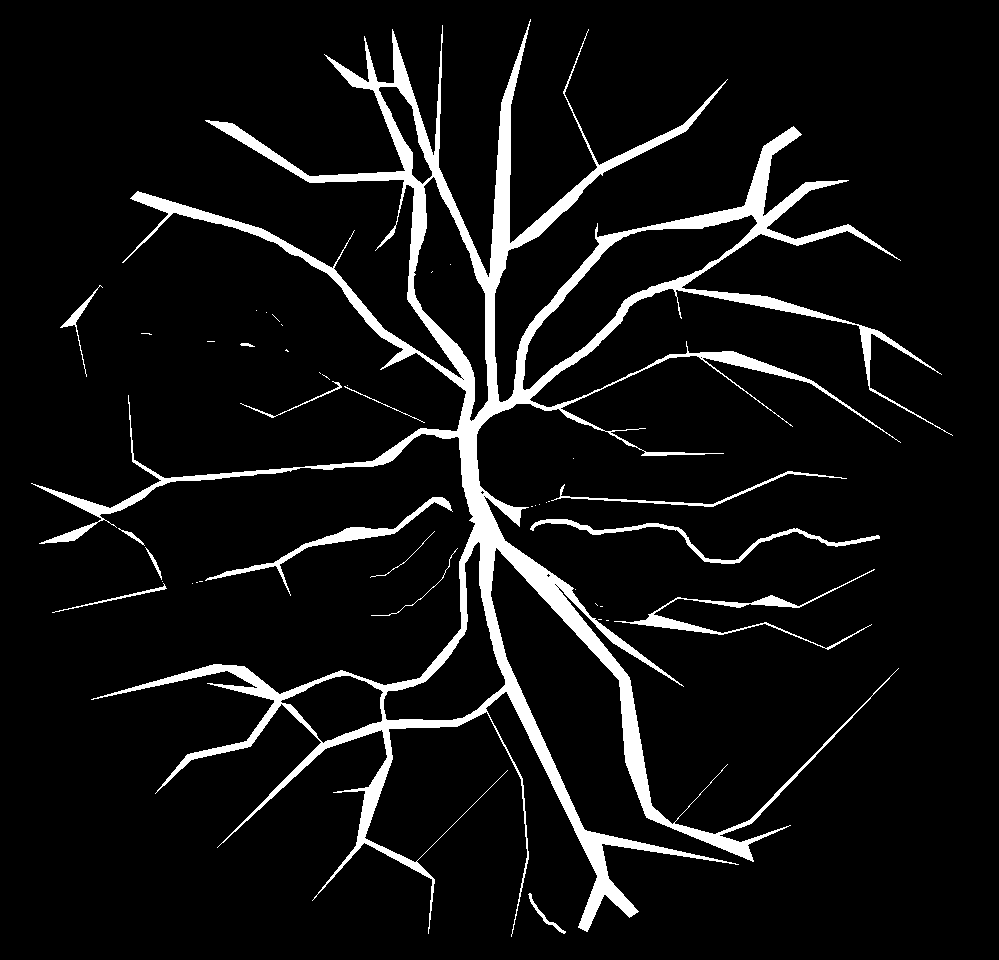

Supplement: Supplementary file 1 — Additional file 1. Generated noisy label maps. [file 12880_2021_732_MOESM1_ESM.zip › Noisy_label_maps/CHASE/LV3/Image_07L_1stHO.png]

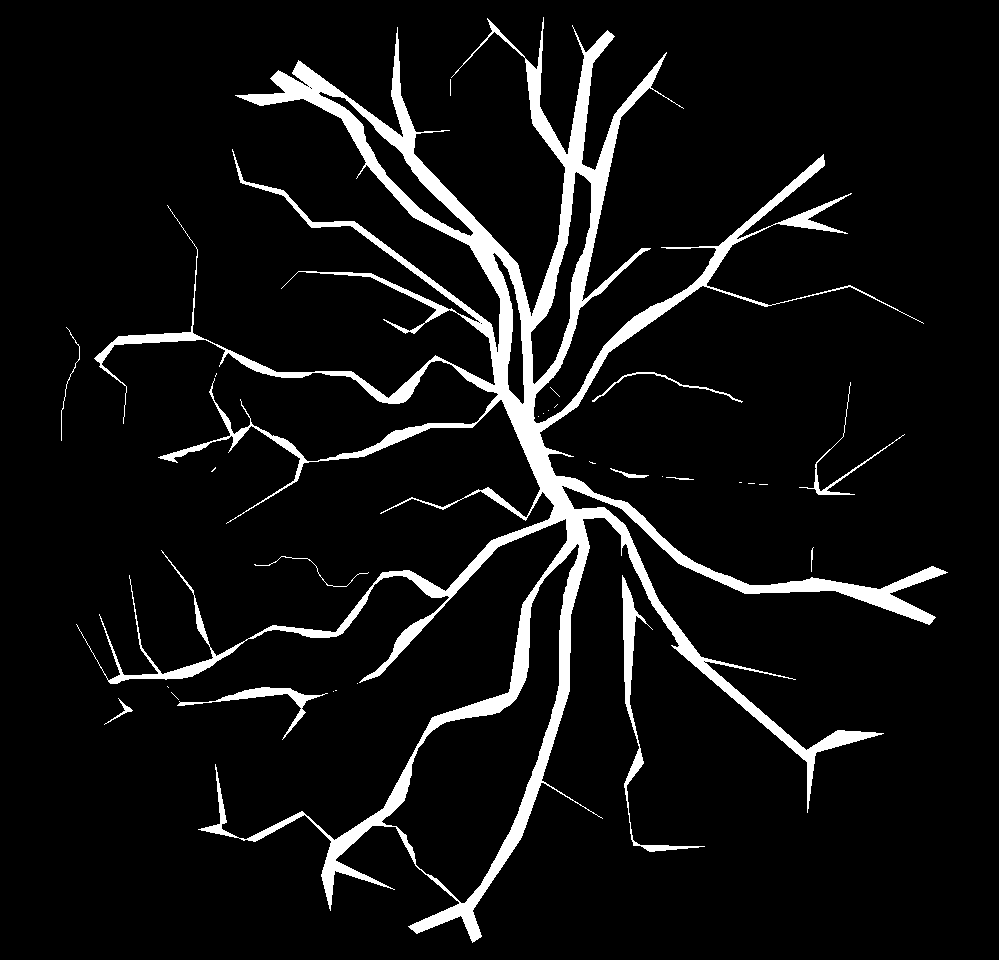

Supplement: Supplementary file 1 — Additional file 1. Generated noisy label maps. [file 12880_2021_732_MOESM1_ESM.zip › Noisy_label_maps/CHASE/LV3/Image_04R_1stHO.png]

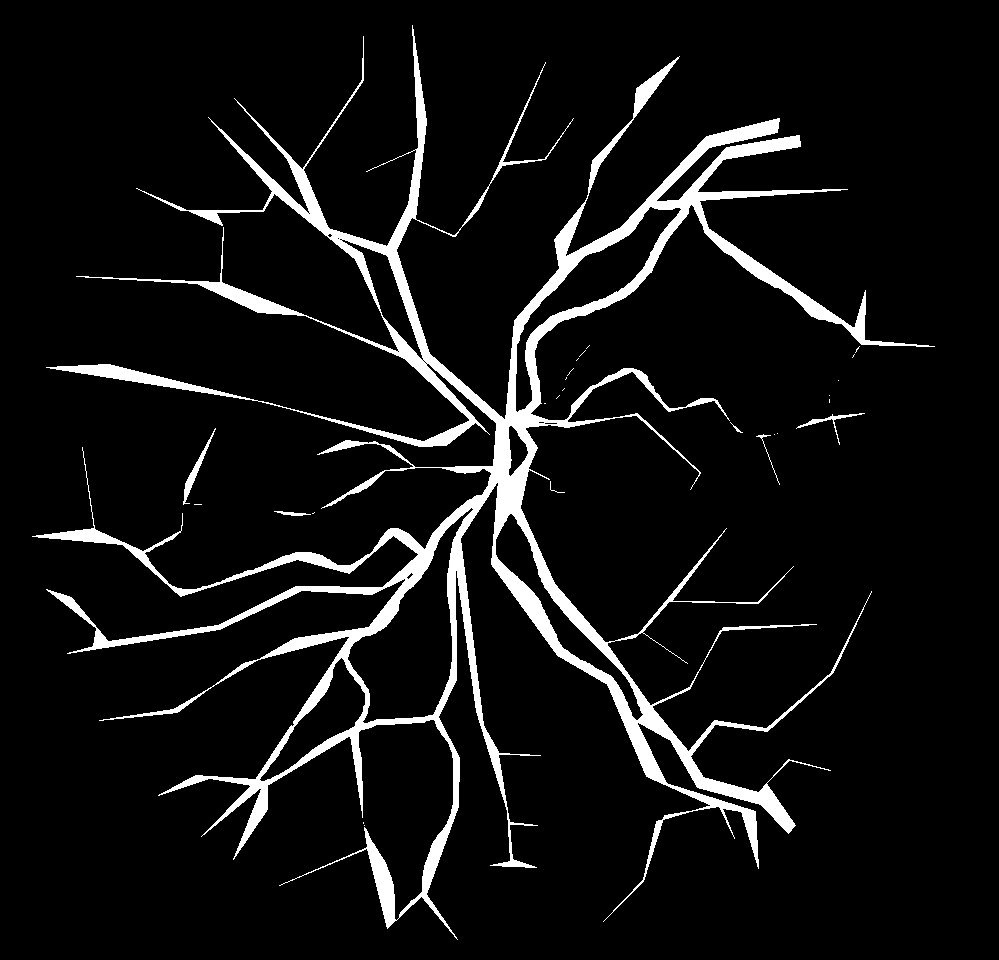

Supplement: Supplementary file 1 — Additional file 1. Generated noisy label maps. [file 12880_2021_732_MOESM1_ESM.zip › Noisy_label_maps/CHASE/LV3/Image_06L_1stHO.png]

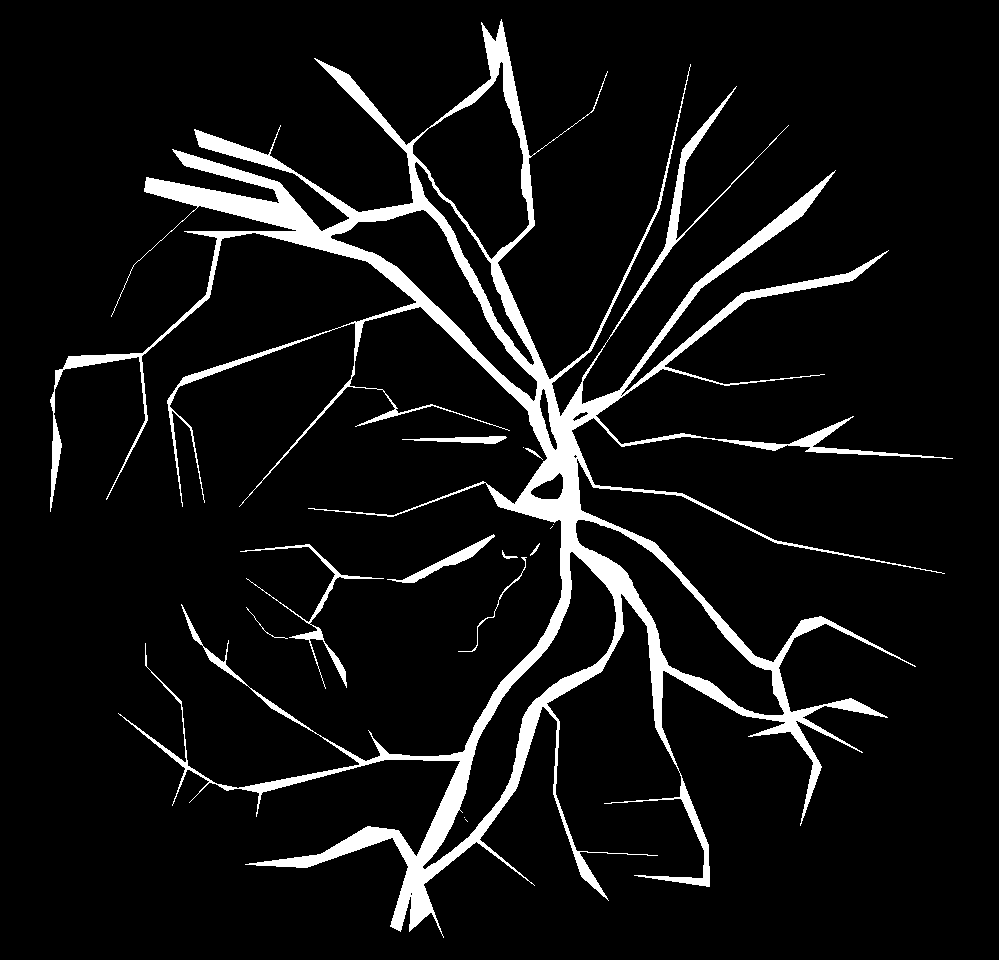

Supplement: Supplementary file 1 — Additional file 1. Generated noisy label maps. [file 12880_2021_732_MOESM1_ESM.zip › Noisy_label_maps/CHASE/LV3/Image_03R_1stHO.png]

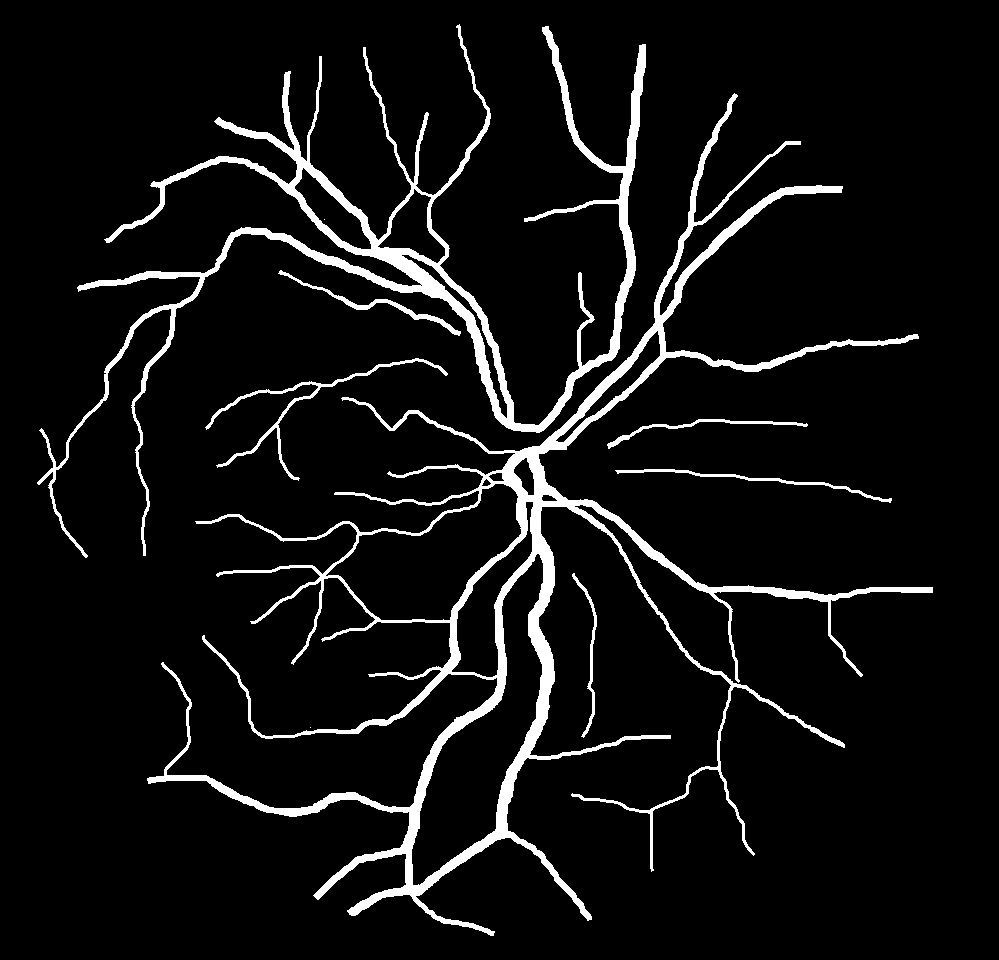

Supplement: Supplementary file 1 — Additional file 1. Generated noisy label maps. [file 12880_2021_732_MOESM1_ESM.zip › Noisy_label_maps/CHASE/Manual/Image_06R_1stHO.png]

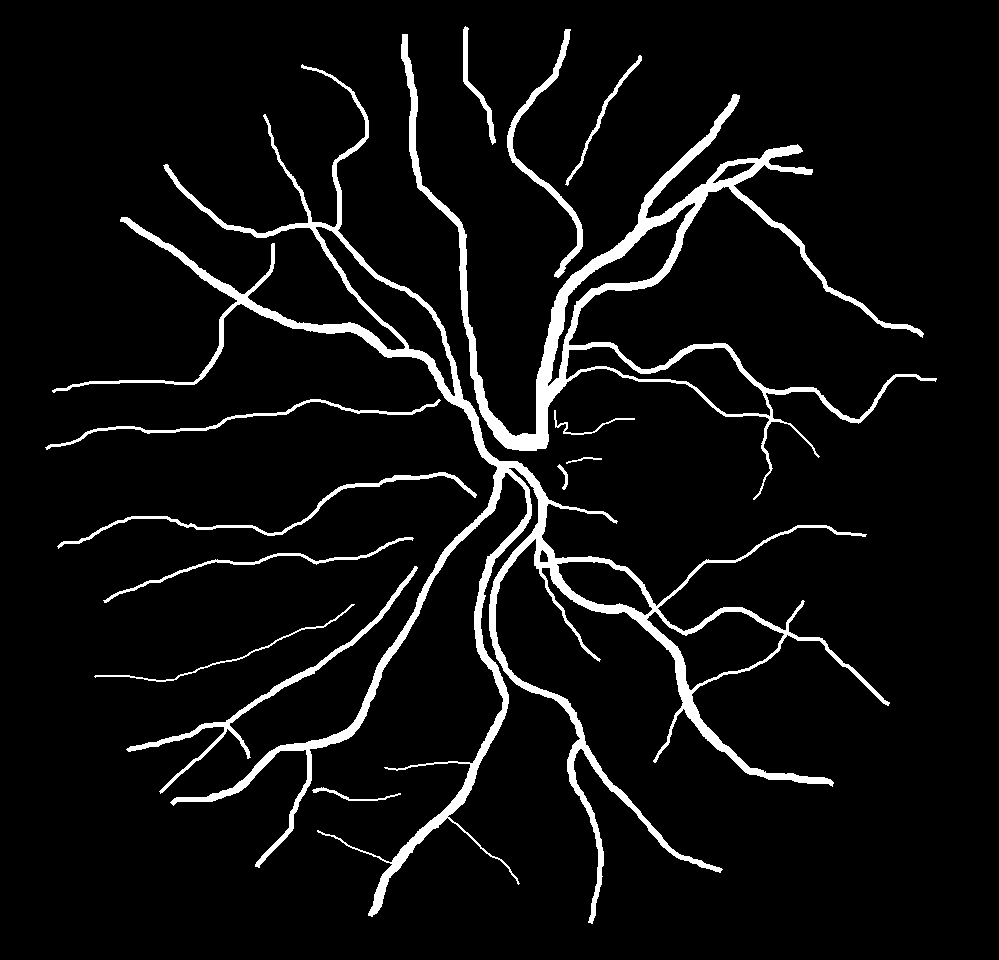

Supplement: Supplementary file 1 — Additional file 1. Generated noisy label maps. [file 12880_2021_732_MOESM1_ESM.zip › Noisy_label_maps/CHASE/Manual/Image_05L_1stHO.png]

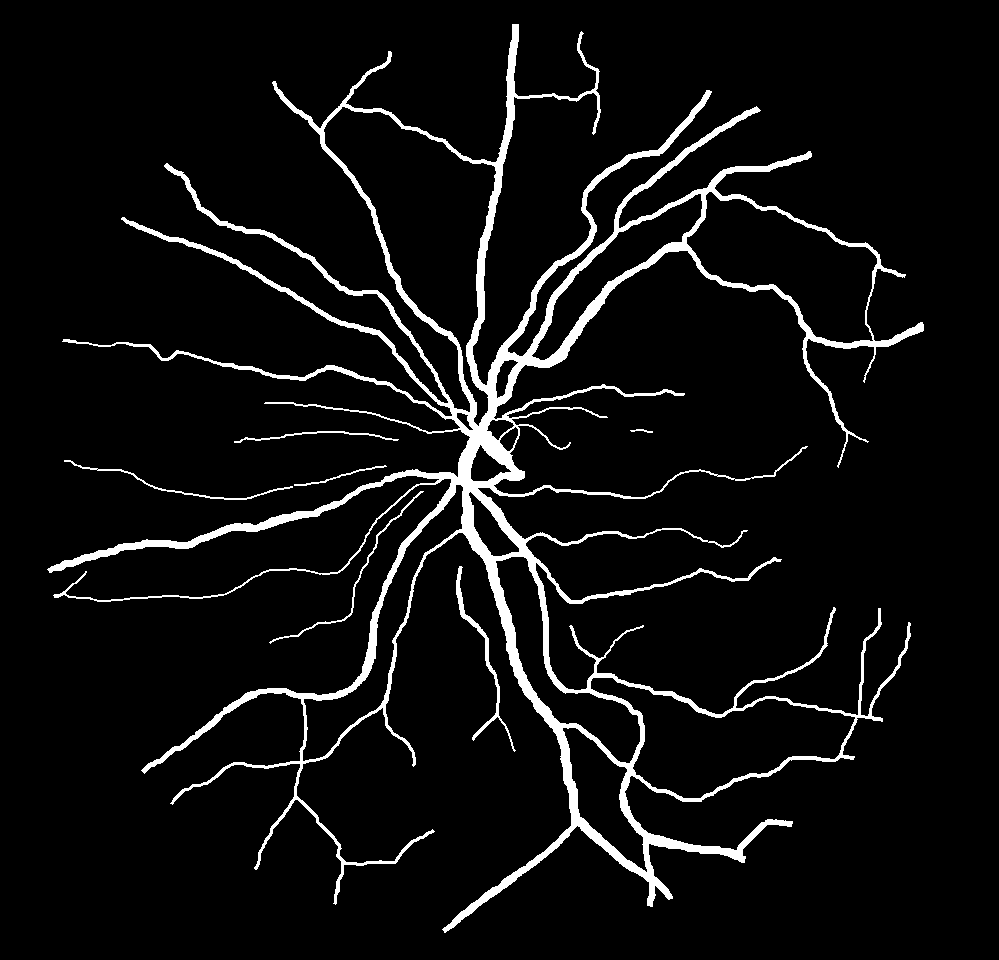

Supplement: Supplementary file 1 — Additional file 1. Generated noisy label maps. [file 12880_2021_732_MOESM1_ESM.zip › Noisy_label_maps/CHASE/Manual/Image_03L_1stHO.png]

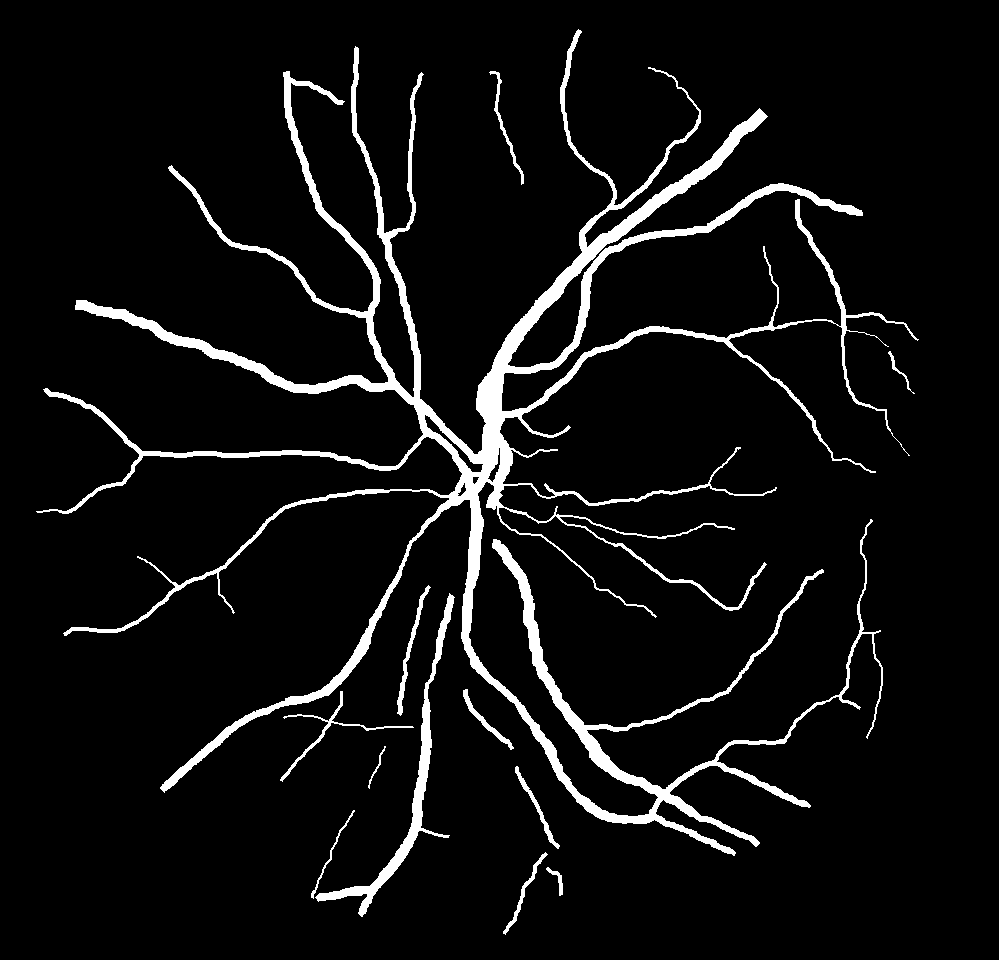

Supplement: Supplementary file 1 — Additional file 1. Generated noisy label maps. [file 12880_2021_732_MOESM1_ESM.zip › Noisy_label_maps/CHASE/Manual/Image_02L_1stHO.png]

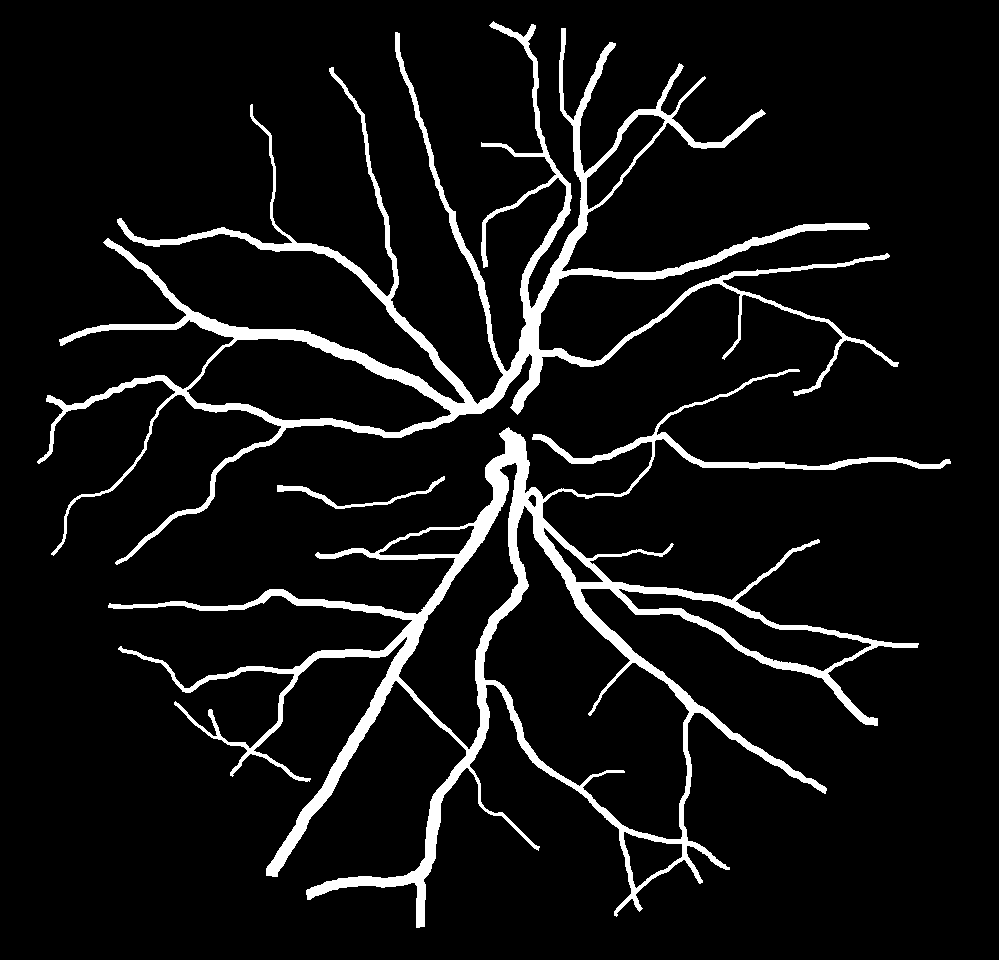

Supplement: Supplementary file 1 — Additional file 1. Generated noisy label maps. [file 12880_2021_732_MOESM1_ESM.zip › Noisy_label_maps/CHASE/Manual/Image_07R_1stHO.png]

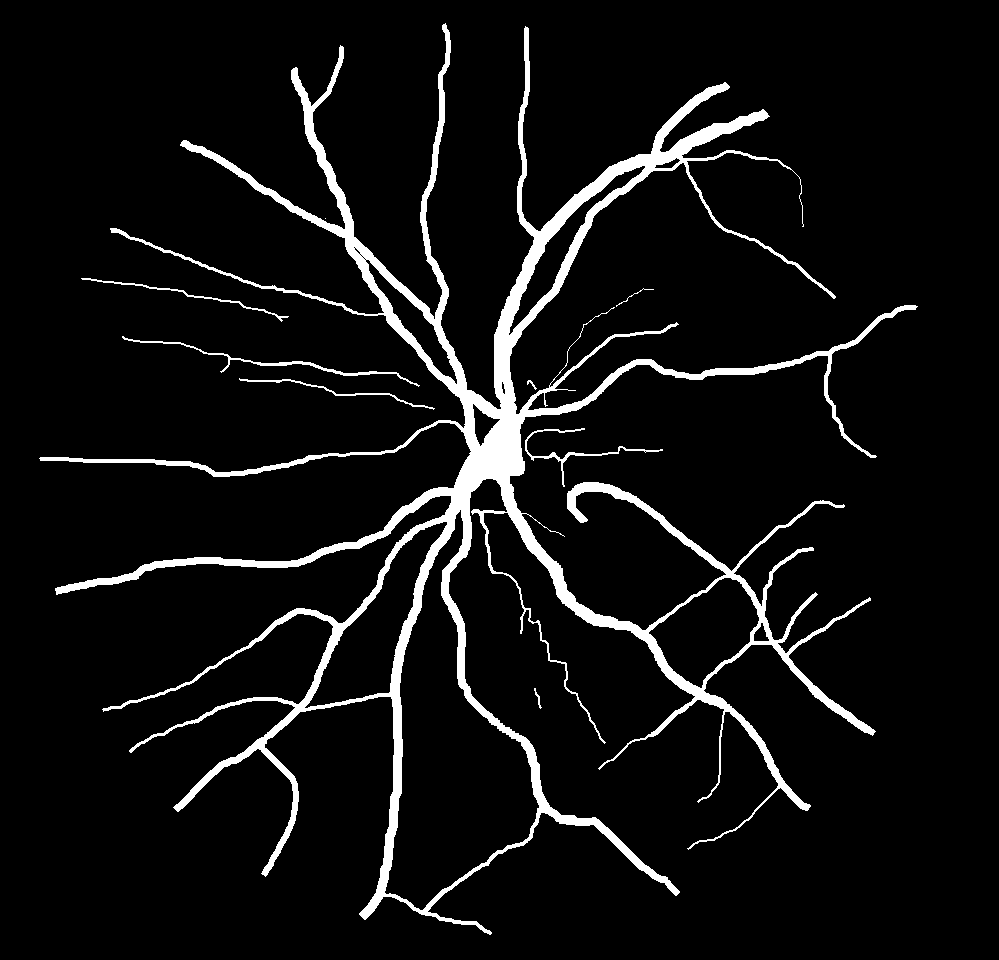

Supplement: Supplementary file 1 — Additional file 1. Generated noisy label maps. [file 12880_2021_732_MOESM1_ESM.zip › Noisy_label_maps/CHASE/Manual/Image_01L_1stHO.png]

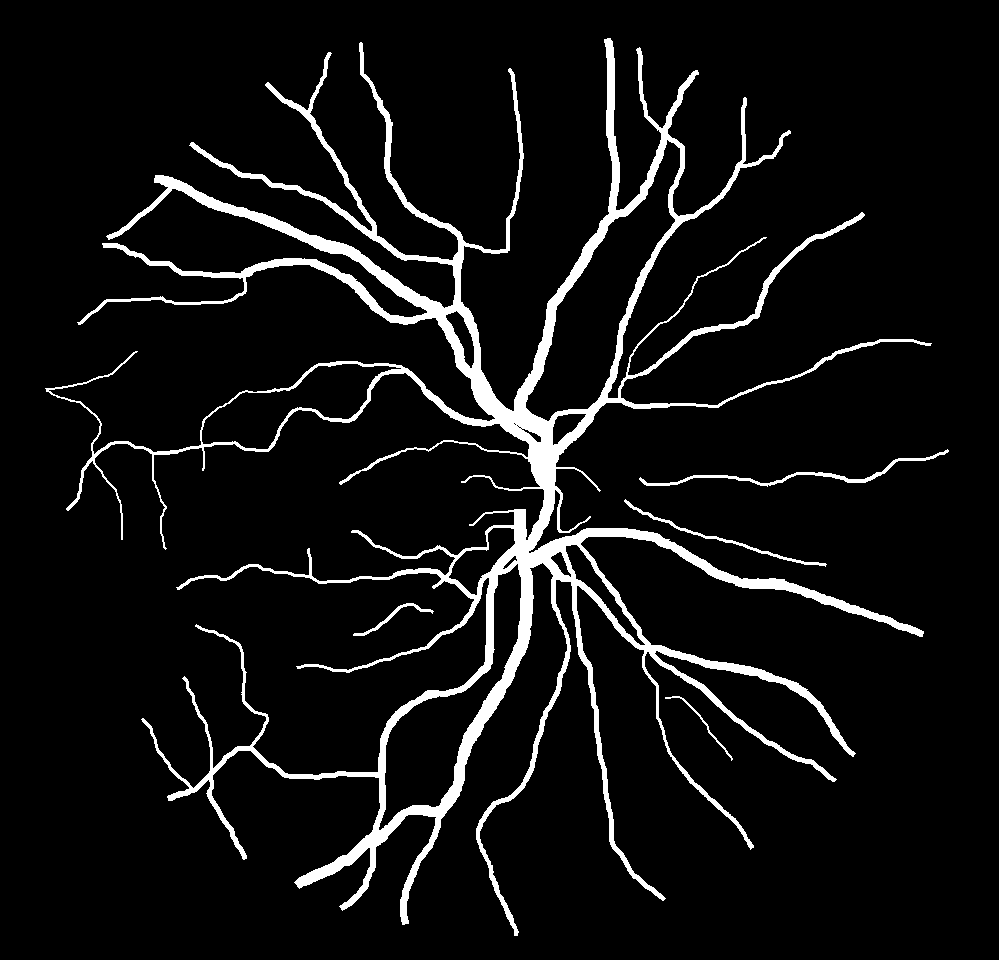

Supplement: Supplementary file 1 — Additional file 1. Generated noisy label maps. [file 12880_2021_732_MOESM1_ESM.zip › Noisy_label_maps/CHASE/Manual/Image_05R_1stHO.png]

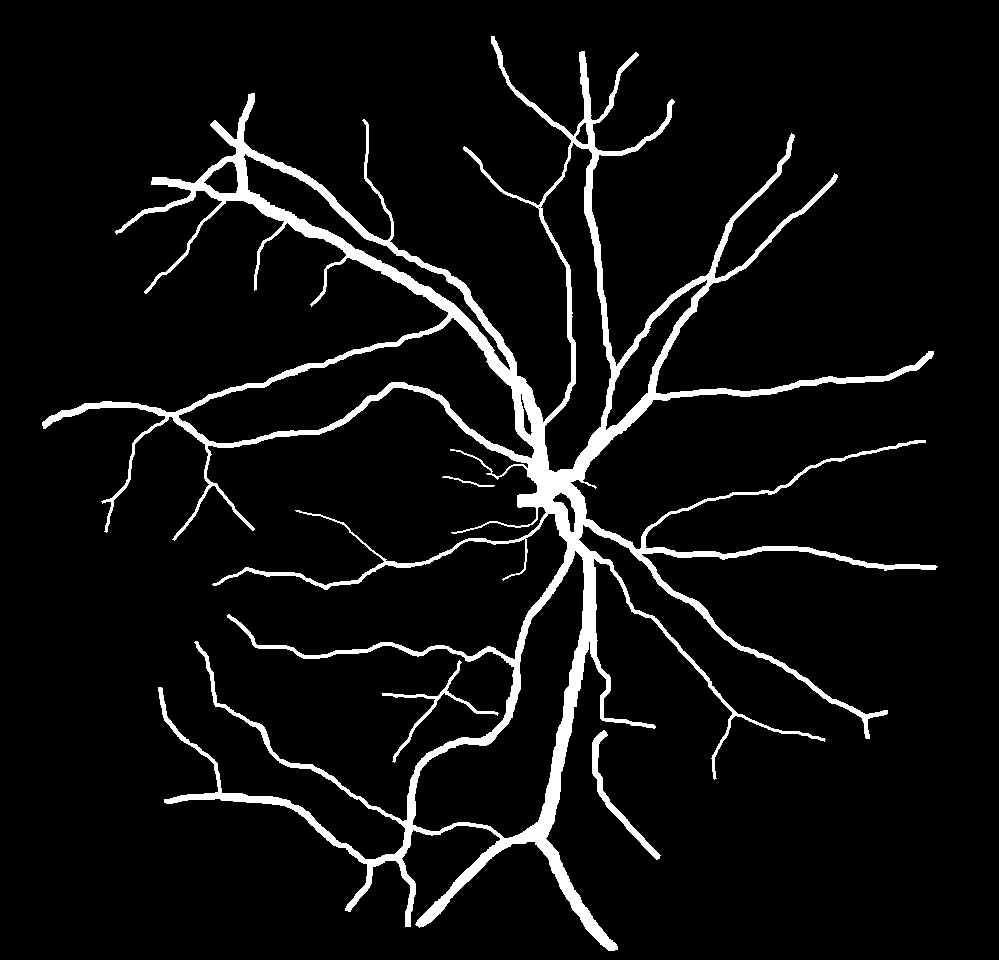

Supplement: Supplementary file 1 — Additional file 1. Generated noisy label maps. [file 12880_2021_732_MOESM1_ESM.zip › Noisy_label_maps/CHASE/Manual/Image_01R_1stHO.png]

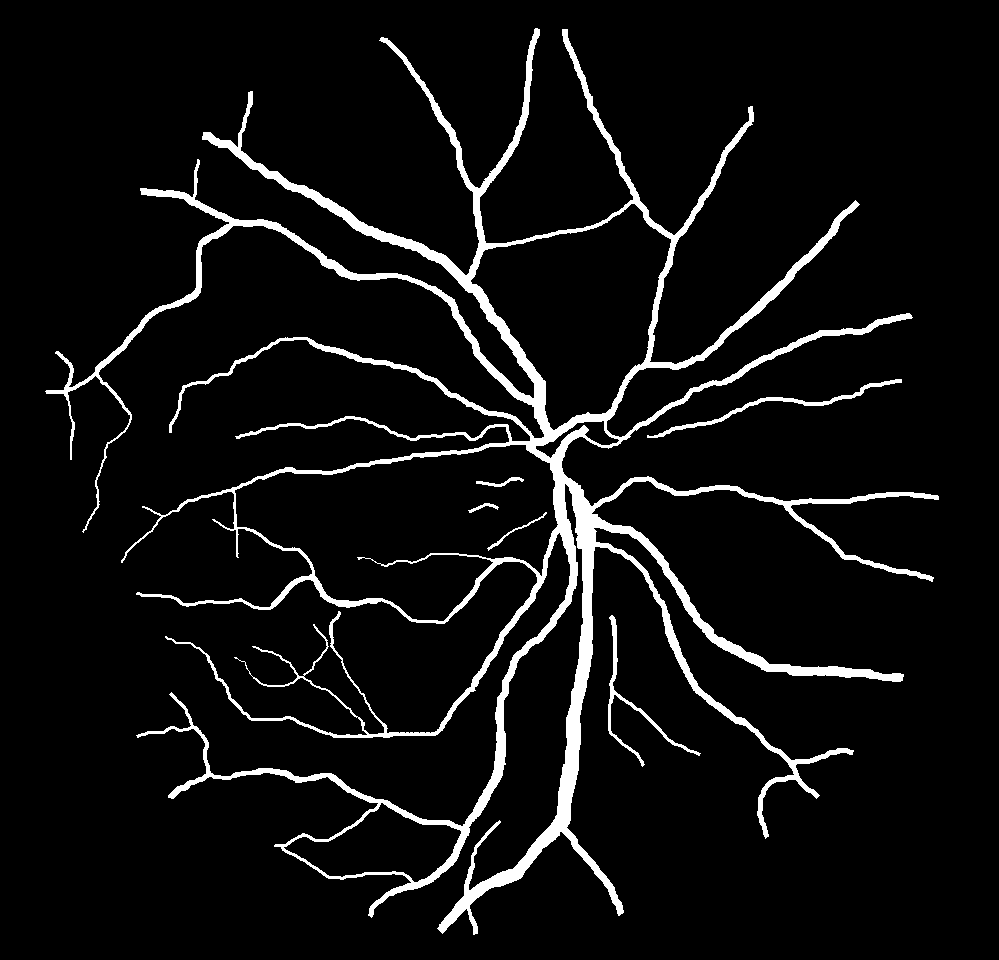

Supplement: Supplementary file 1 — Additional file 1. Generated noisy label maps. [file 12880_2021_732_MOESM1_ESM.zip › Noisy_label_maps/CHASE/Manual/Image_02R_1stHO.png]

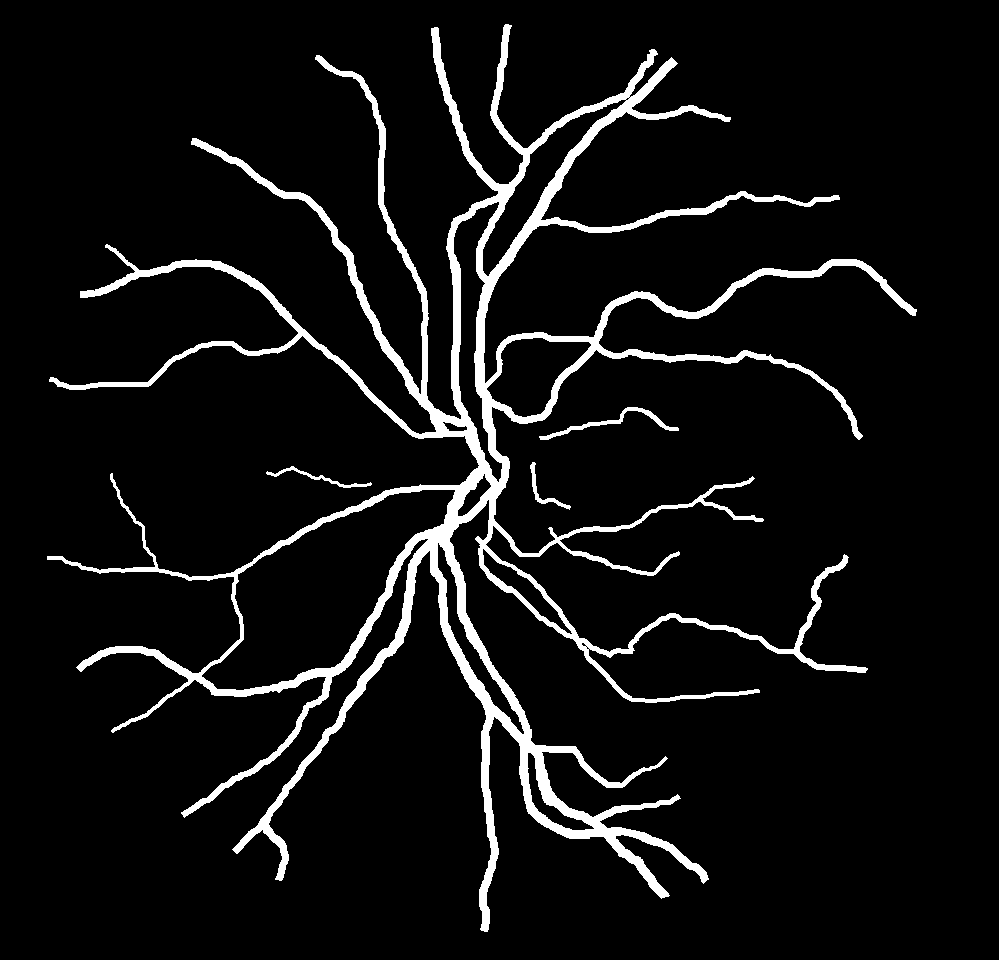

Supplement: Supplementary file 1 — Additional file 1. Generated noisy label maps. [file 12880_2021_732_MOESM1_ESM.zip › Noisy_label_maps/CHASE/Manual/Image_04L_1stHO.png]

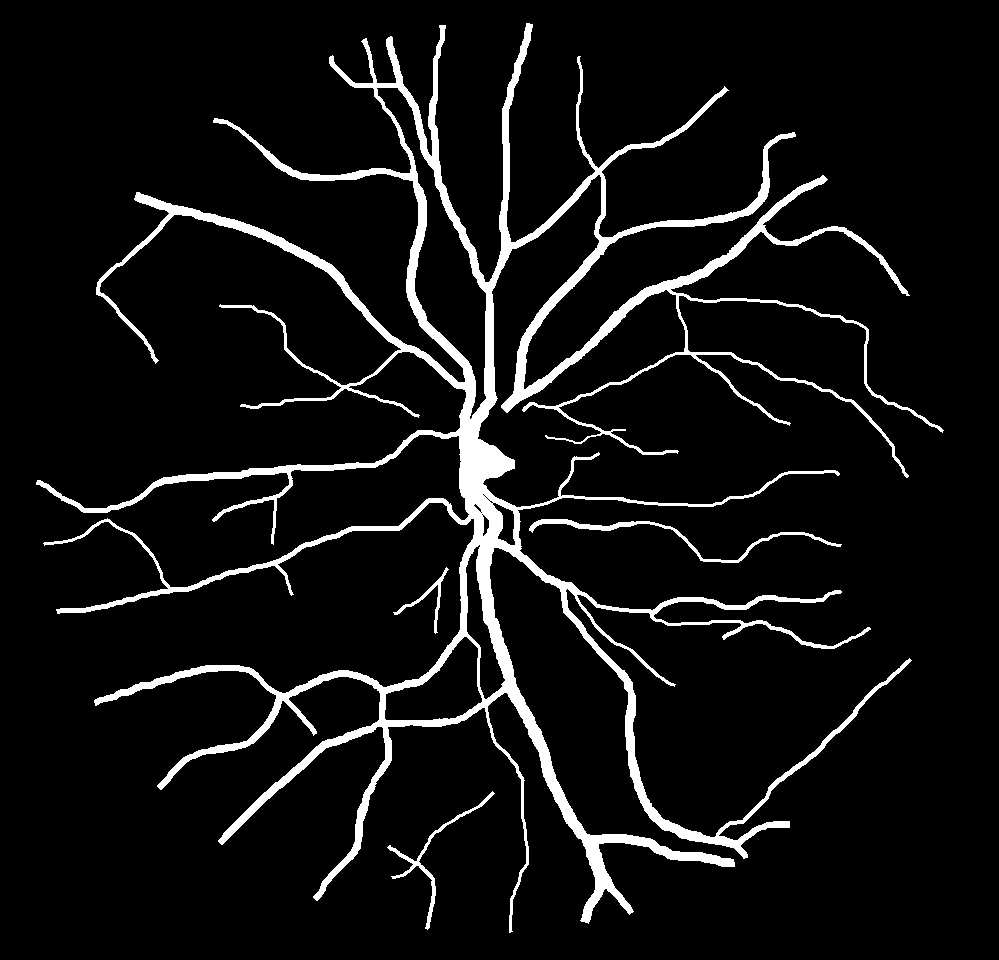

Supplement: Supplementary file 1 — Additional file 1. Generated noisy label maps. [file 12880_2021_732_MOESM1_ESM.zip › Noisy_label_maps/CHASE/Manual/Image_07L_1stHO.png]

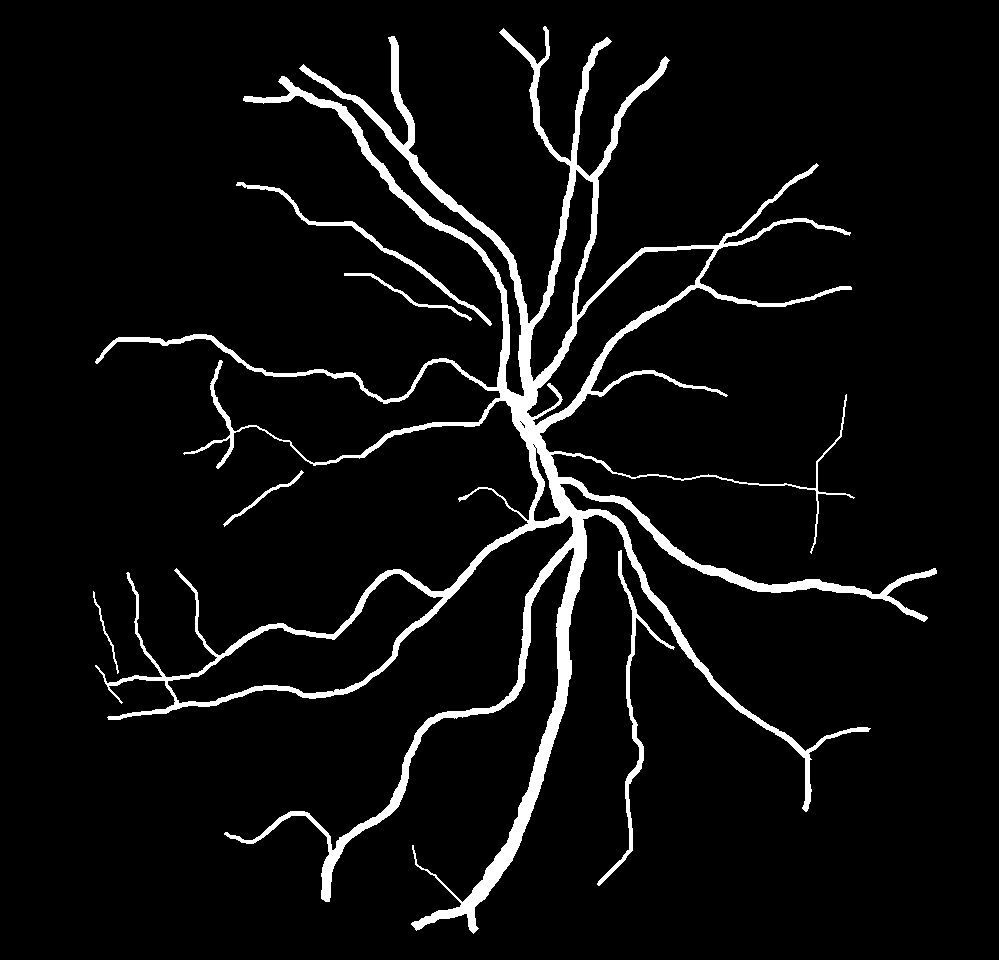

Supplement: Supplementary file 1 — Additional file 1. Generated noisy label maps. [file 12880_2021_732_MOESM1_ESM.zip › Noisy_label_maps/CHASE/Manual/Image_04R_1stHO.png]

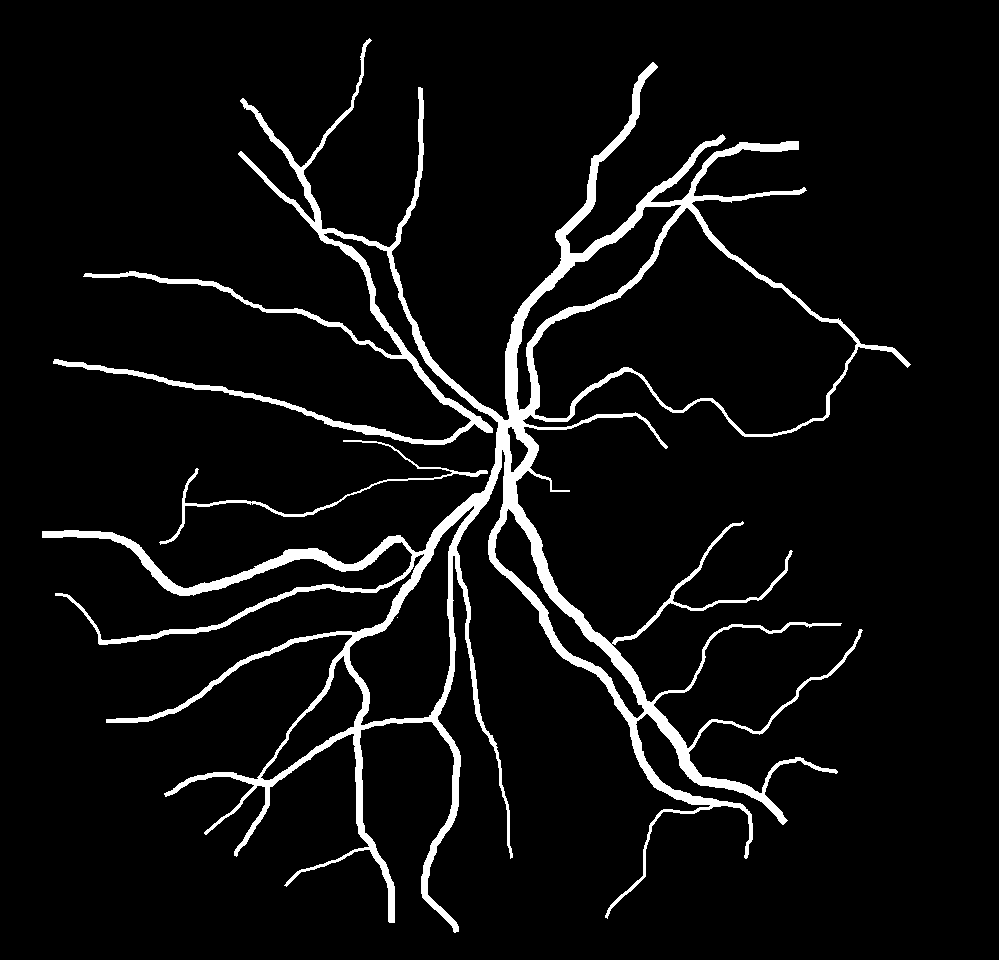

Supplement: Supplementary file 1 — Additional file 1. Generated noisy label maps. [file 12880_2021_732_MOESM1_ESM.zip › Noisy_label_maps/CHASE/Manual/Image_06L_1stHO.png]

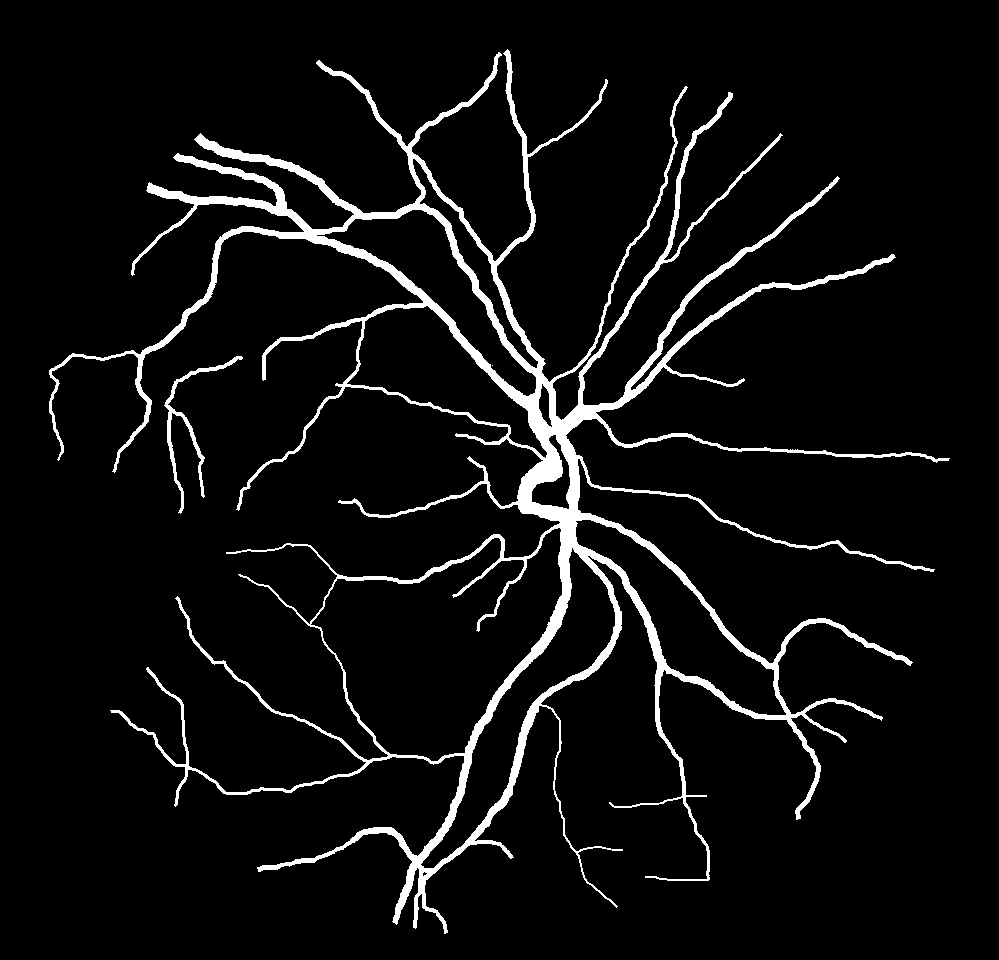

Supplement: Supplementary file 1 — Additional file 1. Generated noisy label maps. [file 12880_2021_732_MOESM1_ESM.zip › Noisy_label_maps/CHASE/Manual/Image_03R_1stHO.png]
