# Supplementary figures and images for: Self-relabeling for noise-tolerant retina vessel segmentation through label reliability estimation (part 1 of 2)
Source: BMC Med Imaging. 2022 Jan 12;22:8. doi: 10.1186/s12880-021-00732-y (PMC8753937; doi:10.1186/s12880-021-00732-y)

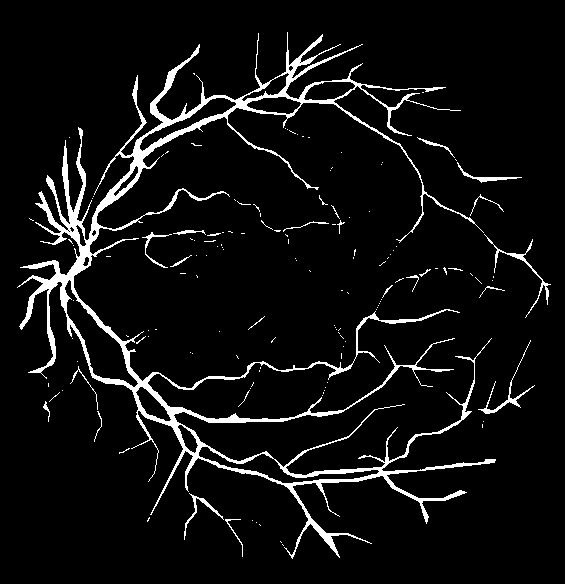

Supplement: Supplementary file 1 — Additional file 1. Generated noisy label maps. [file 12880_2021_732_MOESM1_ESM.zip › Noisy_label_maps/DRIVE(R)/LV1/01_manual1.png]

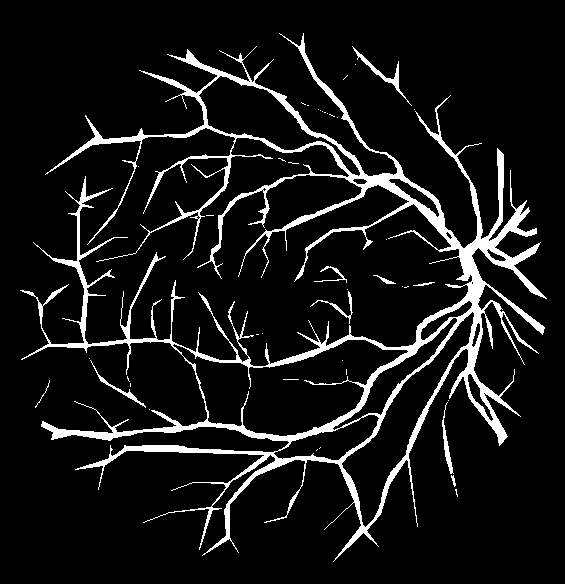

Supplement: Supplementary file 1 — Additional file 1. Generated noisy label maps. [file 12880_2021_732_MOESM1_ESM.zip › Noisy_label_maps/DRIVE(R)/LV1/02_manual1.png]

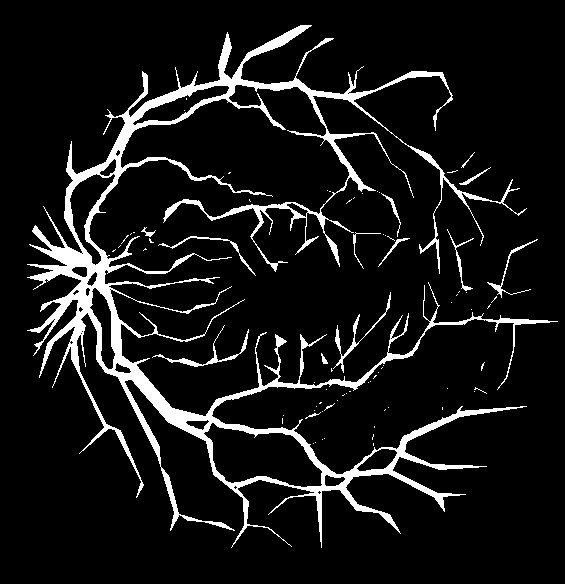

Supplement: Supplementary file 1 — Additional file 1. Generated noisy label maps. [file 12880_2021_732_MOESM1_ESM.zip › Noisy_label_maps/DRIVE(R)/LV1/03_manual1.png]

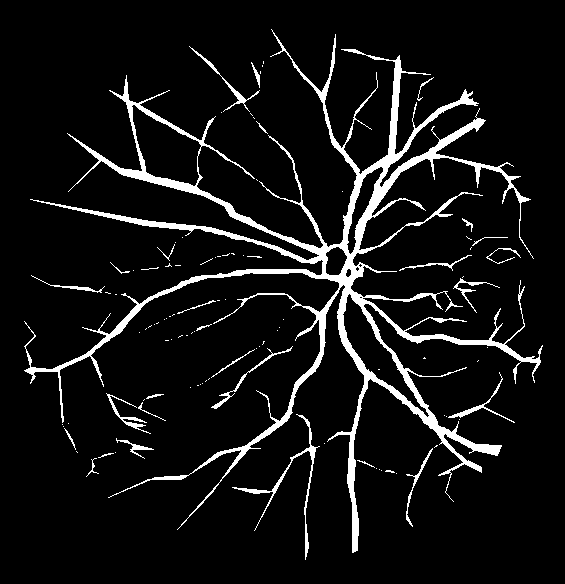

Supplement: Supplementary file 1 — Additional file 1. Generated noisy label maps. [file 12880_2021_732_MOESM1_ESM.zip › Noisy_label_maps/DRIVE(R)/LV1/04_manual1.png]

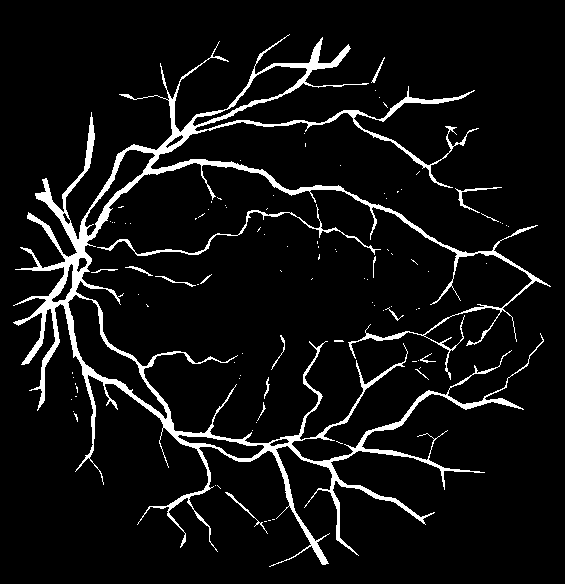

Supplement: Supplementary file 1 — Additional file 1. Generated noisy label maps. [file 12880_2021_732_MOESM1_ESM.zip › Noisy_label_maps/DRIVE(R)/LV1/05_manual1.png]

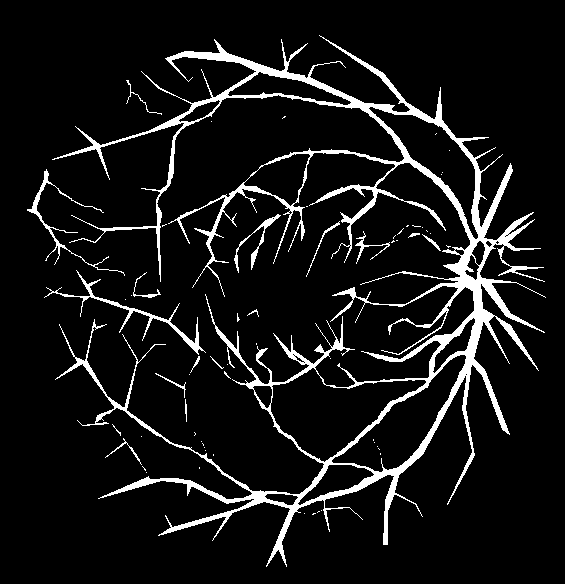

Supplement: Supplementary file 1 — Additional file 1. Generated noisy label maps. [file 12880_2021_732_MOESM1_ESM.zip › Noisy_label_maps/DRIVE(R)/LV1/06_manual1.png]

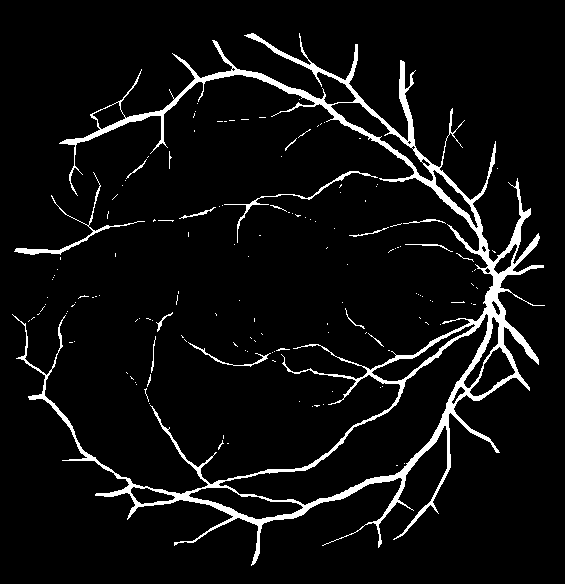

Supplement: Supplementary file 1 — Additional file 1. Generated noisy label maps. [file 12880_2021_732_MOESM1_ESM.zip › Noisy_label_maps/DRIVE(R)/LV1/07_manual1.png]

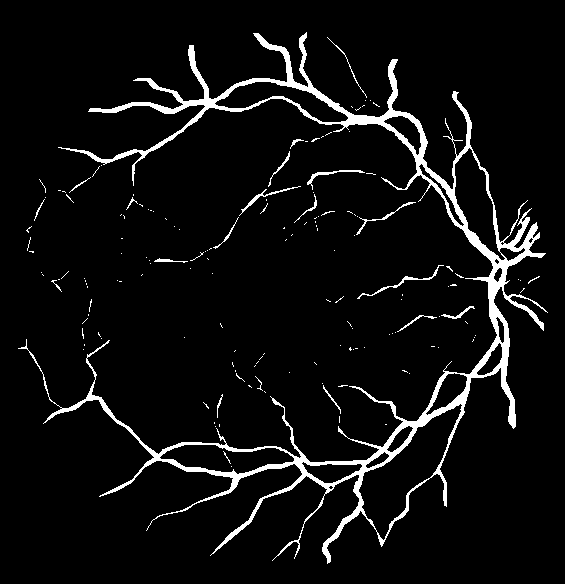

Supplement: Supplementary file 1 — Additional file 1. Generated noisy label maps. [file 12880_2021_732_MOESM1_ESM.zip › Noisy_label_maps/DRIVE(R)/LV1/08_manual1.png]

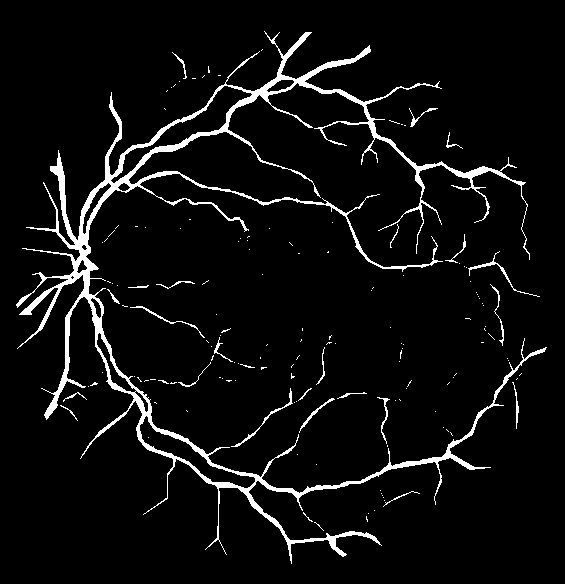

Supplement: Supplementary file 1 — Additional file 1. Generated noisy label maps. [file 12880_2021_732_MOESM1_ESM.zip › Noisy_label_maps/DRIVE(R)/LV1/09_manual1.png]

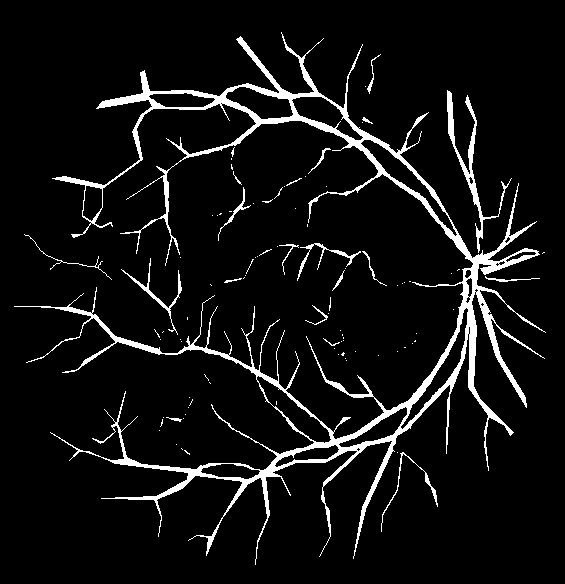

Supplement: Supplementary file 1 — Additional file 1. Generated noisy label maps. [file 12880_2021_732_MOESM1_ESM.zip › Noisy_label_maps/DRIVE(R)/LV1/10_manual1.png]

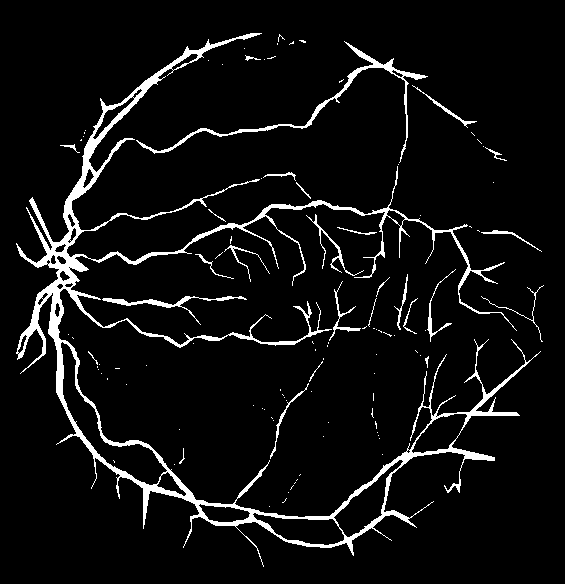

Supplement: Supplementary file 1 — Additional file 1. Generated noisy label maps. [file 12880_2021_732_MOESM1_ESM.zip › Noisy_label_maps/DRIVE(R)/LV1/11_manual1.png]

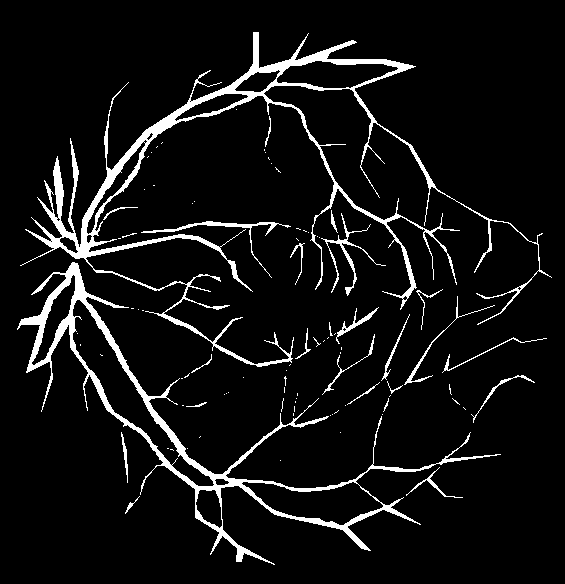

Supplement: Supplementary file 1 — Additional file 1. Generated noisy label maps. [file 12880_2021_732_MOESM1_ESM.zip › Noisy_label_maps/DRIVE(R)/LV1/12_manual1.png]

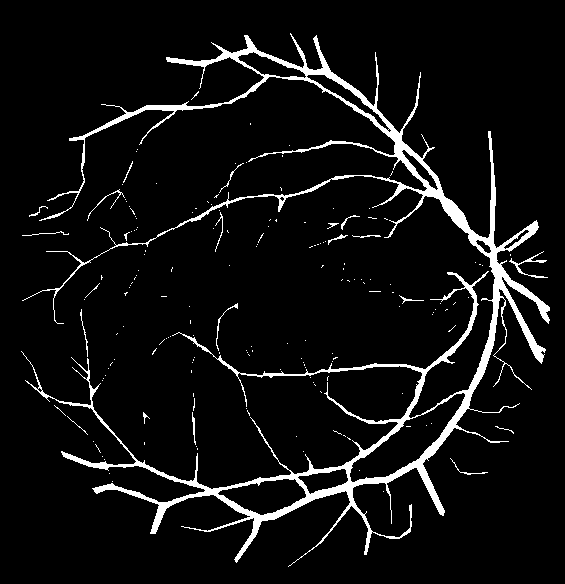

Supplement: Supplementary file 1 — Additional file 1. Generated noisy label maps. [file 12880_2021_732_MOESM1_ESM.zip › Noisy_label_maps/DRIVE(R)/LV1/13_manual1.png]

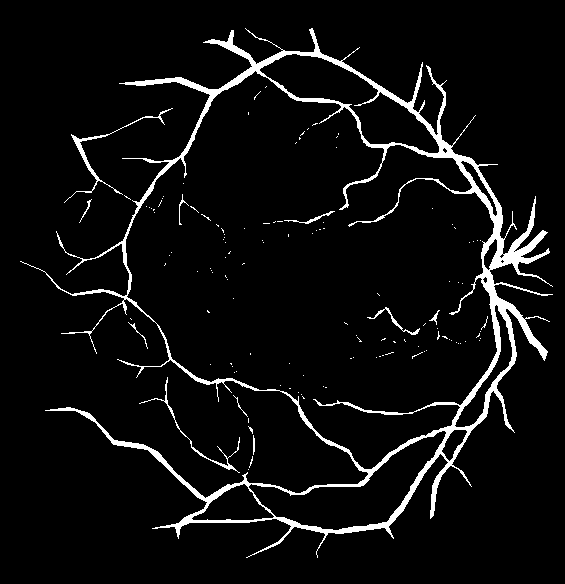

Supplement: Supplementary file 1 — Additional file 1. Generated noisy label maps. [file 12880_2021_732_MOESM1_ESM.zip › Noisy_label_maps/DRIVE(R)/LV1/14_manual1.png]

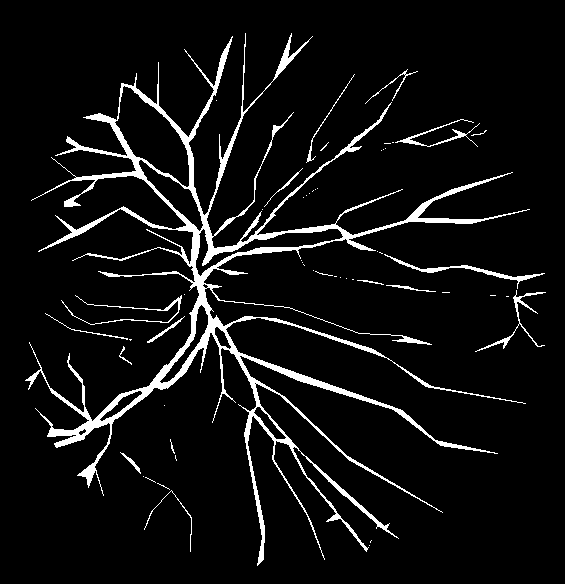

Supplement: Supplementary file 1 — Additional file 1. Generated noisy label maps. [file 12880_2021_732_MOESM1_ESM.zip › Noisy_label_maps/DRIVE(R)/LV1/15_manual1.png]

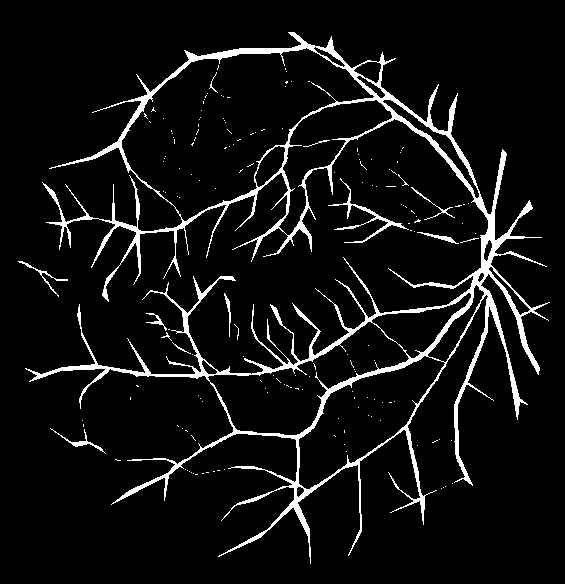

Supplement: Supplementary file 1 — Additional file 1. Generated noisy label maps. [file 12880_2021_732_MOESM1_ESM.zip › Noisy_label_maps/DRIVE(R)/LV1/16_manual1.png]

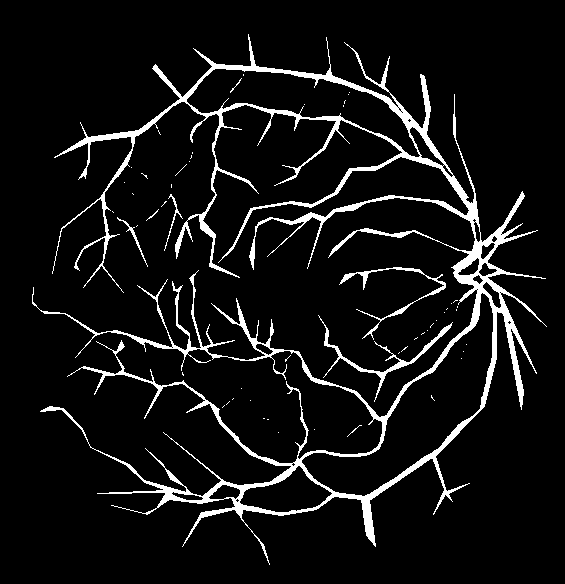

Supplement: Supplementary file 1 — Additional file 1. Generated noisy label maps. [file 12880_2021_732_MOESM1_ESM.zip › Noisy_label_maps/DRIVE(R)/LV1/17_manual1.png]

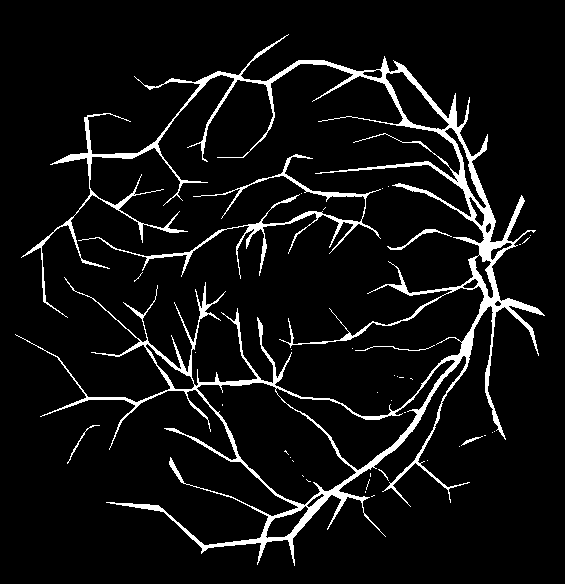

Supplement: Supplementary file 1 — Additional file 1. Generated noisy label maps. [file 12880_2021_732_MOESM1_ESM.zip › Noisy_label_maps/DRIVE(R)/LV1/18_manual1.png]

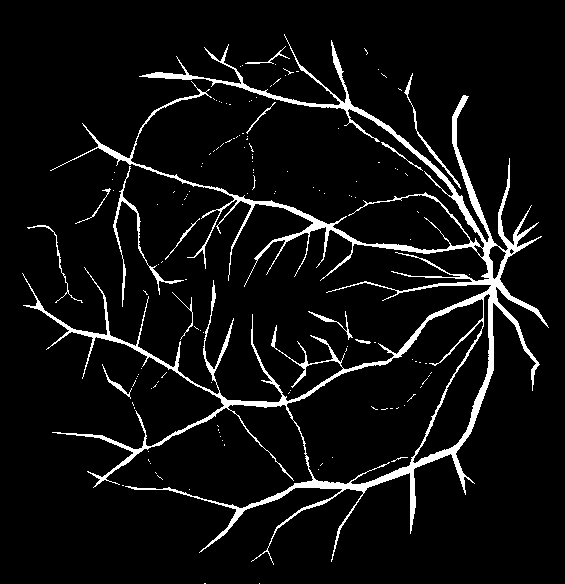

Supplement: Supplementary file 1 — Additional file 1. Generated noisy label maps. [file 12880_2021_732_MOESM1_ESM.zip › Noisy_label_maps/DRIVE(R)/LV1/19_manual1.png]

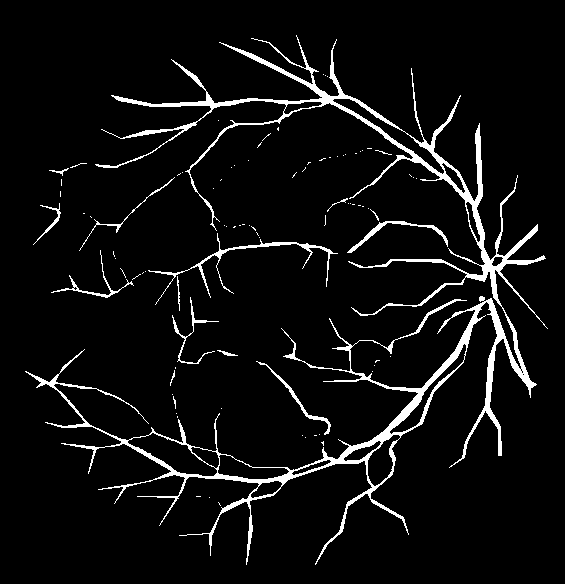

Supplement: Supplementary file 1 — Additional file 1. Generated noisy label maps. [file 12880_2021_732_MOESM1_ESM.zip › Noisy_label_maps/DRIVE(R)/LV1/20_manual1.png]

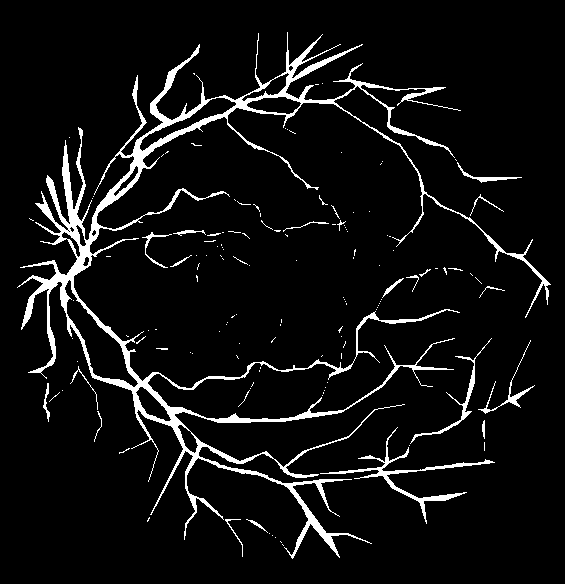

Supplement: Supplementary file 1 — Additional file 1. Generated noisy label maps. [file 12880_2021_732_MOESM1_ESM.zip › Noisy_label_maps/DRIVE(R)/LV2/01_manual1.png]

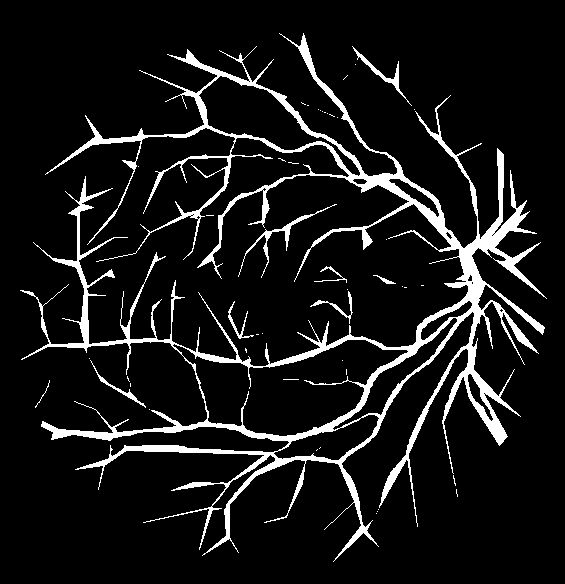

Supplement: Supplementary file 1 — Additional file 1. Generated noisy label maps. [file 12880_2021_732_MOESM1_ESM.zip › Noisy_label_maps/DRIVE(R)/LV2/02_manual1.png]

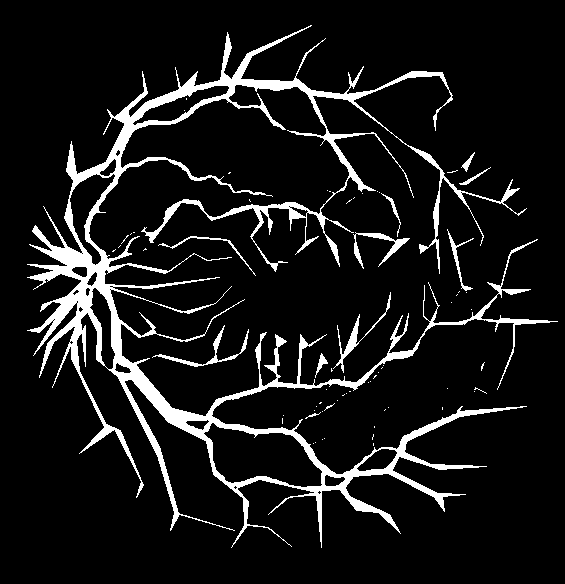

Supplement: Supplementary file 1 — Additional file 1. Generated noisy label maps. [file 12880_2021_732_MOESM1_ESM.zip › Noisy_label_maps/DRIVE(R)/LV2/03_manual1.png]

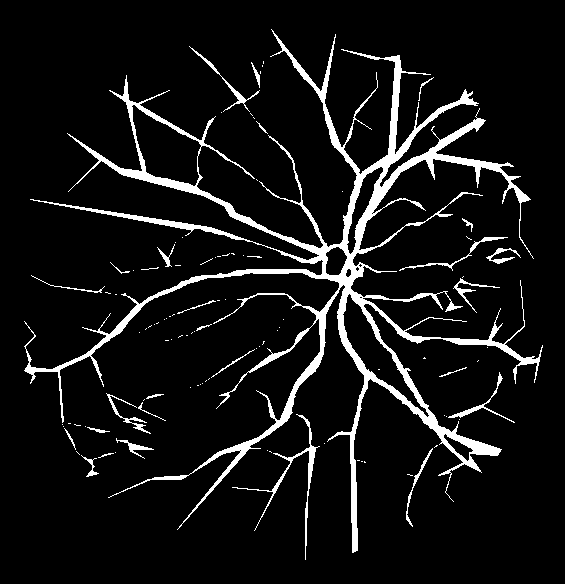

Supplement: Supplementary file 1 — Additional file 1. Generated noisy label maps. [file 12880_2021_732_MOESM1_ESM.zip › Noisy_label_maps/DRIVE(R)/LV2/04_manual1.png]

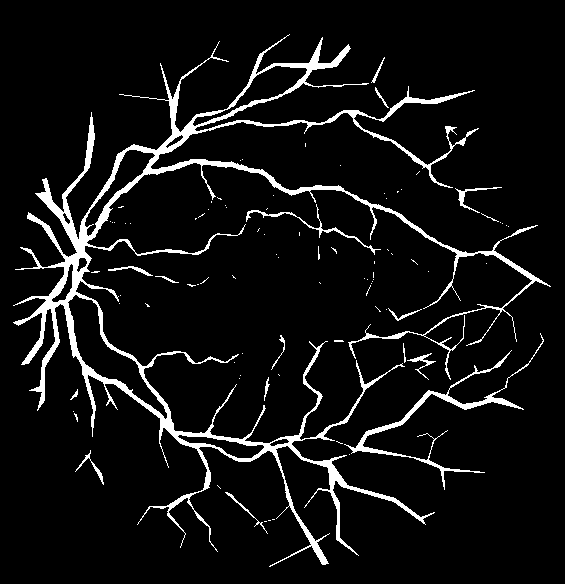

Supplement: Supplementary file 1 — Additional file 1. Generated noisy label maps. [file 12880_2021_732_MOESM1_ESM.zip › Noisy_label_maps/DRIVE(R)/LV2/05_manual1.png]

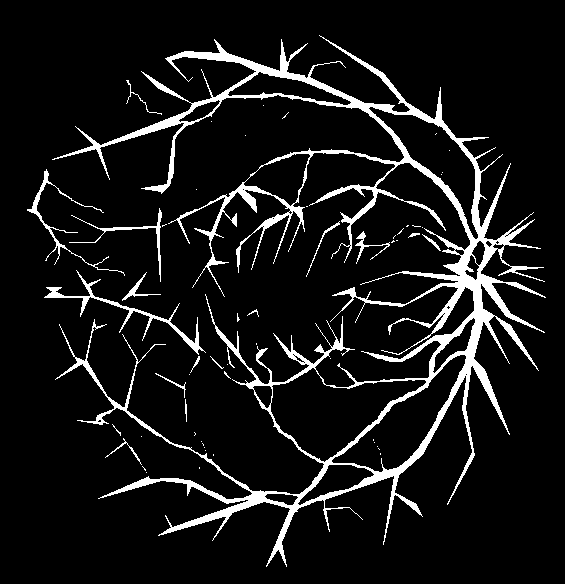

Supplement: Supplementary file 1 — Additional file 1. Generated noisy label maps. [file 12880_2021_732_MOESM1_ESM.zip › Noisy_label_maps/DRIVE(R)/LV2/06_manual1.png]

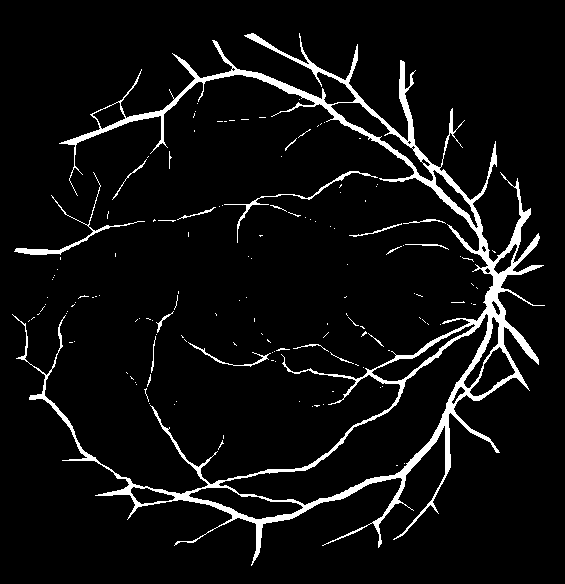

Supplement: Supplementary file 1 — Additional file 1. Generated noisy label maps. [file 12880_2021_732_MOESM1_ESM.zip › Noisy_label_maps/DRIVE(R)/LV2/07_manual1.png]

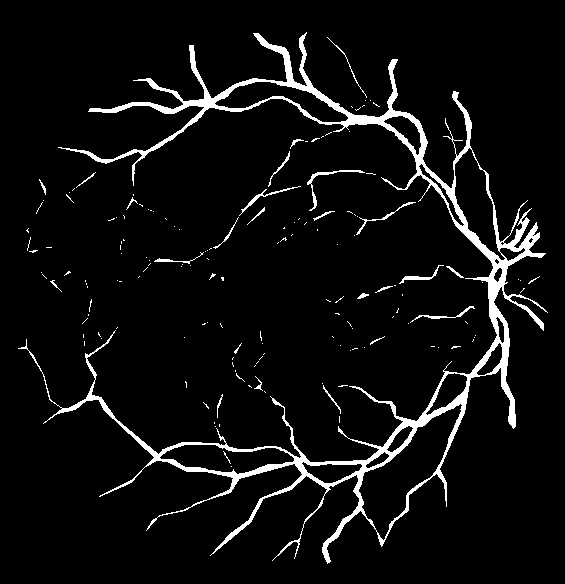

Supplement: Supplementary file 1 — Additional file 1. Generated noisy label maps. [file 12880_2021_732_MOESM1_ESM.zip › Noisy_label_maps/DRIVE(R)/LV2/08_manual1.png]

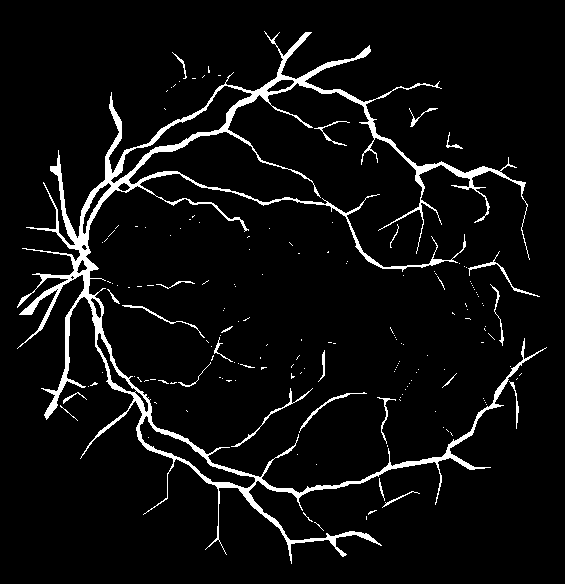

Supplement: Supplementary file 1 — Additional file 1. Generated noisy label maps. [file 12880_2021_732_MOESM1_ESM.zip › Noisy_label_maps/DRIVE(R)/LV2/09_manual1.png]

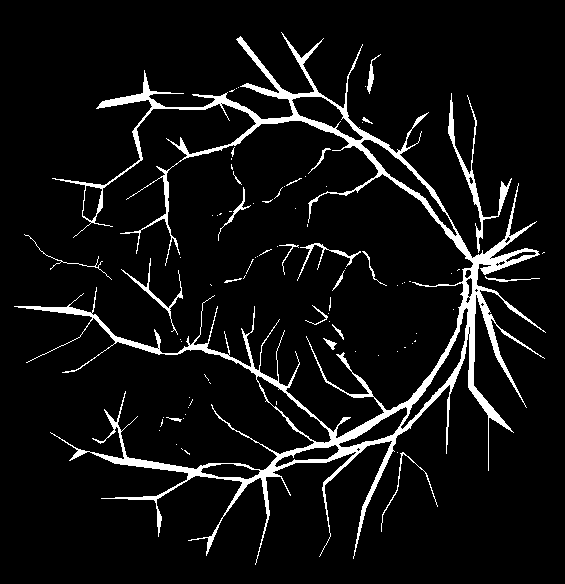

Supplement: Supplementary file 1 — Additional file 1. Generated noisy label maps. [file 12880_2021_732_MOESM1_ESM.zip › Noisy_label_maps/DRIVE(R)/LV2/10_manual1.png]

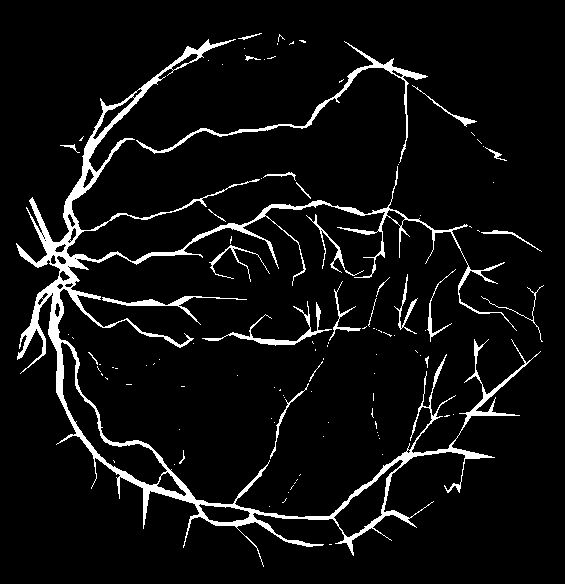

Supplement: Supplementary file 1 — Additional file 1. Generated noisy label maps. [file 12880_2021_732_MOESM1_ESM.zip › Noisy_label_maps/DRIVE(R)/LV2/11_manual1.png]

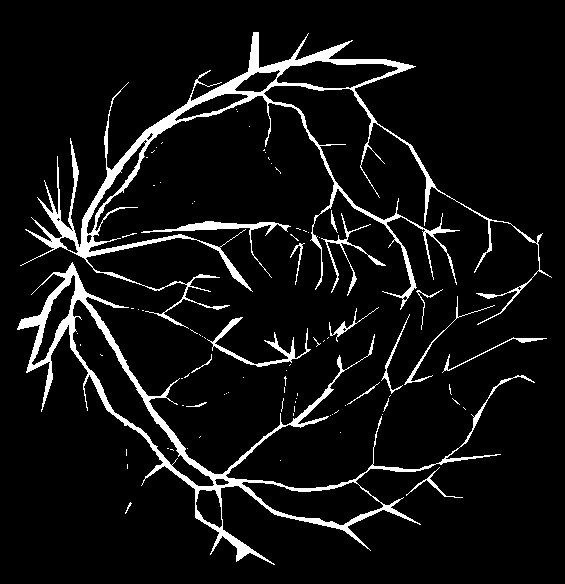

Supplement: Supplementary file 1 — Additional file 1. Generated noisy label maps. [file 12880_2021_732_MOESM1_ESM.zip › Noisy_label_maps/DRIVE(R)/LV2/12_manual1.png]

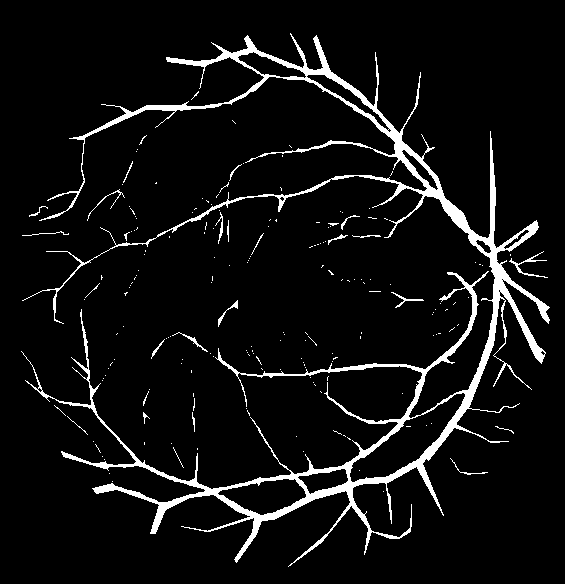

Supplement: Supplementary file 1 — Additional file 1. Generated noisy label maps. [file 12880_2021_732_MOESM1_ESM.zip › Noisy_label_maps/DRIVE(R)/LV2/13_manual1.png]

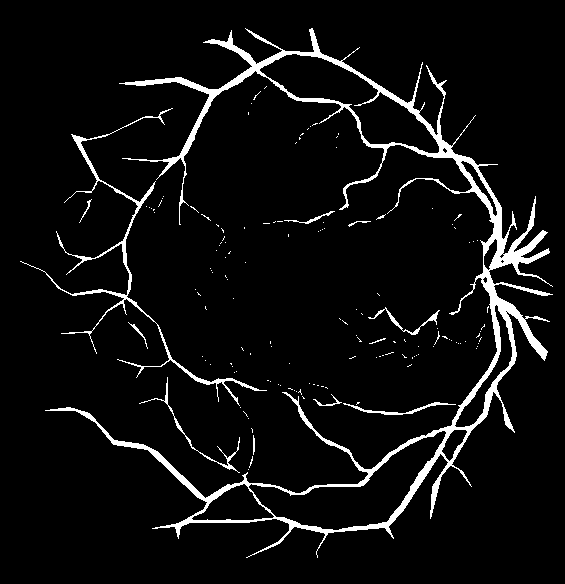

Supplement: Supplementary file 1 — Additional file 1. Generated noisy label maps. [file 12880_2021_732_MOESM1_ESM.zip › Noisy_label_maps/DRIVE(R)/LV2/14_manual1.png]

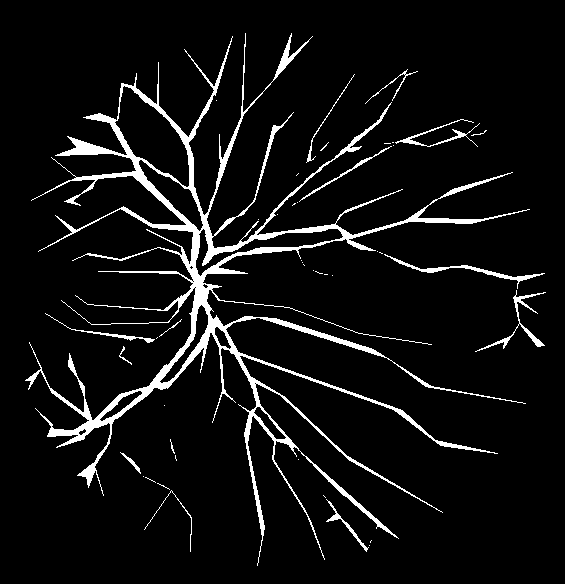

Supplement: Supplementary file 1 — Additional file 1. Generated noisy label maps. [file 12880_2021_732_MOESM1_ESM.zip › Noisy_label_maps/DRIVE(R)/LV2/15_manual1.png]

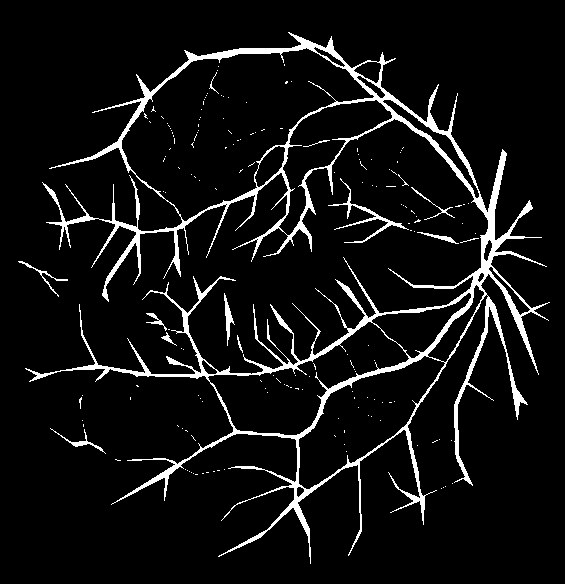

Supplement: Supplementary file 1 — Additional file 1. Generated noisy label maps. [file 12880_2021_732_MOESM1_ESM.zip › Noisy_label_maps/DRIVE(R)/LV2/16_manual1.png]

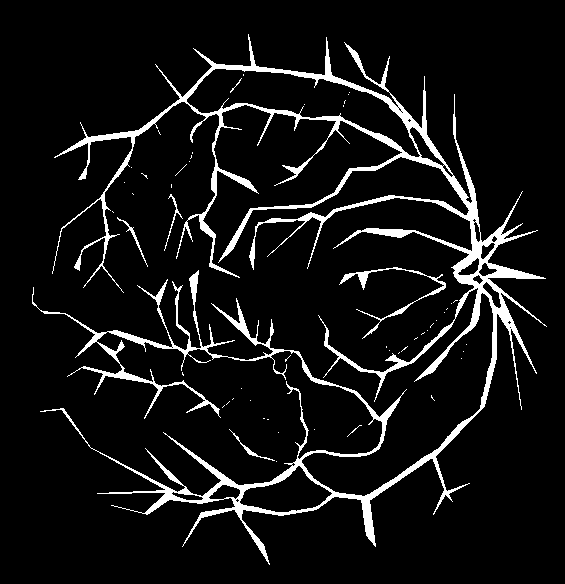

Supplement: Supplementary file 1 — Additional file 1. Generated noisy label maps. [file 12880_2021_732_MOESM1_ESM.zip › Noisy_label_maps/DRIVE(R)/LV2/17_manual1.png]

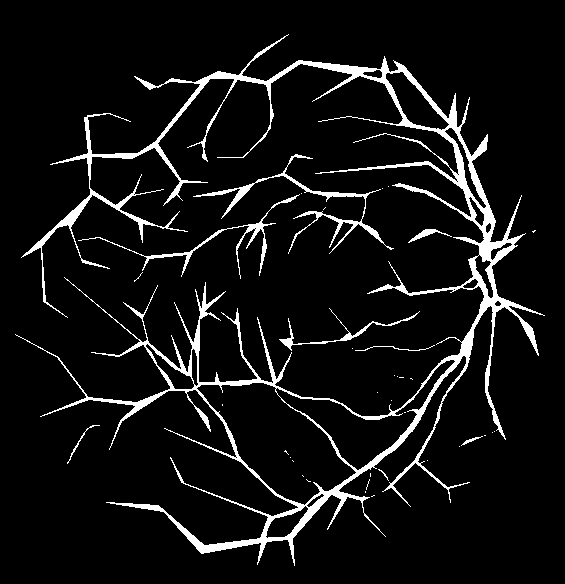

Supplement: Supplementary file 1 — Additional file 1. Generated noisy label maps. [file 12880_2021_732_MOESM1_ESM.zip › Noisy_label_maps/DRIVE(R)/LV2/18_manual1.png]

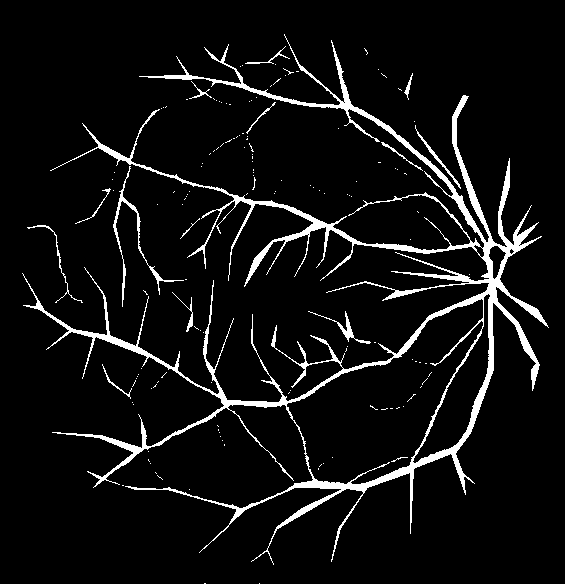

Supplement: Supplementary file 1 — Additional file 1. Generated noisy label maps. [file 12880_2021_732_MOESM1_ESM.zip › Noisy_label_maps/DRIVE(R)/LV2/19_manual1.png]

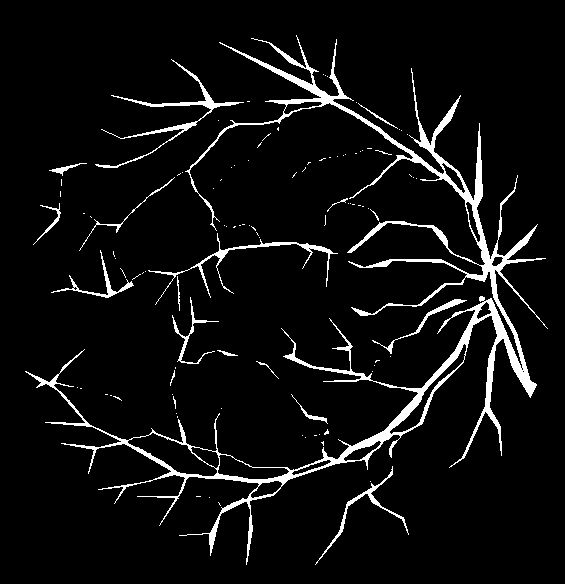

Supplement: Supplementary file 1 — Additional file 1. Generated noisy label maps. [file 12880_2021_732_MOESM1_ESM.zip › Noisy_label_maps/DRIVE(R)/LV2/20_manual1.png]

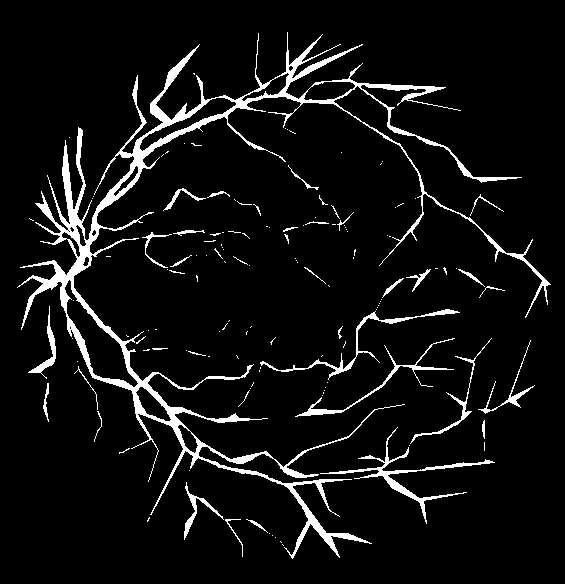

Supplement: Supplementary file 1 — Additional file 1. Generated noisy label maps. [file 12880_2021_732_MOESM1_ESM.zip › Noisy_label_maps/DRIVE(R)/LV3/01_manual1.png]

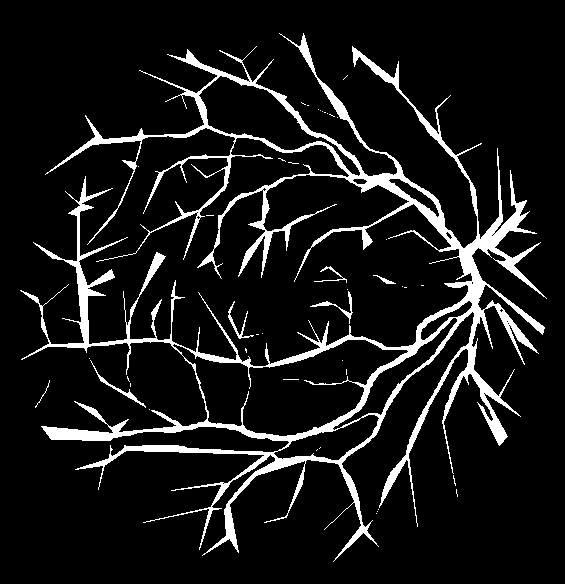

Supplement: Supplementary file 1 — Additional file 1. Generated noisy label maps. [file 12880_2021_732_MOESM1_ESM.zip › Noisy_label_maps/DRIVE(R)/LV3/02_manual1.png]

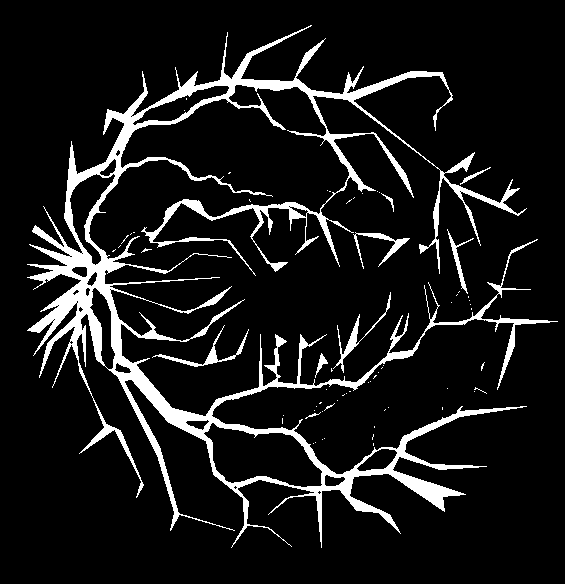

Supplement: Supplementary file 1 — Additional file 1. Generated noisy label maps. [file 12880_2021_732_MOESM1_ESM.zip › Noisy_label_maps/DRIVE(R)/LV3/03_manual1.png]

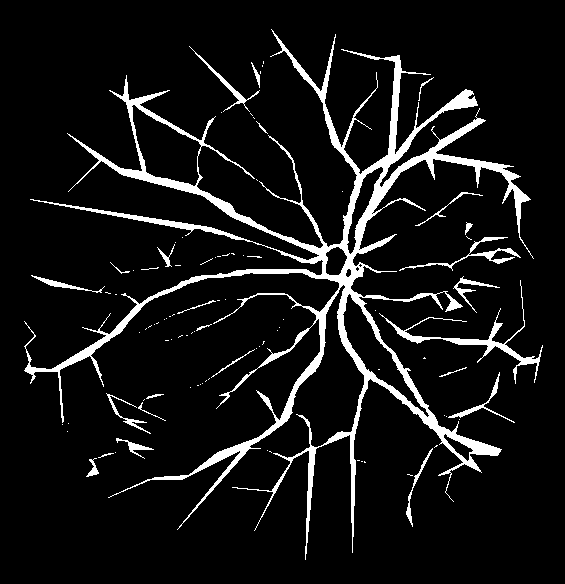

Supplement: Supplementary file 1 — Additional file 1. Generated noisy label maps. [file 12880_2021_732_MOESM1_ESM.zip › Noisy_label_maps/DRIVE(R)/LV3/04_manual1.png]

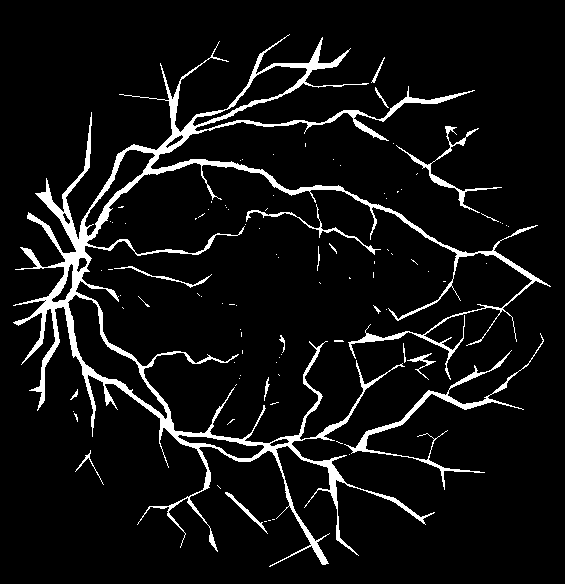

Supplement: Supplementary file 1 — Additional file 1. Generated noisy label maps. [file 12880_2021_732_MOESM1_ESM.zip › Noisy_label_maps/DRIVE(R)/LV3/05_manual1.png]

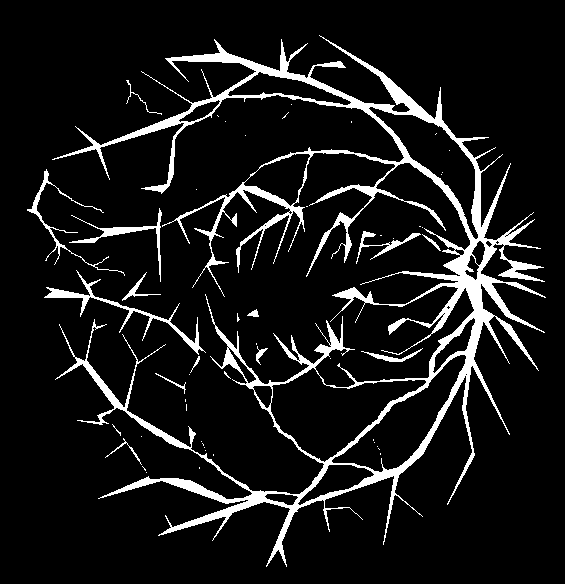

Supplement: Supplementary file 1 — Additional file 1. Generated noisy label maps. [file 12880_2021_732_MOESM1_ESM.zip › Noisy_label_maps/DRIVE(R)/LV3/06_manual1.png]

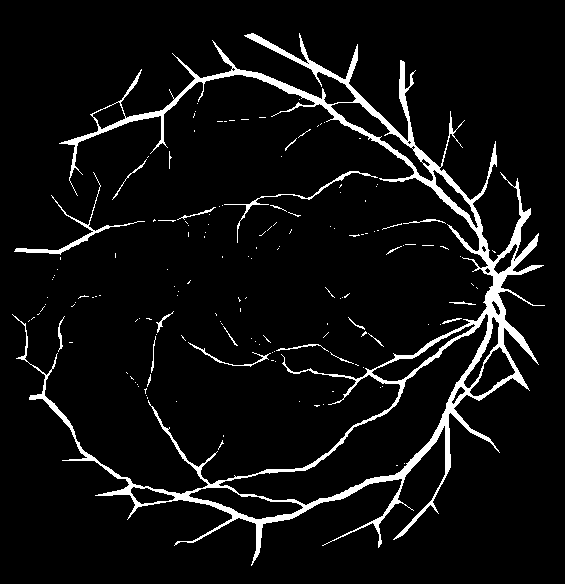

Supplement: Supplementary file 1 — Additional file 1. Generated noisy label maps. [file 12880_2021_732_MOESM1_ESM.zip › Noisy_label_maps/DRIVE(R)/LV3/07_manual1.png]

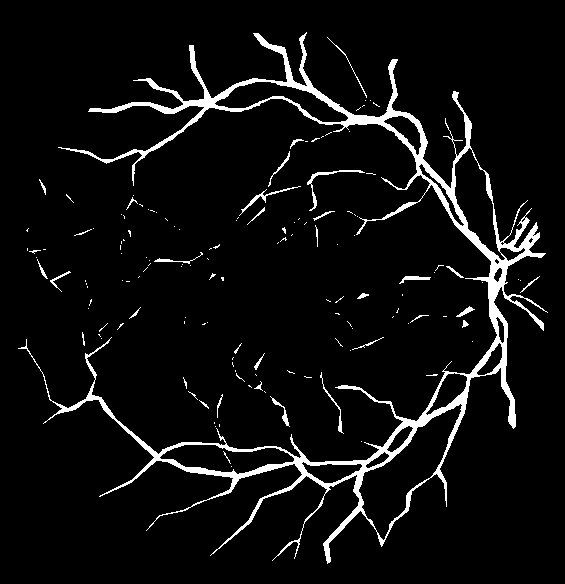

Supplement: Supplementary file 1 — Additional file 1. Generated noisy label maps. [file 12880_2021_732_MOESM1_ESM.zip › Noisy_label_maps/DRIVE(R)/LV3/08_manual1.png]

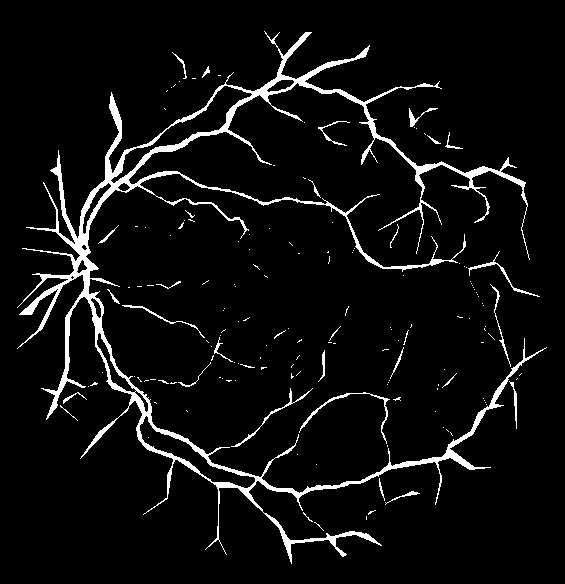

Supplement: Supplementary file 1 — Additional file 1. Generated noisy label maps. [file 12880_2021_732_MOESM1_ESM.zip › Noisy_label_maps/DRIVE(R)/LV3/09_manual1.png]

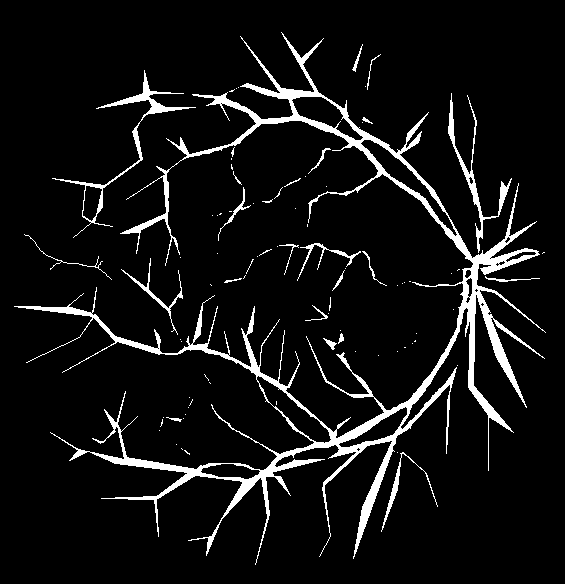

Supplement: Supplementary file 1 — Additional file 1. Generated noisy label maps. [file 12880_2021_732_MOESM1_ESM.zip › Noisy_label_maps/DRIVE(R)/LV3/10_manual1.png]

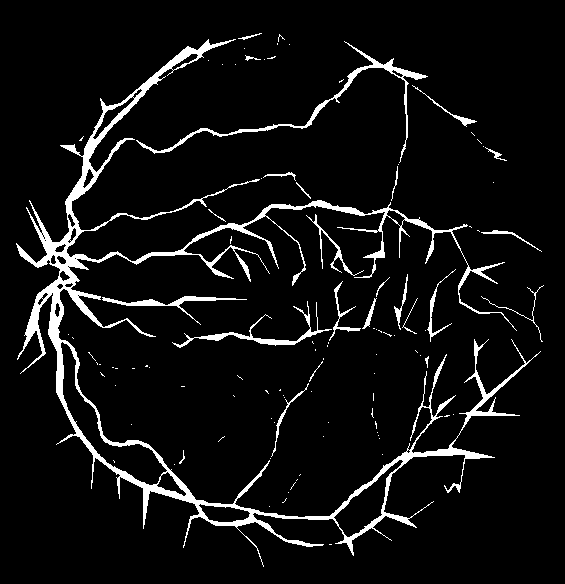

Supplement: Supplementary file 1 — Additional file 1. Generated noisy label maps. [file 12880_2021_732_MOESM1_ESM.zip › Noisy_label_maps/DRIVE(R)/LV3/11_manual1.png]

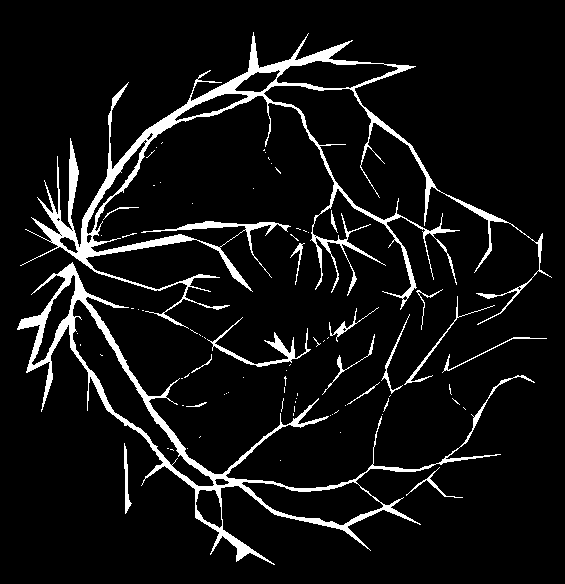

Supplement: Supplementary file 1 — Additional file 1. Generated noisy label maps. [file 12880_2021_732_MOESM1_ESM.zip › Noisy_label_maps/DRIVE(R)/LV3/12_manual1.png]

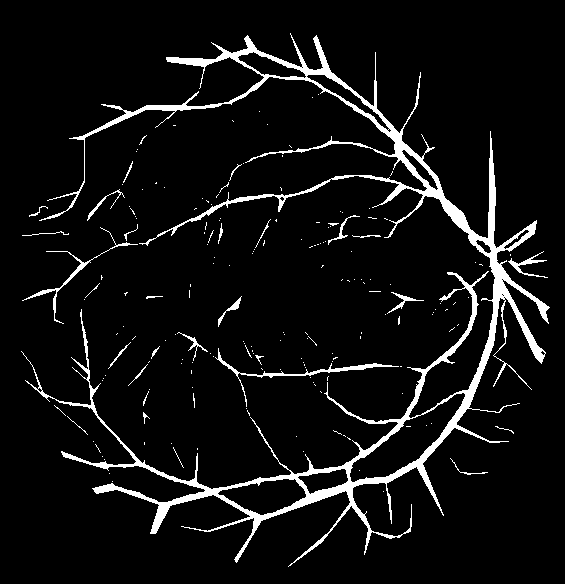

Supplement: Supplementary file 1 — Additional file 1. Generated noisy label maps. [file 12880_2021_732_MOESM1_ESM.zip › Noisy_label_maps/DRIVE(R)/LV3/13_manual1.png]

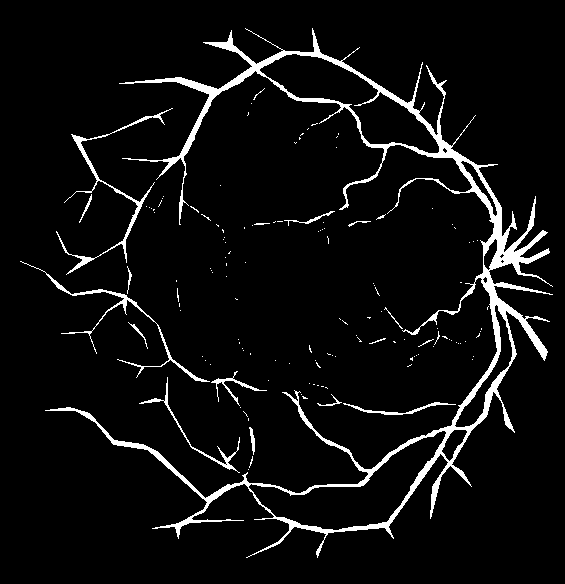

Supplement: Supplementary file 1 — Additional file 1. Generated noisy label maps. [file 12880_2021_732_MOESM1_ESM.zip › Noisy_label_maps/DRIVE(R)/LV3/14_manual1.png]

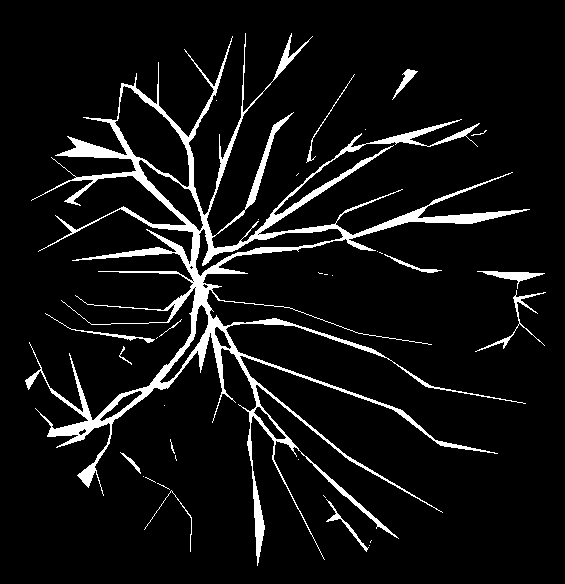

Supplement: Supplementary file 1 — Additional file 1. Generated noisy label maps. [file 12880_2021_732_MOESM1_ESM.zip › Noisy_label_maps/DRIVE(R)/LV3/15_manual1.png]

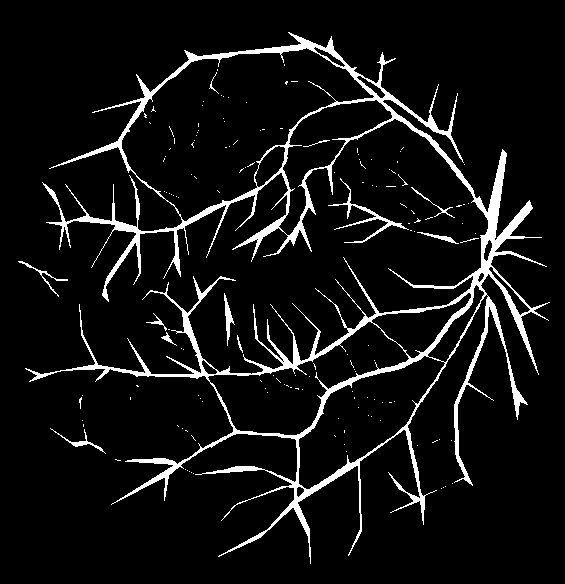

Supplement: Supplementary file 1 — Additional file 1. Generated noisy label maps. [file 12880_2021_732_MOESM1_ESM.zip › Noisy_label_maps/DRIVE(R)/LV3/16_manual1.png]

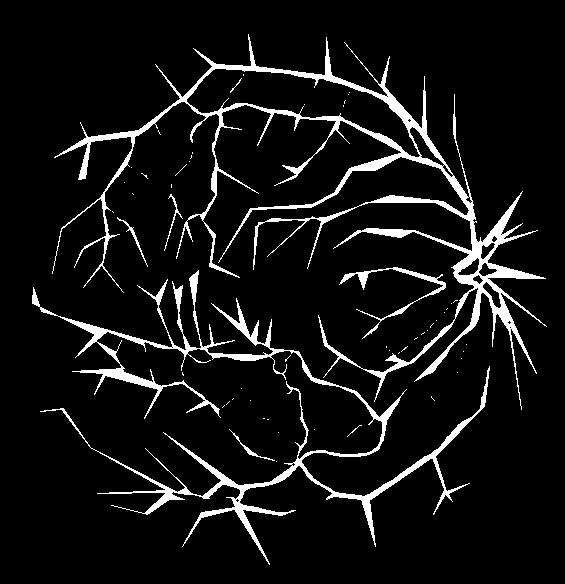

Supplement: Supplementary file 1 — Additional file 1. Generated noisy label maps. [file 12880_2021_732_MOESM1_ESM.zip › Noisy_label_maps/DRIVE(R)/LV3/17_manual1.png]

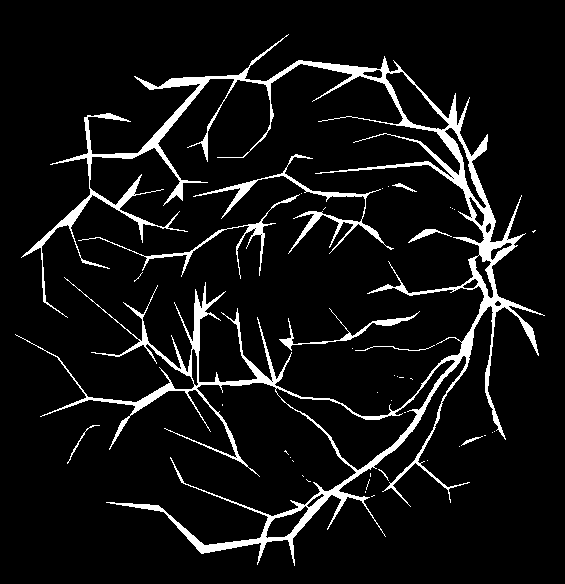

Supplement: Supplementary file 1 — Additional file 1. Generated noisy label maps. [file 12880_2021_732_MOESM1_ESM.zip › Noisy_label_maps/DRIVE(R)/LV3/18_manual1.png]

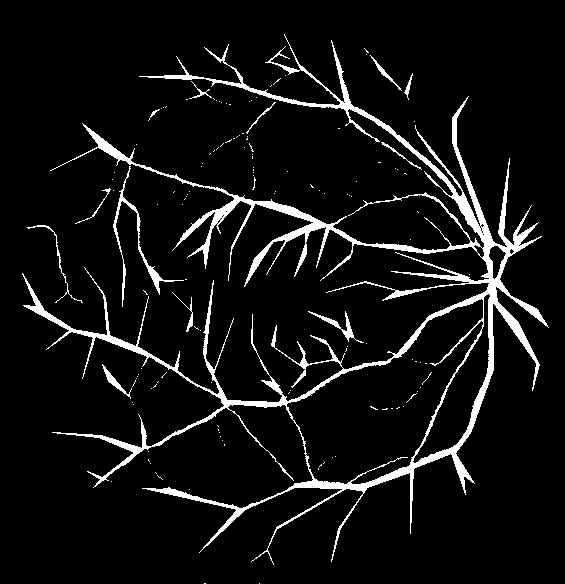

Supplement: Supplementary file 1 — Additional file 1. Generated noisy label maps. [file 12880_2021_732_MOESM1_ESM.zip › Noisy_label_maps/DRIVE(R)/LV3/19_manual1.png]

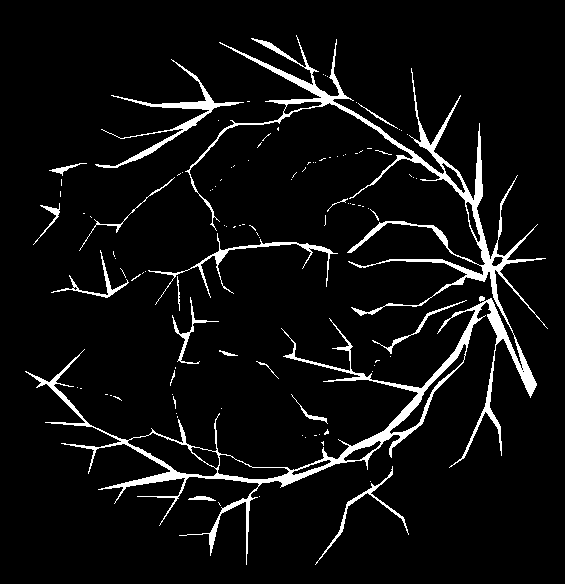

Supplement: Supplementary file 1 — Additional file 1. Generated noisy label maps. [file 12880_2021_732_MOESM1_ESM.zip › Noisy_label_maps/DRIVE(R)/LV3/20_manual1.png]

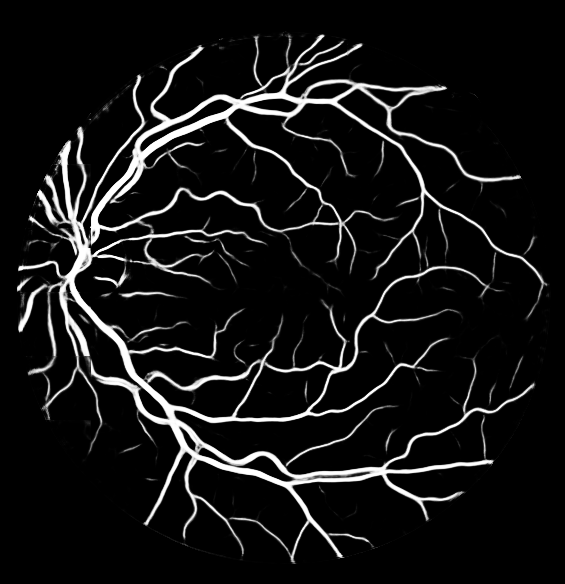

Supplement: Supplementary file 1 — Additional file 1. Generated noisy label maps. [file 12880_2021_732_MOESM1_ESM.zip › Noisy_label_maps/DRIVE(R)/Pseudo/01_prediction_mix.png]

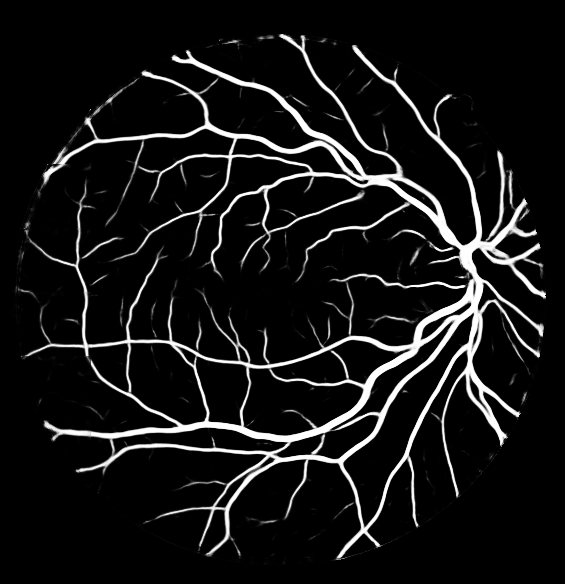

Supplement: Supplementary file 1 — Additional file 1. Generated noisy label maps. [file 12880_2021_732_MOESM1_ESM.zip › Noisy_label_maps/DRIVE(R)/Pseudo/02_prediction_mix.png]

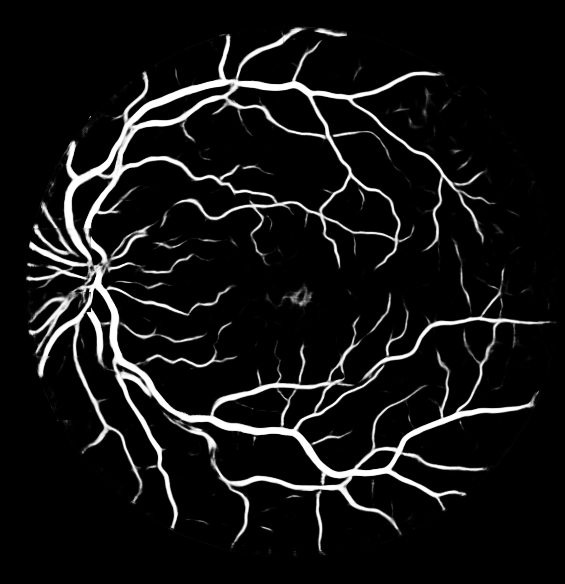

Supplement: Supplementary file 1 — Additional file 1. Generated noisy label maps. [file 12880_2021_732_MOESM1_ESM.zip › Noisy_label_maps/DRIVE(R)/Pseudo/03_prediction_mix.png]

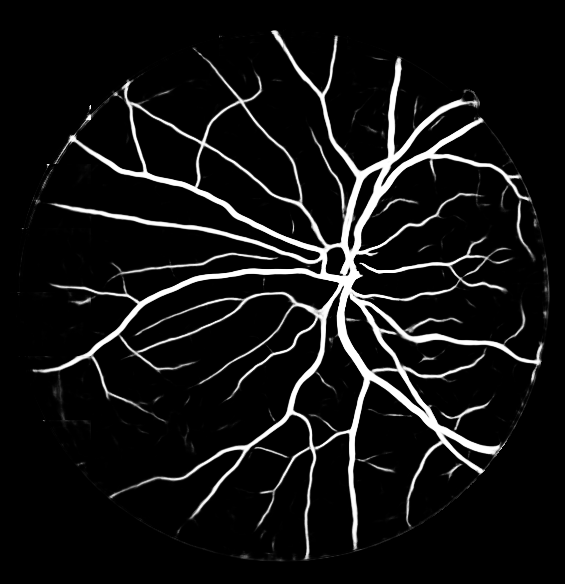

Supplement: Supplementary file 1 — Additional file 1. Generated noisy label maps. [file 12880_2021_732_MOESM1_ESM.zip › Noisy_label_maps/DRIVE(R)/Pseudo/04_prediction_mix.png]

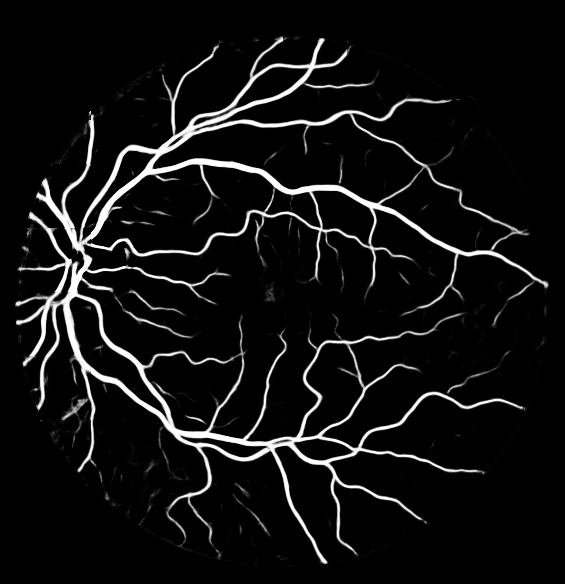

Supplement: Supplementary file 1 — Additional file 1. Generated noisy label maps. [file 12880_2021_732_MOESM1_ESM.zip › Noisy_label_maps/DRIVE(R)/Pseudo/05_prediction_mix.png]

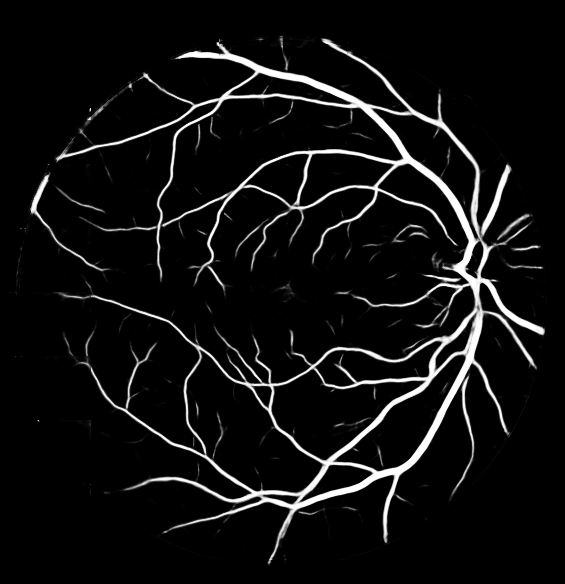

Supplement: Supplementary file 1 — Additional file 1. Generated noisy label maps. [file 12880_2021_732_MOESM1_ESM.zip › Noisy_label_maps/DRIVE(R)/Pseudo/06_prediction_mix.png]

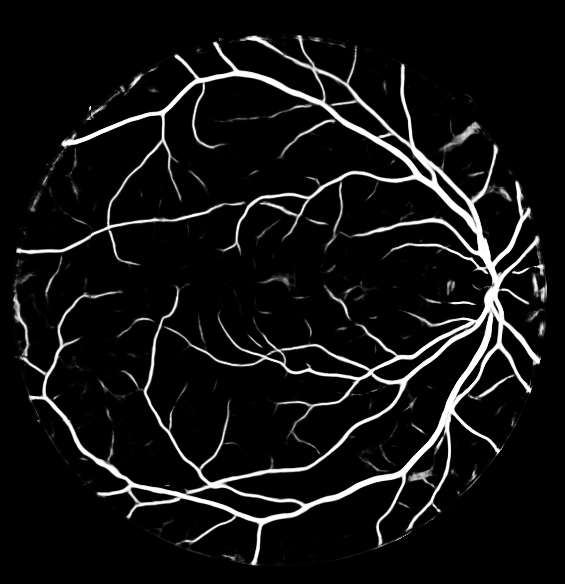

Supplement: Supplementary file 1 — Additional file 1. Generated noisy label maps. [file 12880_2021_732_MOESM1_ESM.zip › Noisy_label_maps/DRIVE(R)/Pseudo/07_prediction_mix.png]

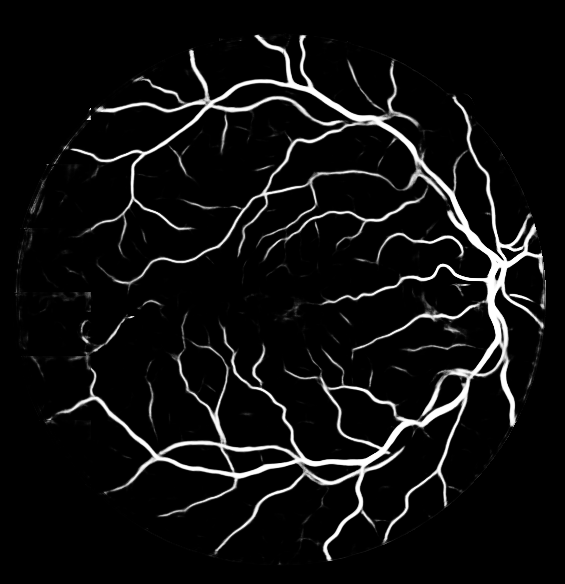

Supplement: Supplementary file 1 — Additional file 1. Generated noisy label maps. [file 12880_2021_732_MOESM1_ESM.zip › Noisy_label_maps/DRIVE(R)/Pseudo/08_prediction_mix.png]

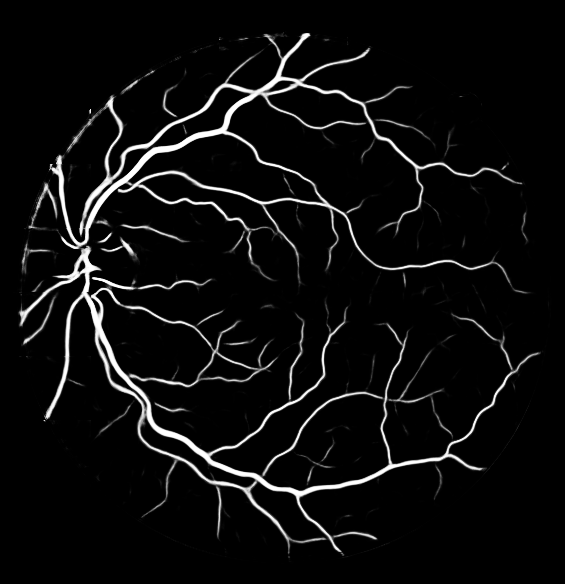

Supplement: Supplementary file 1 — Additional file 1. Generated noisy label maps. [file 12880_2021_732_MOESM1_ESM.zip › Noisy_label_maps/DRIVE(R)/Pseudo/09_prediction_mix.png]

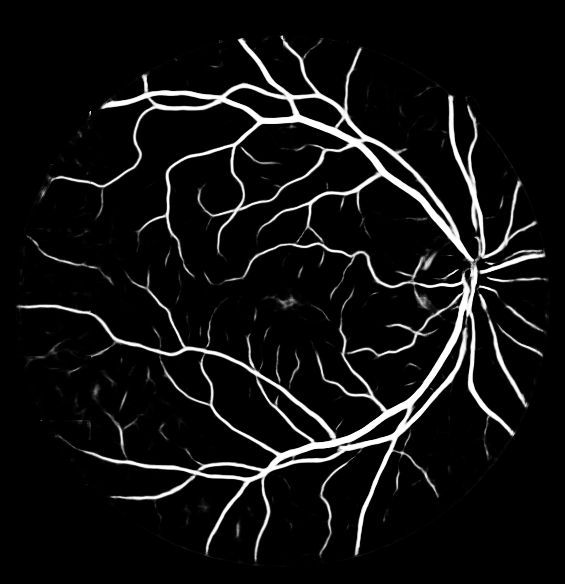

Supplement: Supplementary file 1 — Additional file 1. Generated noisy label maps. [file 12880_2021_732_MOESM1_ESM.zip › Noisy_label_maps/DRIVE(R)/Pseudo/10_prediction_mix.png]

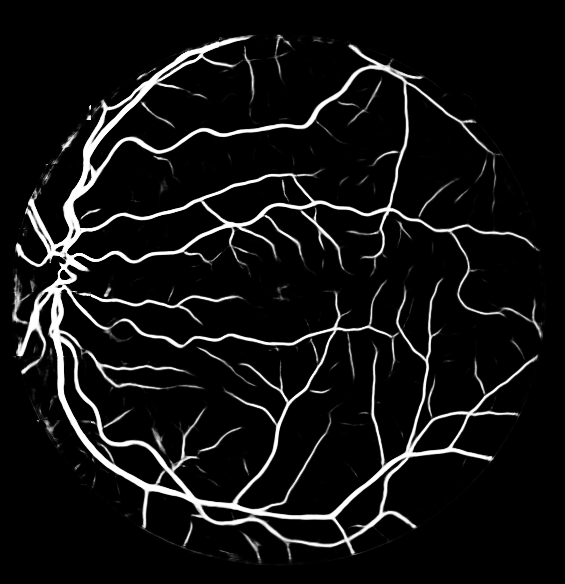

Supplement: Supplementary file 1 — Additional file 1. Generated noisy label maps. [file 12880_2021_732_MOESM1_ESM.zip › Noisy_label_maps/DRIVE(R)/Pseudo/11_prediction_mix.png]

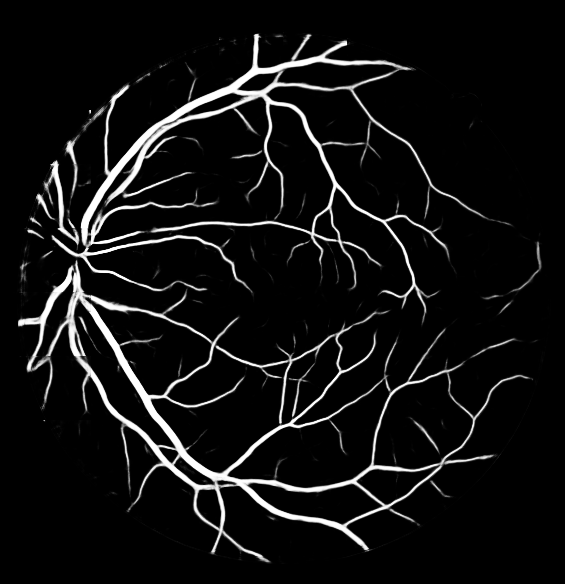

Supplement: Supplementary file 1 — Additional file 1. Generated noisy label maps. [file 12880_2021_732_MOESM1_ESM.zip › Noisy_label_maps/DRIVE(R)/Pseudo/12_prediction_mix.png]

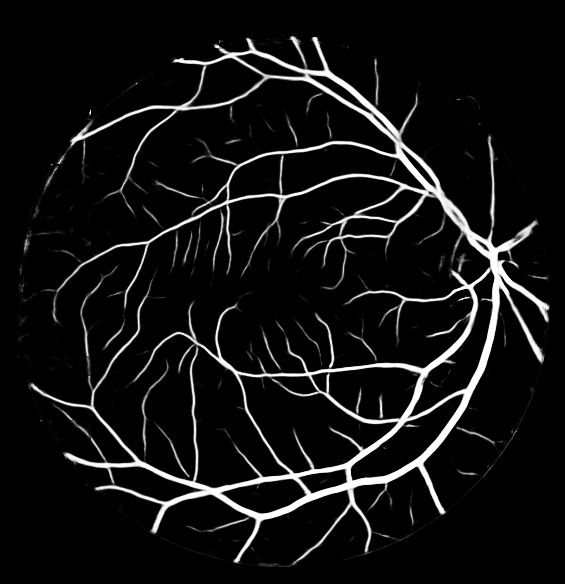

Supplement: Supplementary file 1 — Additional file 1. Generated noisy label maps. [file 12880_2021_732_MOESM1_ESM.zip › Noisy_label_maps/DRIVE(R)/Pseudo/13_prediction_mix.png]

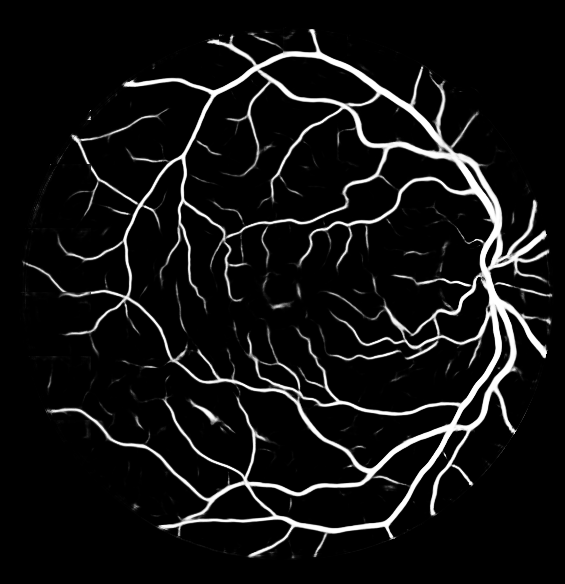

Supplement: Supplementary file 1 — Additional file 1. Generated noisy label maps. [file 12880_2021_732_MOESM1_ESM.zip › Noisy_label_maps/DRIVE(R)/Pseudo/14_prediction_mix.png]

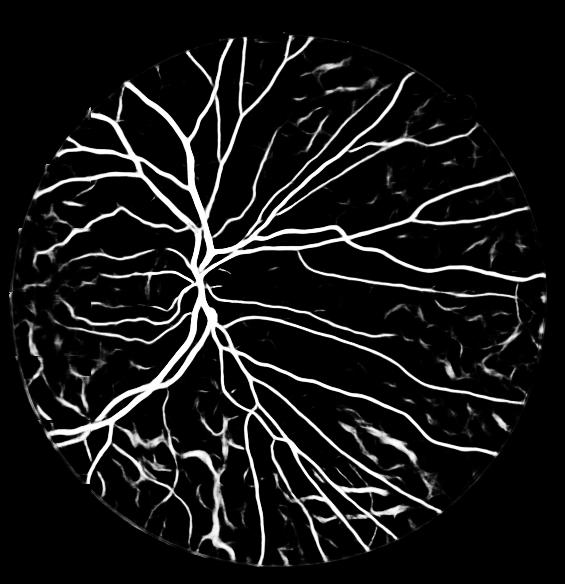

Supplement: Supplementary file 1 — Additional file 1. Generated noisy label maps. [file 12880_2021_732_MOESM1_ESM.zip › Noisy_label_maps/DRIVE(R)/Pseudo/15_prediction_mix.png]

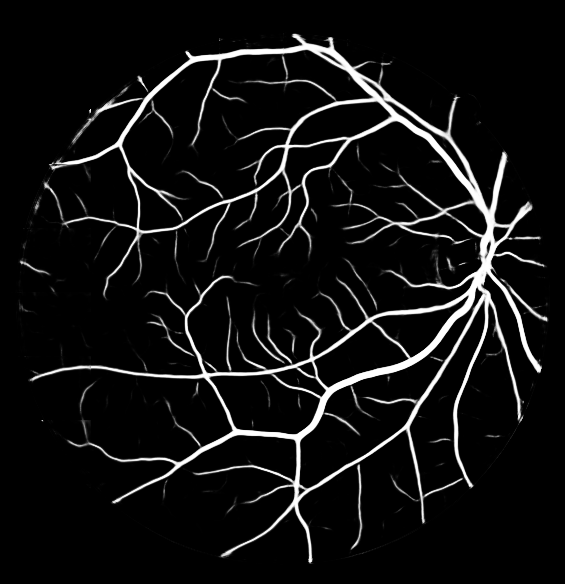

Supplement: Supplementary file 1 — Additional file 1. Generated noisy label maps. [file 12880_2021_732_MOESM1_ESM.zip › Noisy_label_maps/DRIVE(R)/Pseudo/16_prediction_mix.png]

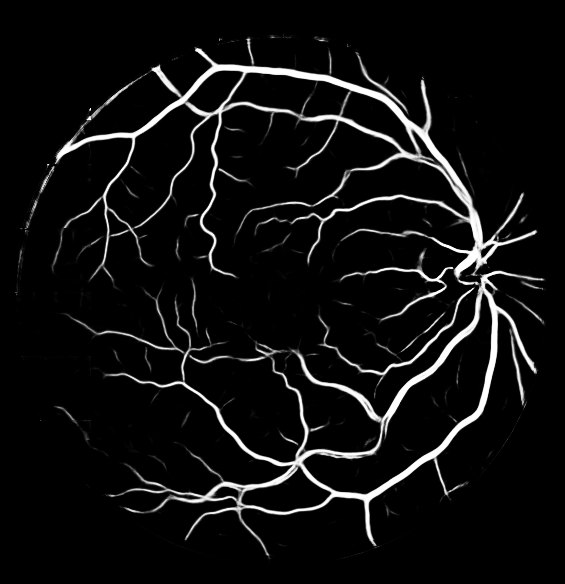

Supplement: Supplementary file 1 — Additional file 1. Generated noisy label maps. [file 12880_2021_732_MOESM1_ESM.zip › Noisy_label_maps/DRIVE(R)/Pseudo/17_prediction_mix.png]

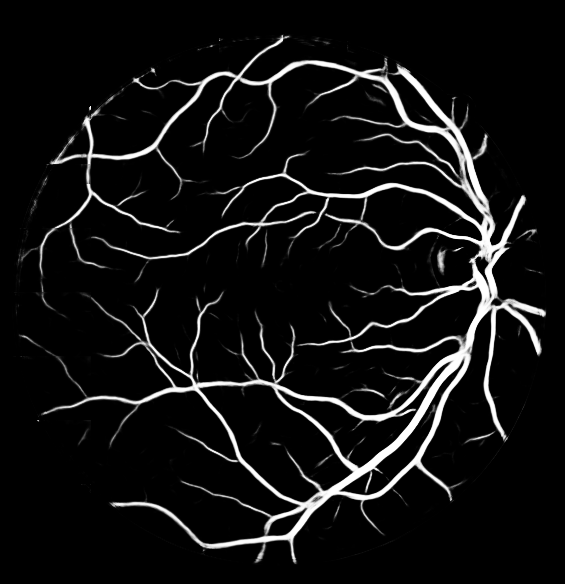

Supplement: Supplementary file 1 — Additional file 1. Generated noisy label maps. [file 12880_2021_732_MOESM1_ESM.zip › Noisy_label_maps/DRIVE(R)/Pseudo/18_prediction_mix.png]

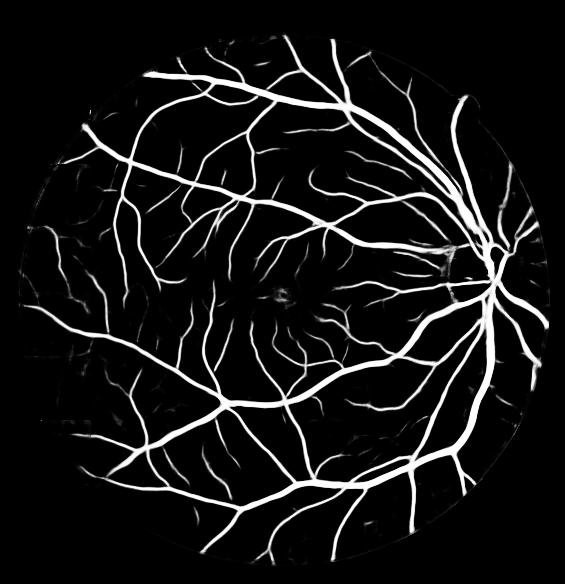

Supplement: Supplementary file 1 — Additional file 1. Generated noisy label maps. [file 12880_2021_732_MOESM1_ESM.zip › Noisy_label_maps/DRIVE(R)/Pseudo/19_prediction_mix.png]

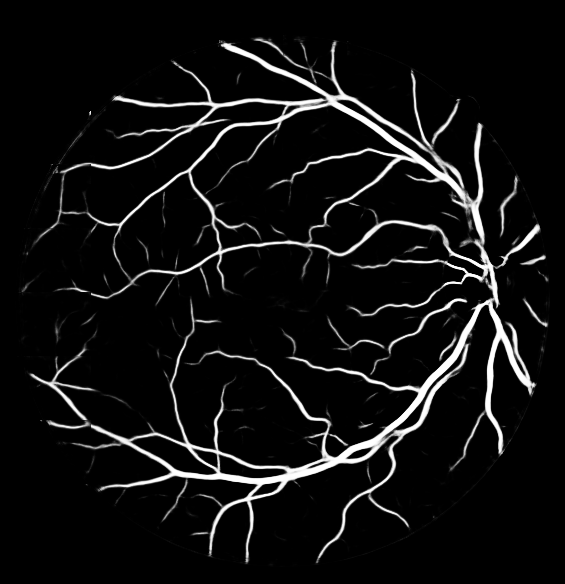

Supplement: Supplementary file 1 — Additional file 1. Generated noisy label maps. [file 12880_2021_732_MOESM1_ESM.zip › Noisy_label_maps/DRIVE(R)/Pseudo/20_prediction_mix.png]

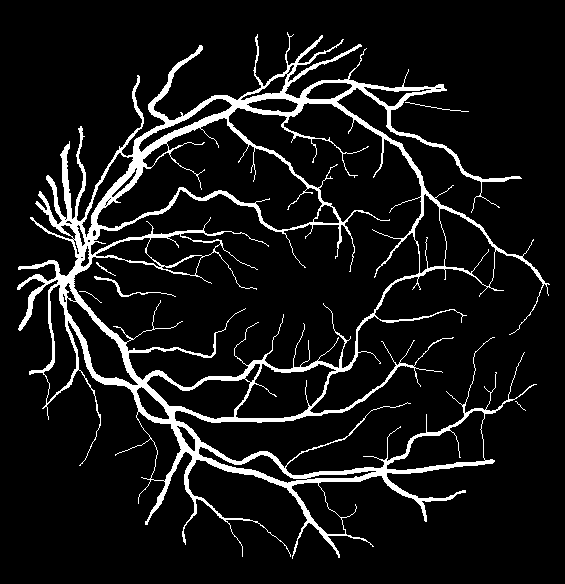

Supplement: Supplementary file 1 — Additional file 1. Generated noisy label maps. [file 12880_2021_732_MOESM1_ESM.zip › Noisy_label_maps/DRIVE(R)/Manual/01_manual1.png]

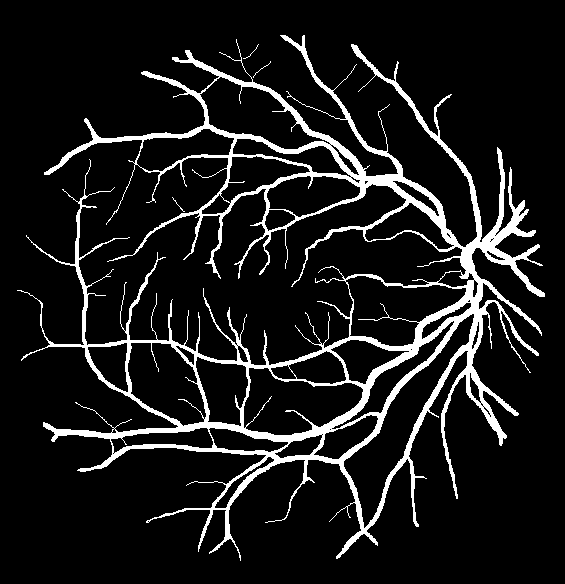

Supplement: Supplementary file 1 — Additional file 1. Generated noisy label maps. [file 12880_2021_732_MOESM1_ESM.zip › Noisy_label_maps/DRIVE(R)/Manual/02_manual1.png]

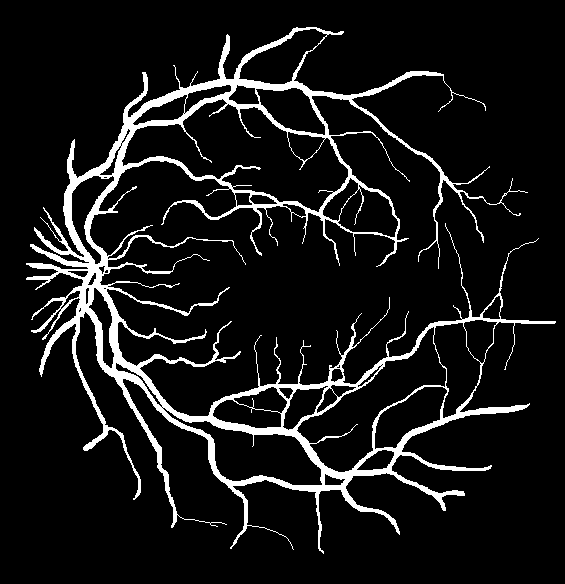

Supplement: Supplementary file 1 — Additional file 1. Generated noisy label maps. [file 12880_2021_732_MOESM1_ESM.zip › Noisy_label_maps/DRIVE(R)/Manual/03_manual1.png]

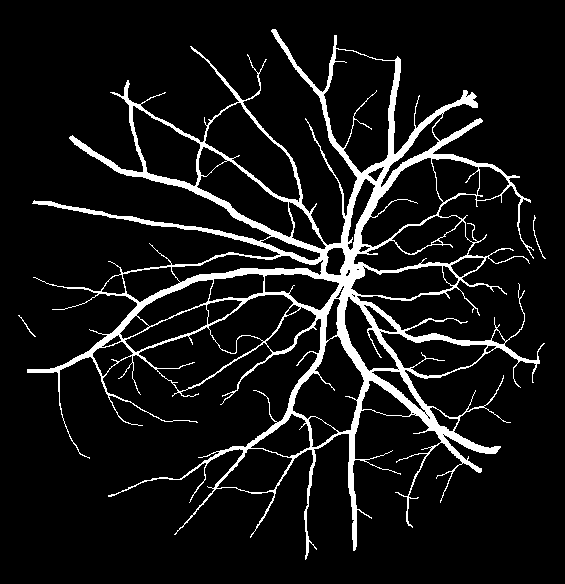

Supplement: Supplementary file 1 — Additional file 1. Generated noisy label maps. [file 12880_2021_732_MOESM1_ESM.zip › Noisy_label_maps/DRIVE(R)/Manual/04_manual1.png]

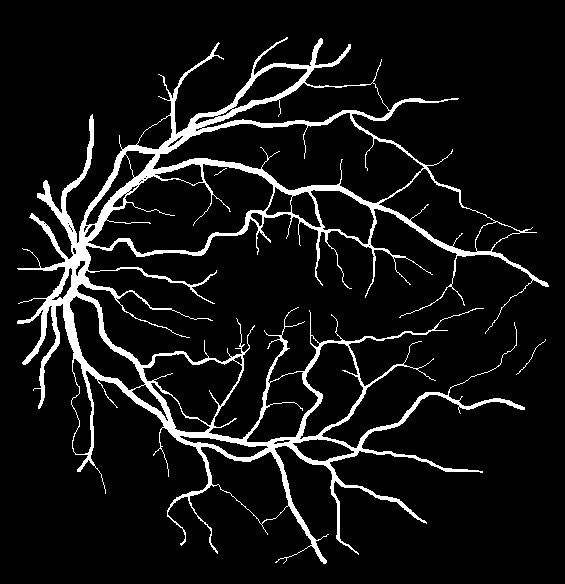

Supplement: Supplementary file 1 — Additional file 1. Generated noisy label maps. [file 12880_2021_732_MOESM1_ESM.zip › Noisy_label_maps/DRIVE(R)/Manual/05_manual1.png]

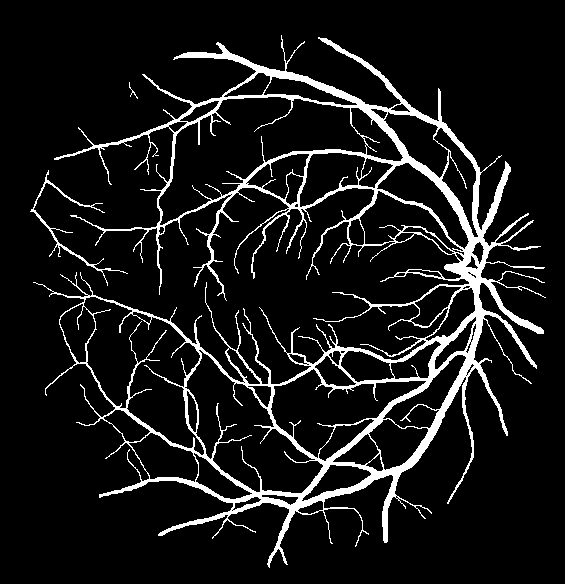

Supplement: Supplementary file 1 — Additional file 1. Generated noisy label maps. [file 12880_2021_732_MOESM1_ESM.zip › Noisy_label_maps/DRIVE(R)/Manual/06_manual1.png]

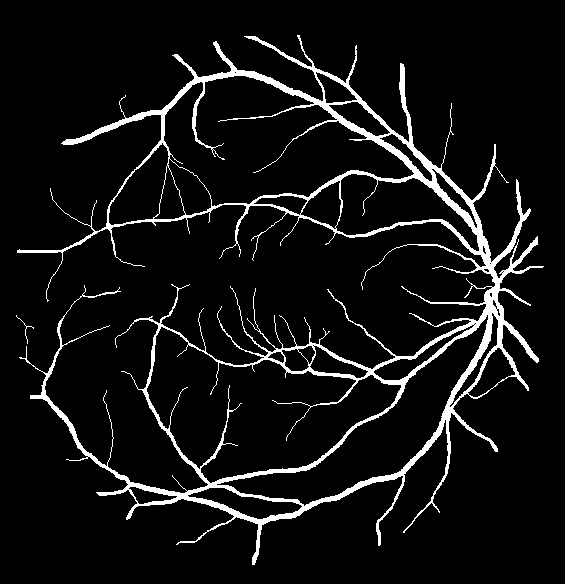

Supplement: Supplementary file 1 — Additional file 1. Generated noisy label maps. [file 12880_2021_732_MOESM1_ESM.zip › Noisy_label_maps/DRIVE(R)/Manual/07_manual1.png]

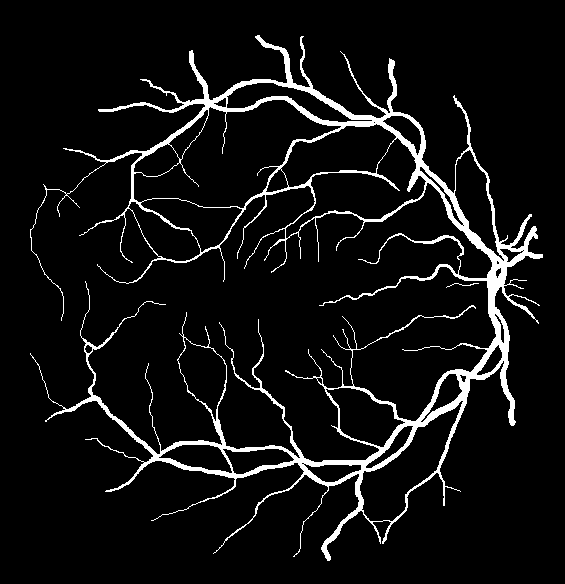

Supplement: Supplementary file 1 — Additional file 1. Generated noisy label maps. [file 12880_2021_732_MOESM1_ESM.zip › Noisy_label_maps/DRIVE(R)/Manual/08_manual1.png]

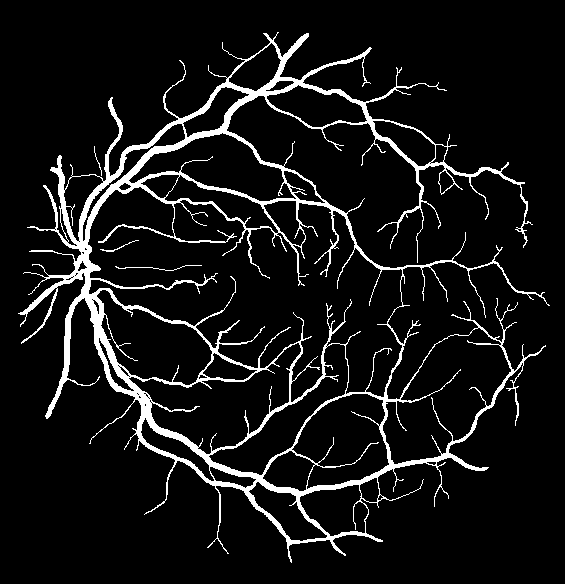

Supplement: Supplementary file 1 — Additional file 1. Generated noisy label maps. [file 12880_2021_732_MOESM1_ESM.zip › Noisy_label_maps/DRIVE(R)/Manual/09_manual1.png]

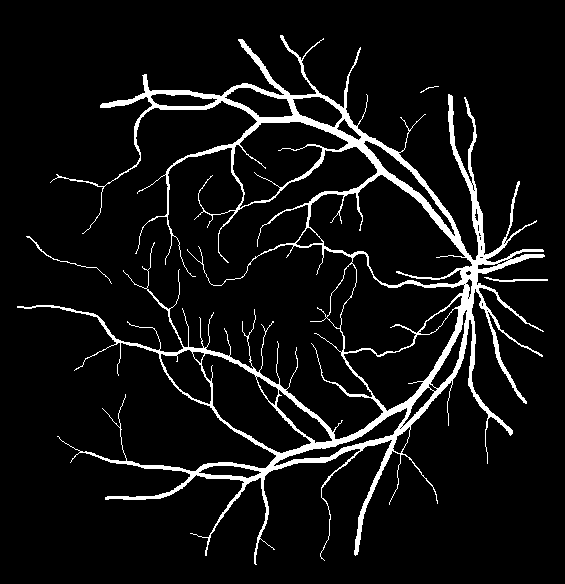

Supplement: Supplementary file 1 — Additional file 1. Generated noisy label maps. [file 12880_2021_732_MOESM1_ESM.zip › Noisy_label_maps/DRIVE(R)/Manual/10_manual1.png]

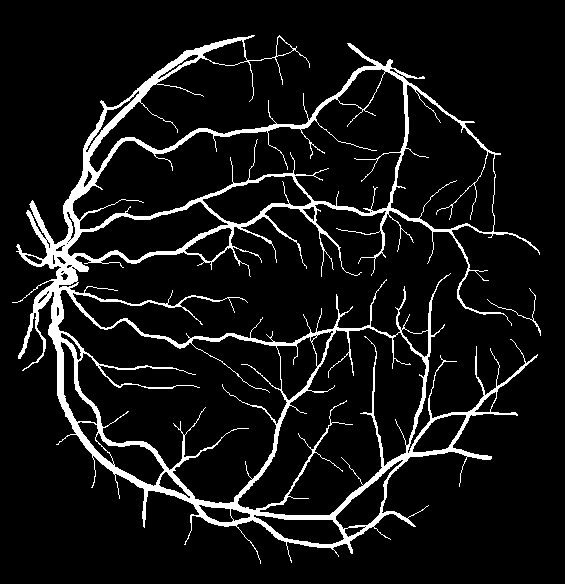

Supplement: Supplementary file 1 — Additional file 1. Generated noisy label maps. [file 12880_2021_732_MOESM1_ESM.zip › Noisy_label_maps/DRIVE(R)/Manual/11_manual1.png]

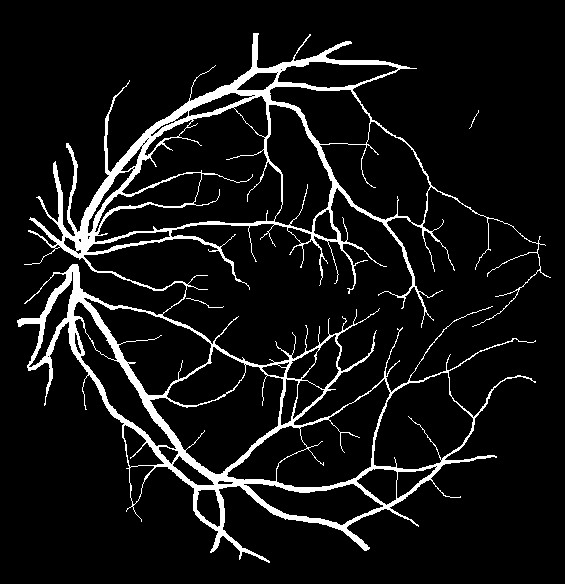

Supplement: Supplementary file 1 — Additional file 1. Generated noisy label maps. [file 12880_2021_732_MOESM1_ESM.zip › Noisy_label_maps/DRIVE(R)/Manual/12_manual1.png]

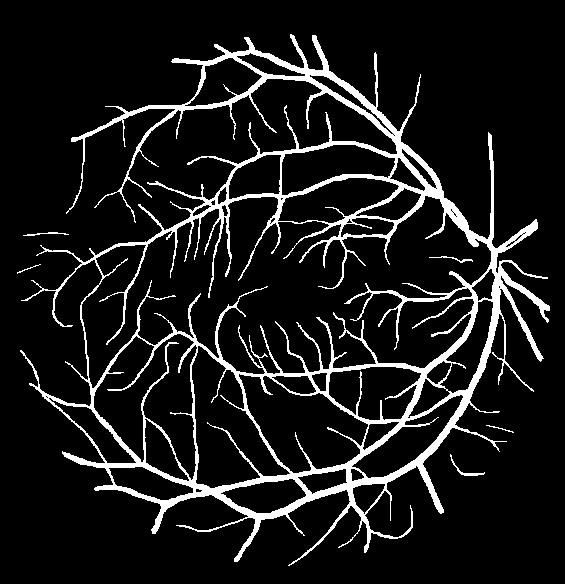

Supplement: Supplementary file 1 — Additional file 1. Generated noisy label maps. [file 12880_2021_732_MOESM1_ESM.zip › Noisy_label_maps/DRIVE(R)/Manual/13_manual1.png]

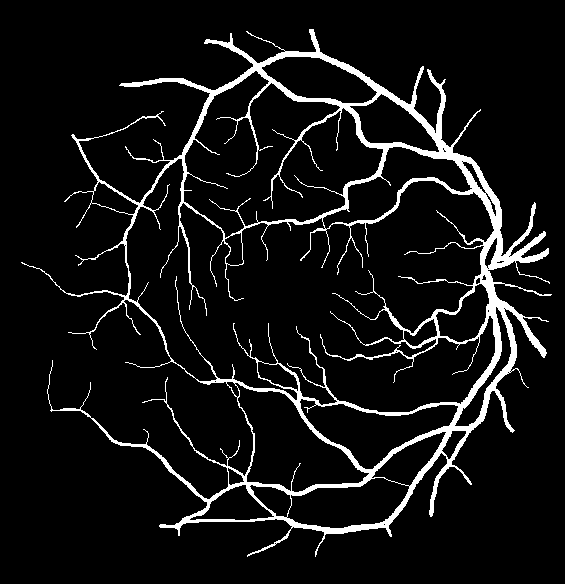

Supplement: Supplementary file 1 — Additional file 1. Generated noisy label maps. [file 12880_2021_732_MOESM1_ESM.zip › Noisy_label_maps/DRIVE(R)/Manual/14_manual1.png]

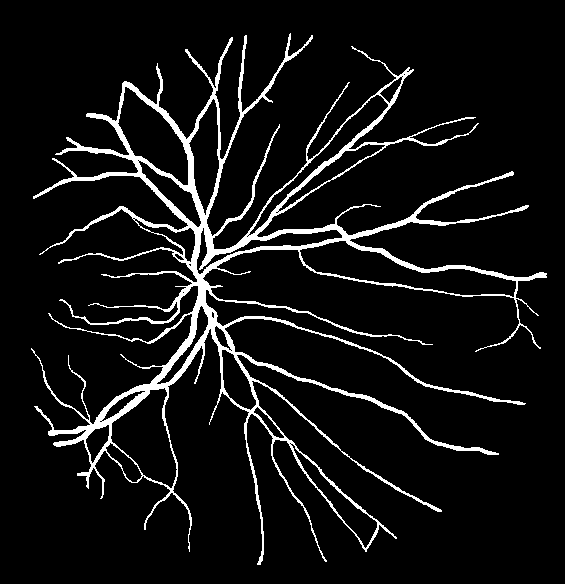

Supplement: Supplementary file 1 — Additional file 1. Generated noisy label maps. [file 12880_2021_732_MOESM1_ESM.zip › Noisy_label_maps/DRIVE(R)/Manual/15_manual1.png]

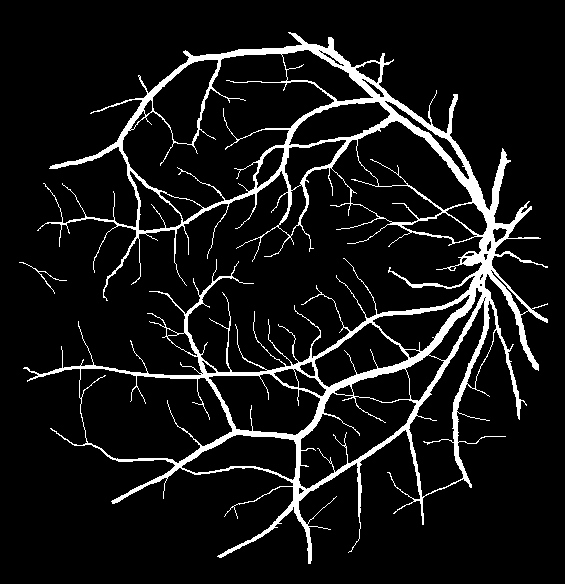

Supplement: Supplementary file 1 — Additional file 1. Generated noisy label maps. [file 12880_2021_732_MOESM1_ESM.zip › Noisy_label_maps/DRIVE(R)/Manual/16_manual1.png]

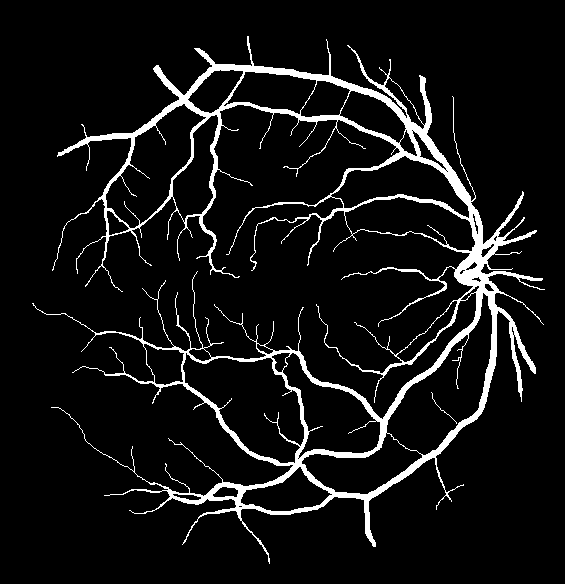

Supplement: Supplementary file 1 — Additional file 1. Generated noisy label maps. [file 12880_2021_732_MOESM1_ESM.zip › Noisy_label_maps/DRIVE(R)/Manual/17_manual1.png]

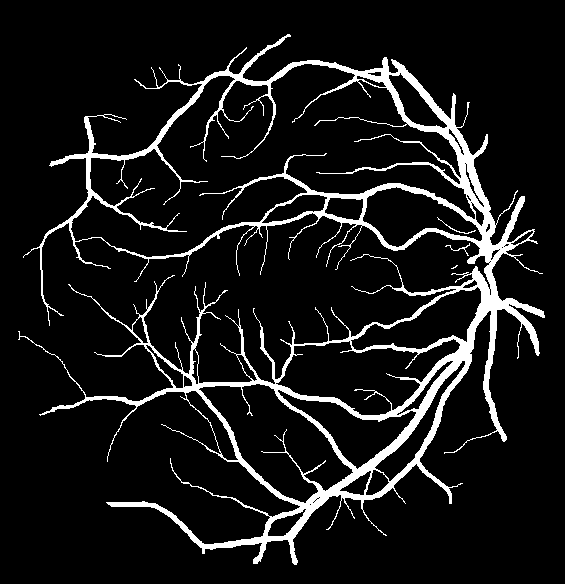

Supplement: Supplementary file 1 — Additional file 1. Generated noisy label maps. [file 12880_2021_732_MOESM1_ESM.zip › Noisy_label_maps/DRIVE(R)/Manual/18_manual1.png]

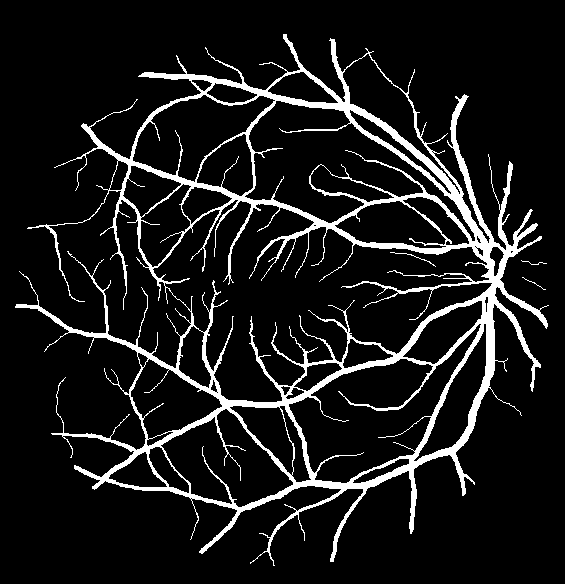

Supplement: Supplementary file 1 — Additional file 1. Generated noisy label maps. [file 12880_2021_732_MOESM1_ESM.zip › Noisy_label_maps/DRIVE(R)/Manual/19_manual1.png]

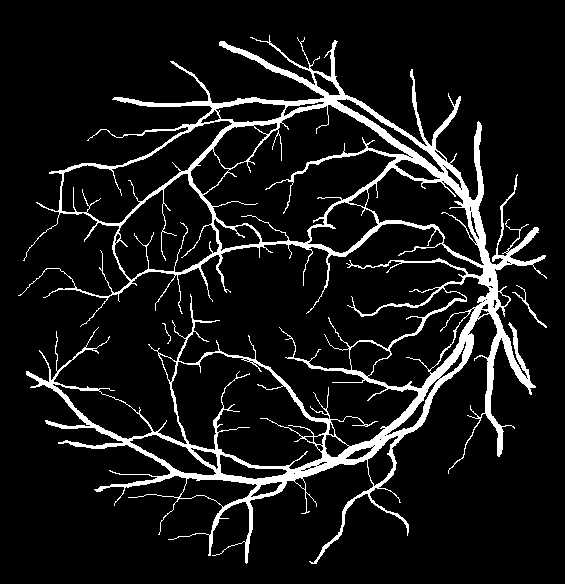

Supplement: Supplementary file 1 — Additional file 1. Generated noisy label maps. [file 12880_2021_732_MOESM1_ESM.zip › Noisy_label_maps/DRIVE(R)/Manual/20_manual1.png]
